# Comparative transcriptome analysis reveals evolutionary divergence and shared network of cold and salt stress response in diploid D-genome cotton

**DOI:** 10.1186/s12870-020-02726-4

**Published:** 2020-11-12

**Authors:** Yanchao Xu, Richard Odongo Magwanga, Dingsha Jin, Xiaoyan Cai, Yuqing Hou, Zheng Juyun, Stephen Gaya Agong, Kunbo Wang, Fang Liu, Zhongli Zhou

**Affiliations:** 1 State Key Laboratory of Cotton Biology, Institute of Cotton Research, Chinese Academy of Agricultural Sciences, Anyang, 455000 China; 2 College of Plant Science and Technology, Huazhong Agricultural University, Wuhan, 40070 China; 3 School of Biological, Physical, Mathematics and Actuarial sciences (SBPMAS), Main campus, Jaramogi Oginga Odinga University of Science and Technology (JOOUST), P.O Box 210-40601, Bondo, Kenya; 4 Economic Crops Research Institute of Xinjiang Academy of Agricultural Science, Urumqi, Xinjiang province China; 5 School of Agricultural Sciences, Zhengzhou University, Zhengzhou, 450001 Henan China

**Keywords:** Diploid D-genome cotton, Co-expression, Comparative transcriptome, Evolutionary divergence, Shared network

## Abstract

**Background:**

Wild species of cotton are excellent resistance to abiotic stress. Diploid D-genome cotton showed abundant phenotypic diversity and was the putative donor species of allotetraploid cotton which produce the largest textile natural fiber.

**Results:**

A total of 41,053 genes were expressed in all samples by mapping RNA-seq Illumina reads of *G. thurberi* (D_1_), *G. klotzschianum* (D_3-k_)*, G. raimondii* (D_5_) and *G. trilobum* (D_8_) to reference genome. The numbers of differently expressed genes (DEGs) were significantly higher under cold stress than salt stress. However, 34.1% DEGs under salt stress were overlapped with cold stress in four species. Notably, a potential shared network (cold and salt response, including 16 genes) was mined out by gene co-expression analysis. A total of 47,180–55,548 unique genes were identified in four diploid species by De novo assembly. Furthermore, 163, 344, 330, and 161 positively selected genes (PSGs) were detected in *thurberi*, *G. klotzschianum*, *G. raimondii* and *G. trilobum* by evolutionary analysis, respectively, and 9.5–17% PSGs of four species were DEGs in corresponding species under cold or salt stress. What’s more, most of PSGs were enriched GO term related to response to stimulation. *G. klotzschianum* showed *the* best tolerance under both cold and salt stress. Interestingly, we found that a RALF-like protein coding gene not only is PSGs of *G. klotzschianum,* but also belongs to the potential shared network.

**Conclusion:**

Our study provided new evidence that gene expression variations of evolution by natural selection were essential drivers of the morphological variations related to environmental adaptation during evolution. Additionally, there exist shared regulated networks under cold and salt stress, such as Ca^2+^ signal transduction and oxidation-reduction mechanisms. Our work establishes a transcriptomic selection mechanism for altering gene expression of the four diploid D-genome cotton and provides available gene resource underlying multi-abiotic resistant cotton breeding strategy.

**Supplementary Information:**

The online version contains supplementary material available at 10.1186/s12870-020-02726-4.

## Background

Cotton (*Gossypium*) provides the most natural fiber for the manufacture of textiles [1], and is an important natural fiber crop globally. At present, *G. hirsutum* and *G. barbadense* (allotetraploid cotton) are widely planted, and domesticated through a long-time history. Genetic evidence suggests that allotetraploid cotton was formed by diploid A- and D-genome species hybridization events at about 1–2 million years ago [2]. Diploid D-genome cotton contains at least 13 species. Among those species, *G. thurberi*, *G. klotzschianum, G. raimondii,* and *G. trilobum*, which distribute four different latitude areas of the Americas [3], were observed distinct difference of morphological character. Molecular evolutionary processes and phylogeny of those four species were revealed through phylogenetic methods [3]. Such as, *G. thurberi* and *G. trilobum* show the close relationship of phylogeny, even though there is distinctly different latitude of natural distribution. Moreover, *G. thurberi* a member of the D genome is tolerant of cold temperature [4].

Currently, researchers acknowledges that *G. arboreum* and *G. raimondii* as the putative A and D genome donors of the elite allotetraploid cotton, respectively [5]. A-genome diploid cotton contains two species, *G. arboreum* and *G. herbaceum*, and distributed Southern Africa and Asia. D-genome diploid cotton contains about 13 species, and primarily distributed Mexico, with range extensions into Peru, the Galapagos Islands, and southern Arizona [6]. The reason behind the divergent genomic and morphological characteristics of D- and A-genome cotton is geography insulation and division. About 1–2 million years ago, A-genome diploid is hybridization with D-genome diploid cotton, and allotetraploid cotton appeared through subsequent polyploidization events [7]. This requires that A and D genome must have established physical proximity [8], but it is inconceivable to contact across the Pacific. Therefore, the origin and evolution of the modern allotetraploid cotton is marred with a lot of mysteries, although there are many hypotheses or theories proposed to explain the origin and evolution of the AD cotton genome.

Cold and salt stresses are important environmental factors that greatly limit cotton production in the world [9]. Plant adaptation to abiotic stresses is dependent upon the activation and trigger of various molecular networks, which are involved in stress perception, signal transduction, metabolites and the induction of specific stress-related genes [10]. Stress-inducing factors can occur simultaneously or sequentially and cause osmotic stress, water deficits, ionic imbalances, peroxidation damage, ultimately, growth inhibition [11]. Calcium plays a major role in abiotic stress response as the second messenger [12, 13]. Salt and cold forms of stresses could enhance cytosolic free calcium concentration in plants. Research has shown that OSCA1, reduced hyperosmolality-induced calcium increase 1, is a putative sensor for osmotic stress, involving in cold and salt stress response [14–16]. MAPK (Mitogen-activated protein kinase) cascades, stimulated by the second messenger, for example, calcium, participate in abiotic stress signal transduction [16]. Moreover, SnRK2 (Sucrose non-fermenting-1-related protein kinase 2) family of protein kinases is also involved in signal transduction under salt, osmotic and drought stress treatments. MAPK and SnRK2 could be rapidly activated by cold and salt in plants [15, 16].

An increasing number of genome sequencing and resequencing, mRNA sequencing and phenotypical assesses of cotton [15] provides important resources for studying potential biological mechanisms in cotton. Comparative transcriptome analysis usually used to construct a regulated model by gene expression changes [17]. Phylotranscriptomic analysis provides a new strategy to investigate the gene evolution and expression change during domestication [14]. For example, hundreds of candidate genes that have evolved new protein sequences or have changed expression levels in response to natural selection were identified in wild tomato relatives by the phylotranscriptomic analysis, indicating artificial and natural selection have had on the transcriptomes of tomato and its wild relatives and expression change play an important role in the evolution and domestication [18]. The weighted correlation network analysis (WGCNA) is an R package for gene co-expression network (GCN) analysis and can be used as a data exploratory tool or a gene screening (ranking) method to find clusters (modules) of highly correlated genes [19]. It was used widely to find hub genes in biomedical science [20].

In our research, to reveal the genetic and expression diversity under cold and salt stress, we perform transcriptomic sequencing of four diploid D-genome species, including *G. thurberi*, *G. klotzschianum, G. raimondii* and *G. trilobum*. The phylogenetic relationship of four species was in line with previous studies [3]. Six species-specific profiles and four species-specific modules were identified by comparative transcriptomics. In the expression of various genes, more genes exhibited differential expression under cold in contrast to salt stress. The gene evolutionary analysis identified hundreds of PSGs in different genomes or subgenomes (wild species: *G. thurberi*, *G. klotzschianum, G. raimondii,* and *G. trilobum*; cultivated species: *G. arboreum*, *G. hirsutum* A_t_ and D_t_, *G. barbadense* A_t_ and D_t_). We also found a module was negatively correlated with salt and cold stress by WGCNA. *G. klotzschianum* showed better resistance under cold and salt stress, and 171 common DEGs under cold and salt stress were identified in this species. In summary, gene expression variations were essential drivers of the morphological variations related to environmental adaptation during evolution and there are shared networks that involved in cold and salt stress response, such as signal transduction and oxidation-reduction processes. Our work provides an insightful understanding of expression divergence, conservation, and response to environmental adaptation during evolution by combining protein-coding sequence and gene expression diversity.

## Result

### Phenotyping diversity and RNA-seq data of diploid D genome species

**Fig. 1:**
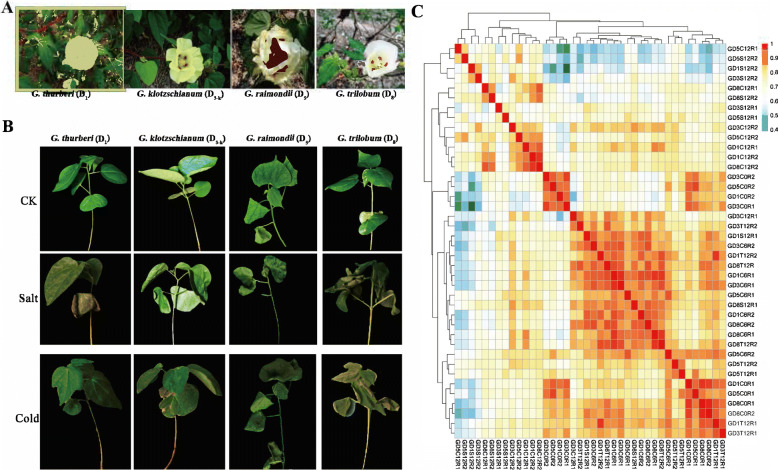
Phenotypic variations and correlation of whole-genome expression of four species. **a** Phenotypic variations in flower color and leaf shape. **b** Tolerance divergence of four species in seedlings. **c** Heatmap of correlation value (R square) of 40 libraries

To observe an evolutionary divergence and conservation at the transcriptomic level of wild diploid D-genome cotton, four cotton species including *G. thurberi* (D_1_), *G. klotzschianum* (D_3-k_)*, G. raimondii* (D_5_) and *G. trilobum* (D_8_) with a rich diversity of morphological characteristics were selected for further analysis. These species show tremendous phenotypic variations in flower color and leaf shape, although these are phylogenetically most closely related to each other (Fig. 1a). Additionally, four species presented variant cold and salt resistance: *G. klotzschianum* and *G. thurberi* showed excellent resistance to cold and salt stress, followed by *G. raimondii* (Fig. 1b). Strangely, *G. trilobum* indicated the lowest resistance, despite it is most closely related to *G. thurberi*. For a comprehensive evaluation, we carried out the RNA-sequencing of D-genome diploid cotton. Four species, *G. thurberi*, *G. klotzschianum, G. raimondii,* and *G. trilobum*, were abbreviated as GD1, GD3, GD5 and GD8 for convenience. Ten leaf samples were collected from each species in different time intervals (0 h, 6 h, and 12 h after a three-leaf stage of seedlings: C0, C6, and C12) after two stress treatment samples (Cold T12 and salt stress S12), two repeats for each sample. A total of 40 data sets with 273.01 GB raw data were obtained. The resulting GC content rates of 44.41–46.64% are similar in a different dataset. A total of 273.01 Gb clean data without an adapter, ploy-N, and lower quality reads were obtained after quality control. Every dataset is at least 17.1 million clean reads and more than 89.03% of base Q30. After that, the RNA-sequenced clean reads were then anchored onto the reference genome, *G. raimondii*. The mapped clean read ranged from 81.37 to 91.37% of the clean reads were uniquely mapped to the reference genome (Table S1). Based on mapping results more than, 7374 SNPs (Single Nucleotide Polymorphisms) were identified in each library (Table S2). The expression level of 41,053 genes, including 3548 new transcriptional genes, is quantitated. Except for samples of GD1S12, the R1 and R2 libraries in the same sample area with a high value of correlation (*R*^*2*^ *> 0.7*); suggested datasets of other samples are reliable (Fig. 1c). The reason for the lower value of the correlation between GD1S12R1 and GD1S12R2 may be the effects of developmental and environmental variation on gene expression, although we try to minimize it.

### Genetic divergence of four diploid D genome species

**Fig. 2:**
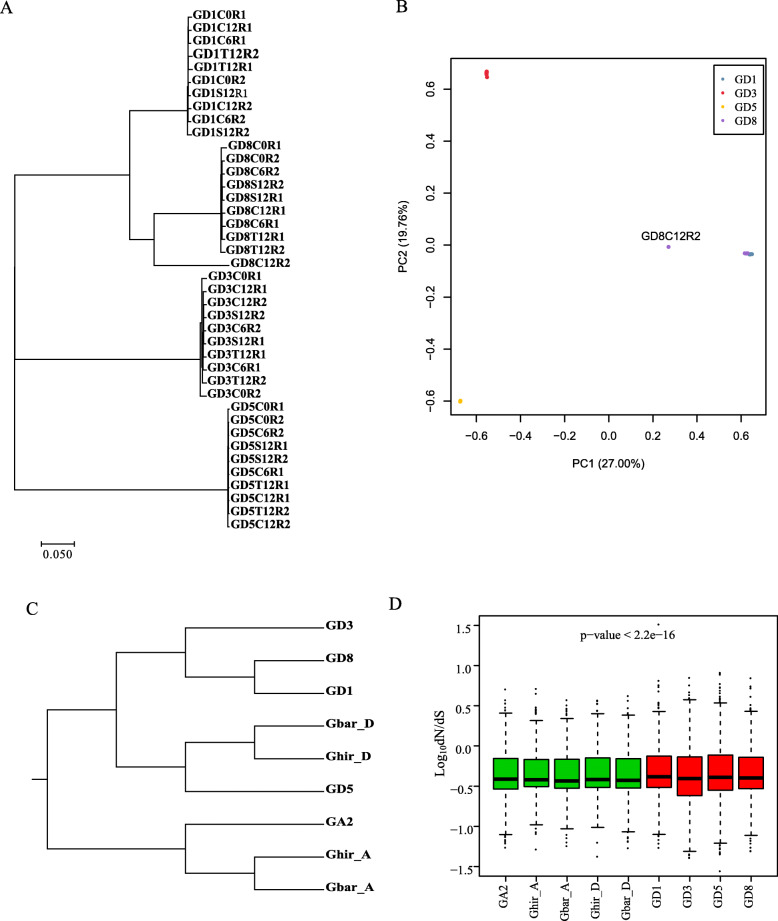
Sequence diversity of four species of cotton. **a** Unrooted phylogenetic tree of four species using SNPs, which obtained from transcriptomic data. The scale bar represents the expected number of substitutions per site. **b** The relationship showed by the principal component cluster among four species. **c** Phylogenetic tree of nine genomes using the identified orthologous genes. The scale bar represents the expected number of substitutions per site. **d** Boxplot of the dN/dS ratio of nine genomes. Wild species, red boxes. Cultivated species, green boxes

The transcript sequence polymorphism analysis contributed to understanding the evolutionary diversity and conservation at the transcriptomic level in four D-genome diploid cotton. The RNA-Seq data were used for sequence polymorphism discovery and 7374–404,737 SNPs were identified in different libraries (Table S2). More than 59% of SNPs have a transition type. On account of the datasets was obtained by transcriptomic sequencing, the chromosomal distribution of more than SNPs in introgenic region. Of note is the observation that about 9% SNPs were identified in the intergenic regions of GD1, GD3, and GD8, representing a new genic region in other D-genome species. Libraries of GD5 (*G. raimondii*) have a fewer number of SNPs as compared to the other three species. In general, transcriptomes of GD5 samples should have the same genome with a reference genome. Although the transcriptome of GD5 samples and reference genome was very similar to each other (< 1 SNP per Kb of most genes), some SNPs were observed between GD5 and the reference genome, suggesting that there existed in differences in the leave′s transcriptome among plants of the same donor material. A total, 3, 2651 genes were expected to have high functional effects by SNPs in four diploid D-genome kinds of cotton. Go ontology (GO) enrichment analysis showed that a high number of the genes were highly involved in various processes such as the oxidation-reduction, stress response, protein modification, and protein phosphorylation. It has been demonstrated that abiotic stresses have played an important role in driving the transcriptional diversity among the four cotton species (Table S3). On the contrary, it is also noticeable that 8402 genes have no SNPs function-effect, including many housekeeping genes such as (Tubulin, ribosomal protein, and glycolytic enzyme-coding genes). A total of 8402 genes were involved in several biological pathways including photosynthetic electron transport chain. We used Neighbor-joining methods to construct a phylogeny tree of four species base on transcriptomic data (Fig. 2a). And, the principal component cluster was performed to observe the relationship between four species (Fig. 2b). A modest number of SNPs separate them among four species (< 5 SNPs per kb of most of the genes). The datasets of the same species almost overlap with each other. *G. thurberi* showed closer relationships with *G. trilobum* has previously been demonstrated.

### Transcriptome De novo assembly

Transcriptome De novo assemblies were performed based on RNA-seq data using Trinity software. Ranged from 47,180 to 55,548 unique genes were identified in four diploid species. Most of the unigenes (*> 40%*) lengths were ranged from 200 to 500 bp. To initiate our evolutionary analysis, we identified the strictly orthologous unigenes among 7 species, including three cultivated (A_2_-genome: *G. arboreum*; AD_1_-genome: *G. hirsutum*; AD_2_-genome: *G. barbadense*) and four wild species. For rigorous analysis, the genomes of allotetraploid species were separate A and D subgenome to identify orthologous transcripts. A total of 47,119 orthologous gene pairs were characterized, and 5312 single-copy transcript pairs of those were used to construct the phylogenic tree of the nine genomes by maximum likelihood methods. Phylogenetic analysis using these unigenes revealed a quite similar topology (Fig. 2c) of the tree based on transcriptomic SNP data (Fig. 2a), indicating a solid phylogeny for four D-genome diploid species. Same with the previous study, A subgenome of *G. hirsutum* and *G. barbadense* originate from *G. arboreum*, which formed monophyly in our phylogenetic analysis. *G. hirsutum*-D subgenome, *G. barbadense*-D subgenome, and GD5 formed monophyly. These results proved D subgenome of allotetraploid AD_1_ and AD_2_ genomes have the same donors, originated from *G. raimondii*.

### Estimate evolutionary rates and identify positively selected genes (PSG)

Non-synonymous (dN) and synonymous (dS) are used to estimate the evolutionary rate and positively selected genes (PSGs) in each species. If dN/dS value is *> 1*, this is indicative of positive selection, whereas equal to or greater than 1 (> 1) denotes either purifying or neutral selection. Therefore, we evaluated the dN, dS, and dN/dS of the identified orthologous single-copy genes among nine genomes. Based on the above-constructed phylogenetic tree, the dN/dS for each orthologous unigene pair was evaluated in the different branches using a free ratio model (model = 1), which allows for a separate dN/dS ratio for each branch. We found that wild diploid cotton (GD1, GD3, GD5, GD5_ref, and GD8) had higher dN/dS ratios than the branch of cultivated cotton (GAD1, GAD2, and GA2). Under natural selected pressure, wild species are showed a fast evolutionary ratio based on adaptive choices (Fig. 2d). A total of 163, 344, 330, and 161 PSGs in GD1, GD3, GD5, and GD8 were identified by PAML software, respectively (Table S4). PSGs of diploid kinds of cotton were enriched in GO terms related to protein modification, protein ubiquitination, RNA processing, and ncRNA processing, involved in environmental adaptation (Table S5). What is more, we also found 180, 77, 51, 103, and 70 PSGs in GA2, GAD1_A, GAD1_D, GAD2_A, and GAD2_D, and these PSGs were enriched mRNA metabolic process (Table S6).

### Gene expression divergence and conservation

**Fig. 3:**
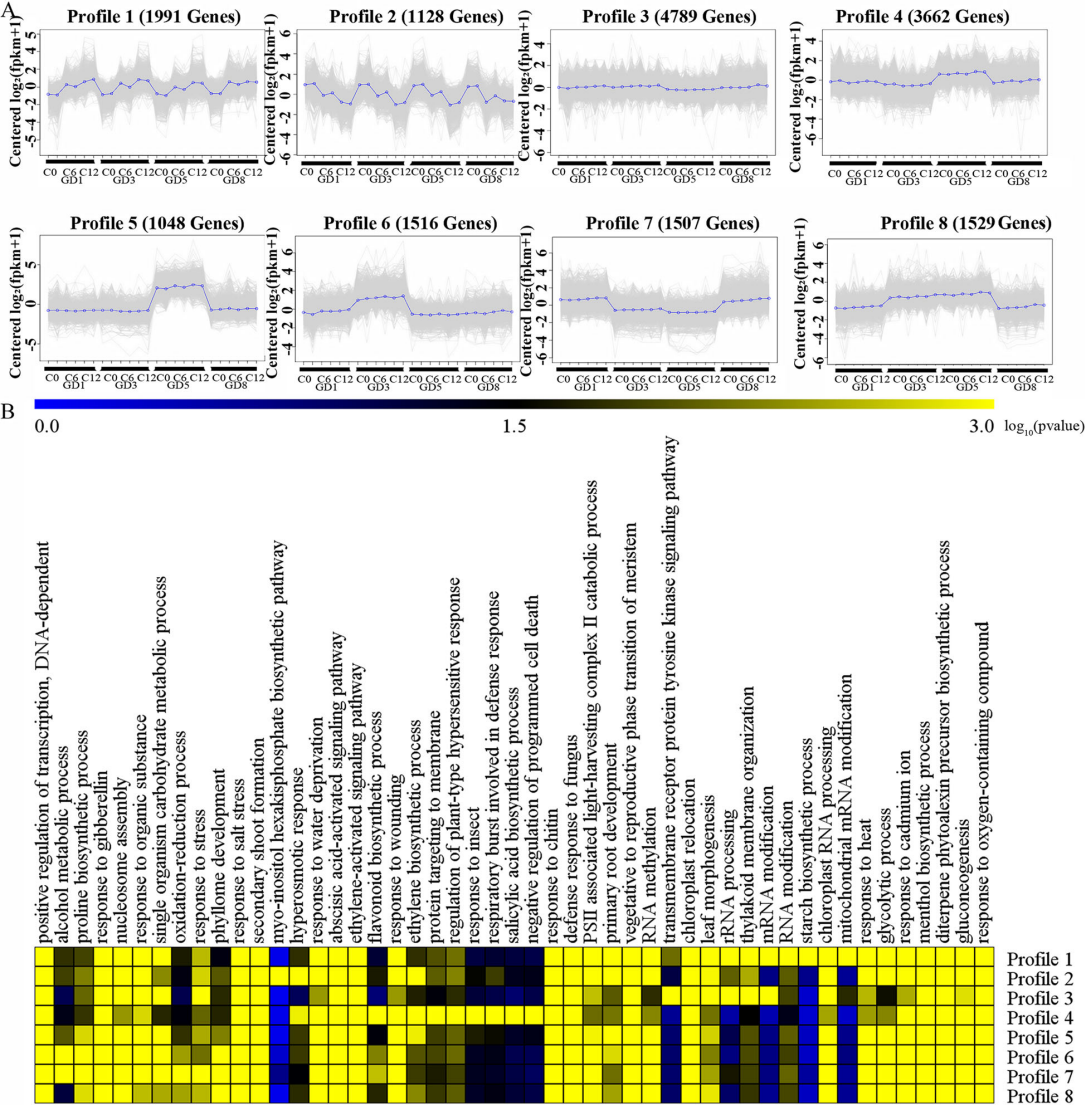
Gene expression pattern across three-time points (C0, C6, and C12) of four species under normal condition and corresponding top fifteen enrichment GO terms. **a** Gene expression pattern across three-time points. Eight gene clusters (profile 1–8) were identified using k-means clustering. In each cluster, the y-axis represents log_2_ (FPKM+ 1) derived from RNA-seq data for each biological sample, while the x-axis represents the biological samples that are ordered as C0 (R1 and R2), C6 (R1 and R2), and C12 (R1 and R2) for each species. **b** Heatmap of –log_10_ (*p*-value) of biological process category enrichment among the eight profiles

We detected expression levels of a total of 41,053 transcripts in at least a single sample. Global expression level distributions of 40 data sets were similar to each other. Twenty-four datasets of leaves without stress treatment were used to detect gene expression divergence and conservation of four species. Samples of GD5C0R1 and GD5C0R2 were as a control group to identify different expression transcripts. Patterns of differential gene expression were characterized by four species (Fig. 3a). A total of 29,512 DEGs was clustered eight profiles based on gene expression pattern use k-means clustering method. Profiles with a large number of DEGs, display a similar expression pattern (Profile 1, 2 and 3) of four species. The profiles 1 and 2 showed considerable differences in expression over time in the leaves of four species. In the Profile 1, the DEGs were down-regulated expression level over time and enriched in carbon metabolism and carbon fixation in the photosynthetic organism. On the contrary, DEGs of the profile 2 were up-regulated and enriched in plant circadian rhythm and ribosome biogenesis in eukaryotes. In the profile 3, DEGs were significantly enriched biosynthesis of unsaturated fatty acids, DNA replication, and photosynthesis-antenna proteins process (Fig. 3b). Those pathways are indispensable during leaf’s growth and development, while transcripts expression of those pathways is conservation during the divergence of D-genome species of cotton. Profile 4–9 was observed a considerable species-specific expression pattern. In the profile 4 and 5, 3662 and 1048 DEGs displayed GD5-specific expression pattern. What′s more, in the profile 6, 1516 DEGs displayed GD1-specific expression pattern. And, in profile 7 and 8, showed GD3-GD5 and GD1-GD8 special expression pattern. Given GD1 and GD8 with a close relationship, it made perfect sense that GD1 and GD8 contain more conservative biological pathways.

### Evolutionary conservation and divergence of the gene co-expression networks

**Fig. 4:**
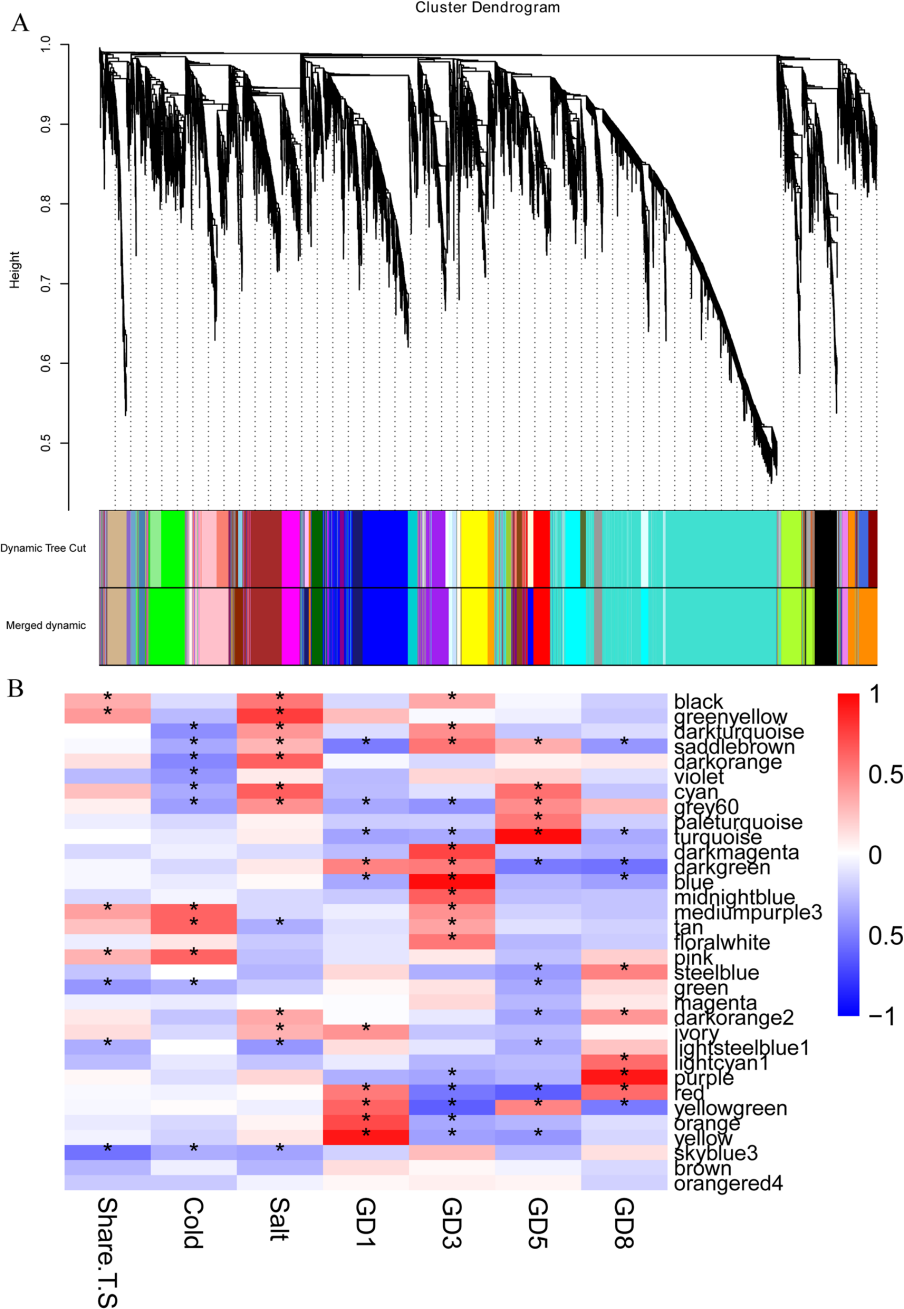
Co-expression network analyses by WGCNA. **a** Hierarchical cluster tree showing co-expression modules identified by WGCNA. Each leaf in the tree represents one gene. The major tree branches constitute 33 modules labeled with different colors. **b** Module–sample association. Each row corresponds to a module labeled with color as in (A) Modules are distinguished by different colors which were arbitrarily assigned by the WGCNA package. Each column corresponds to a tissue type as indicated. The color of each cell at the row-column intersection indicates the correlation coefficient (R) between the module and the tissue type. *Significance at *P < 0.05*; **Significance at *P < 0.01*

Notwithstanding the analysis of gene expression pattern provided insights of gene expression divergence and conservation, co-expression gene networks, constructed by highly connected genes, and is more relevant to the vital biological processes of growth and development, and complex regulatory process of abiotic stress response. To grasp gene co-expression network conservation and divergence, all 40 datasets of leaves were used to construct a co-expression network by weighted gene co-expression network analysis (WGCNA). To enhance the accuracy of the WGCNA analysis, genes with fragments per kilobase of exon model per million reads mapped of less than one (*FPKM < 1)* were removed. In conjunction with connection strengths (soft-threshold power: 5, *R*^*2*^ *> 0.90*) among 12,110 genes (Fig. S1), a global view of co-expression network topology among four species was constructed. Genes in the same showed module showed a higher topological overlap (Fig. S2). Finally, a total of 33 modules were used for investigating evolutionary conservation and divergence of gene co-expression networks, which are defined as clusters of highly interconnected genes (Fig. 4a). In these modules, the number of the genes ranged from 31 (dark orange) to 3128 (turquoise) with high correlation coefficients with one another in corresponding modules. Every gene connectivity was evaluated on each module. Highly connected genes (*kME > 0.95*) were identified as hub genes. Ultimately, a total of 425 hub genes (ranging from 1 to 293 within the modules) were detected.

In each module, expression levels of all genes were displayed by a heatmap and were summarized by the eigengene values (the first principle component of module expression profiles). Seven sample conditions were defined for identified significant modules (Fig. 4b). Share.T.S group was used for identifying conservative shared networks of the cold and salt stress response. Additionally, the module, correlated with cold and salt stress response, was identified by Cold and Salt Groups, respectively. GD1, GD3, GD5, and GD8 Groups were used for identifying genome-specific modules. By association analysis between eigengenes and sample conditions (7 groups: Share.T.S, Cold, Salt, and 4 genomes) via Pearson correlation coefficient analysis, 29 modules were identified with significant genome-specific and/or abiotic stress-regulated co-expression patterns (ANOVA, *P < 0.05*). Four major modules of highly co-expression genes were most strongly correlated (Pearson’s correlation *r > 0.9*) with four genomes, respectively. The largest module (turquoise), containing 3128 highly connected genes in GD5, enriched in response to stimulus and immune system process (Table S7). A second module (Blue, 1193 genes), showed GD3 specific. The other two modules (yellow with 420 genes and purple with 442 genes), which included few highly connected genes, respectively related to GD1 and GD8. Interestingly, all four major modules displayed similar results of GO enrichment analysis. Almost a half of the genes enriched for GO terms were related to response to stimulus and immune system process in each module, revealing that abiotic and biotic stresses have played a major role driving transcriptional variation among these four species. We noticed that characteristics of the seven modules were unique related to the corresponding group, and among these modules, five modules were genome-specific. What′s more, most of the modules most strongly correlated with four genome group. These results suggested transcriptional variation is mostly correlated with genome divergence. We observed some modules overlapped among different groups. Overlap modules are helpful to understand the similar biological characteristics among two or more genomes. For example, the dark green module was significantly showed a positive correlation with GD1 and GD3, but a negative correlation with GD5 and GD8, and enriched sesquiterpenoid and triterpenoid biosynthesis and flavonoid biosynthesis which were known related with abiotic and biotic stress resistance.

Of particular concern is that co-expression networks related to cold and salt stress, due to four diploid species presented different cold and salt stress resistance. Association analyses between co-expression modules and abiotic stress (Cold and salt stress) revealed that 17 modules correlated with abiotic stress (Shar.T.S: 7 modules; Cold: 8 modules; Salt: 8 modules), thus representing suites of interconnected genes underlying the biological process of the abiotic stress response (Table S7). Noticeably, among four species, GD3 and Salt/Cold group had more overlapped modules. Considering GD3 showed better cold and salt tolerance than the other three species, suggested GD3 evolved more complete mechanisms in abiotic stress adaptation. On the contrary, almost no one module showed overlap between GD8 and abiotic stress groups, and it is foreseeable that GD8 manifests as significantly sensitive under cold and salt.

**Fig. 5:**
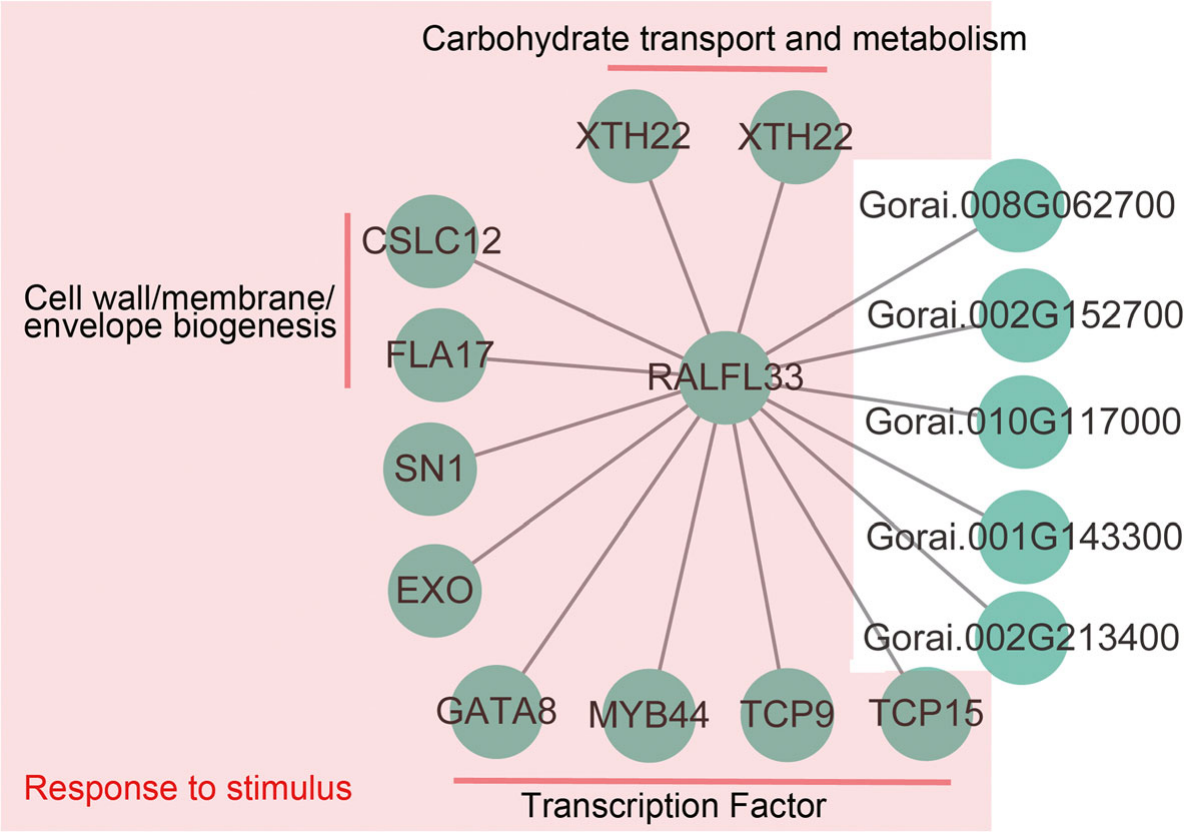
Co-expression network of hub gene in skyblue3 modules. Genes were related to responses to stimuli in a red background. Five function unknown genes were displayed in white background

Interestingly, the skyblue3 module exhibited a significantly negative correlation with the Share.T.S, Cold, and Salt groups. One of the *hub* gene, RALF (rapid alkalization factor)-like was identified in within the module. The homolog gene from the Arabidopsis perhaps could be responsible for the regulation of the plant stress response and or adaptation, growth, and development. Fifteen genes in this module were interconnected with the RALF-like protein coding gene. Except for five uncharacterized genes, ten out of fifteen interconnected genes were associated with the GO term, response to the stimulus, including *Gorai.005G234900*, *Gorai.007G094200*, *Gorai.N023400*, *and Gorai.011G238800*. The homologs from Arabidopsis have been annotated for four important transcription factors: MYB44, TCP9, TCP12, and GATA8, and are majorly involved in abiotic stress response. Moreover, two xyloglucan endotransglucosylase/hydrolase protein 22 (XTH22)-encoding genes, *Gorai.003G052400* and *Gorai.009G006400*, were observed to be involved in carbohydrate transport and metabolism. Homologs of *Gorai.005G094300* in Arabidopsis, EXORDIUM protein-encoding gene, required for cell expansion in leaves, and may be involved in signaling processes that coordinate brassinosteroid (BR) responses to environmental or developmental signals (Fig. 5). Most of the 15 genes were decreased the expression level after cold and salt stress treatment.

### Characterization of DEGs under cold and salt stress and validation of key genes under salt-alkali stress conditions

**Fig. 6:**
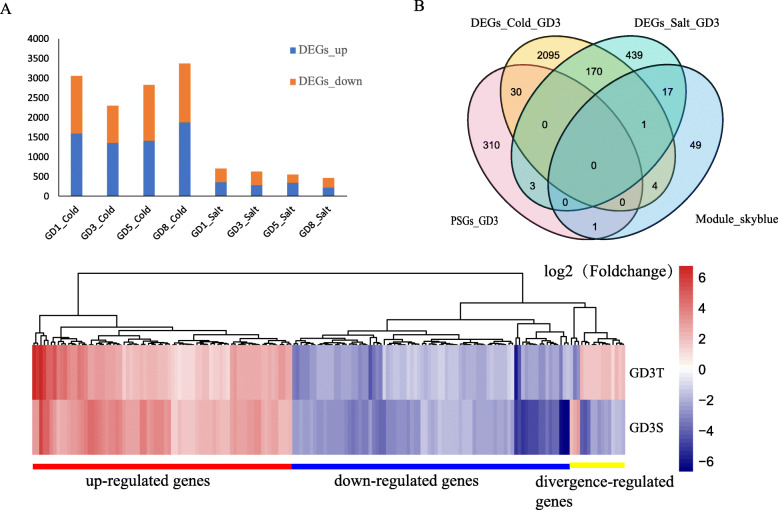
Characterization of DEGs under cold and salt stress. **a** Numbers of DEGs of four species under cold and salt. Red block means numbers of down-regulated genes. Blue block means numbers of up-regulated genes. **b** DEGs of GD3 under cold and salt stress; PSGs of GD3; and genes of skyblue3 module showed by Venn. **c** Common DEGs of GD3 under cold and salt stress

The false discovery rate (FDR) *≤ 0.001* and log_2_ rates *≥2* (8 groups: treatment/control) were used to identify DEGs in four species. The number of DEGs varied from 459 to 3372 among treatments of 8 groups (Fig. S3). A total of 7515 DEGs were found, occupying 20.04% of total detected genes. Interestingly, the number of DEGs among salt stress groups was always smaller than those among cold stress group in four species examined (Fig. 6a), indicated that the cold stress response was divergent than salt stress response, and more unique DEGs (6405, 85.2% of all DEGs) in one type of abiotic stress again proved that. But, 1109 genes (16.1% of cold DEGs; 63.3% of Salt DEGs) were found in two different abiotic stresses, indicating potential share regulated pathways in cold and salt stress response. As expected, 1109 genes were enriched in the oxidation-reduction pathway, which was involved in multiple abiotic stress responses.

**Fig. 7:**
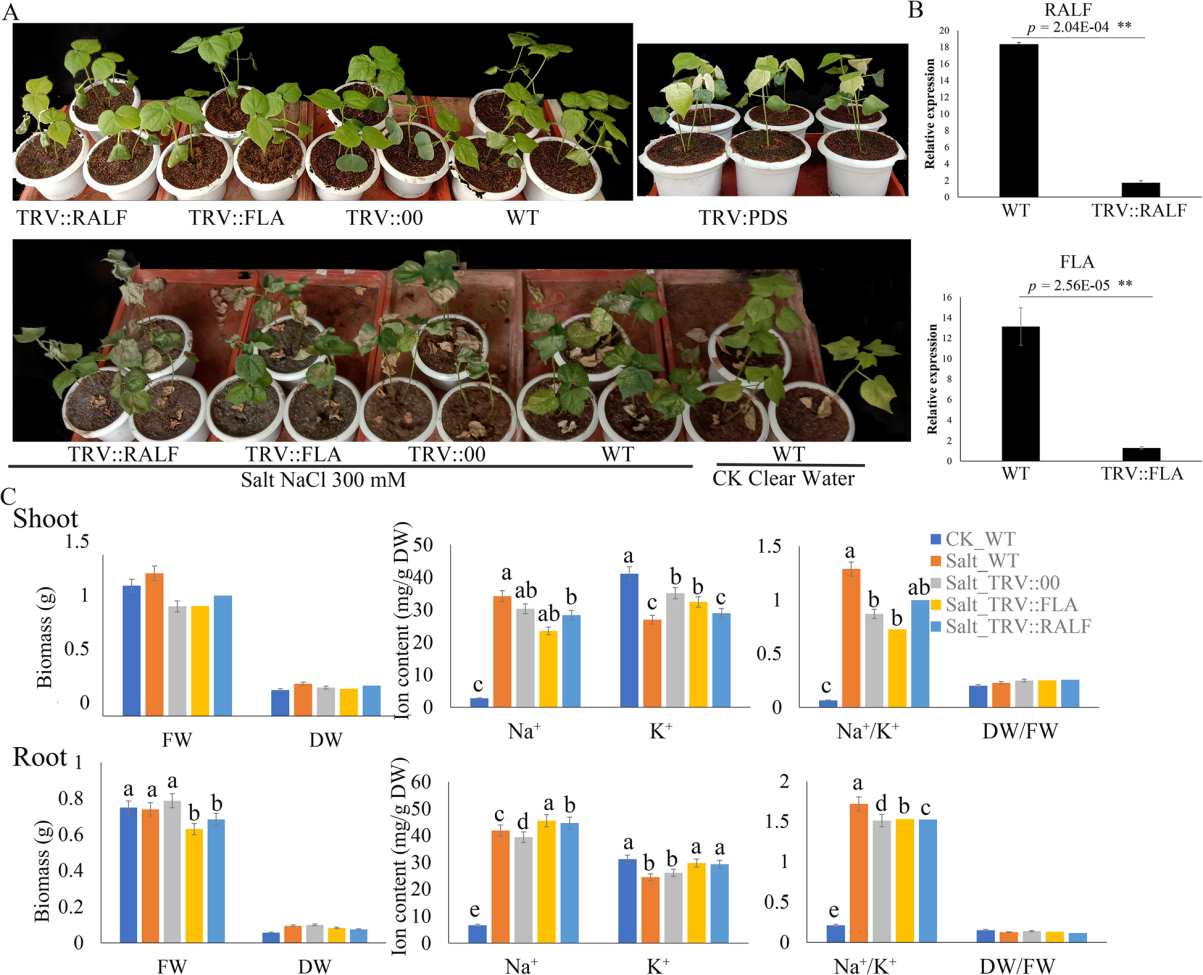
Phenotype observed in the silenced plants with the TRV: 00 empty vectors, wild type plants, and the silenced plants at 12 days post-inoculation. **a** Albino’s appearance on the leaves of the PDS infused plants. **b** RT-qPCR analysis of the change in the expression level of the *RALF* and *FLA* genes in cotton plants treated with VIGS. **c** Evaluation of fresh shoot biomass, fresh root biomass, Na^+,^ and K^+^ ions concentration and their ratios. Letters a/b indicate statistically significant differences (two-tailed, *p < 0.05*). The error bars of the *RAL*F and *FLA* gene expression level represent the standard deviation of three biological replicates

We thus focused on DEGs in GD3, on the account showed higher tolerance under cold and salt stress, and ultimately identified 2759 DEGs in salt and cold groups of GD3, including 2300 genes cold DEGs, 630 salt DEGs. Among those genes, we found171 share DEGs under salt and cold stress, containing 80 down-regulated and 75 up-regulated DEGs (Fig. 6c). Interestingly, 102 genes of share DEGs involved response to stimulate, and 32 genes of those genes were related to response to cold or salt stress, including 13 transcriptional factors encoding genes (NAC, ERF, MYB, G2, HD-ZIP) are putatively related to response to abiotic stress, since some homologs of those genes in Arabidopsis related to the abiotic stress response. For example, *Gorai.002G073700* encodes the homolog of Arabidopsis NAC72, which binds to a drought-responsive *cis-*element in the early responsive to dehydration stress 1 promoter [21]. Also, both *Gorai.001G239000* and *Gorai.006G017400* encode homologs of MYB-like protein in Arabidopsis that involve in plant defense response [22]. Notable a total of 30 cold DEGs and three salt DEGs were PSGs. Moreover, three cold DEGs and 12 salt DEGs were in the skyblue3 module. Although fewer DEGs (Cold DEGs: 6.1%; Salt DEGs: 1.7%) were identified above all salt DEGs, some DEGs were overlapping with the PSGs or skyblue3 module (Fig. 6b). Those results indicate again adaptive evolution drives transcriptional diversity, and a share regulated network involved in cold and salt stress response. We also found *Gorai.006G147500* of skyblue3 module genes is positively selected and is interconnected with the *hub* gene, RALF-like protein coding gene. It further confirmed the potential regulated network that was identified in the previous analysis. Validation of the key genes through reverse genetics revealed that the VIGS-plant’s ability to adapt to the salt-alkali was highly compromised. The VIGS-plants exhibited wilting and gradual drying of the leaf margin upon exposure to salt stress (Fig. 7a), the results were in agreement with previous findings which demonstrated that salt stress does negatively affects the leaves and in turn, leads to a significant reduction in above-ground biomass [23].. Furthermore, all exhibited significantly low biomass (Fig. 7b), moreover, determination of ion content of the various tissues, the plants in which the genes was knocked out registered a significantly lower concentration of Na^+^/K^+^ ions, an indication that the balance between the two ions is affected due to salt-alkali toxicity (Fig. 7c).

## Discussion

In a previous study, some researches focus on the relationship of D-genome diploid cotton, for instance, the relationship of *G. trilobum* is close with *G. thurberi,* rather far with *G. klotzschianum* and *G. raimondii* [3]. Range from 7374 to 404,737 million SNPs was detected by aligning our transcriptomic data with the reference genome (*G. raimondii*, JGI). And then, those SNPs were applied for the construction of a phylogenetic tree to investigate the relationship of four species. The relationship of the four species is in line with previous studies, and the result further confirmed feasible genomic sequences analysis based on RNA-Seq de novo assembly. Allotetraploid cotton contains seven species. Based on the similar distribution of 45S and 5S rDNA between allotetraploid and diploid species in chromosomes, some researchers thought different allotetraploid species of the cotton genus have different donor species, such as *G. thurberi*, *G. klotzschianum, G. raimondii* and *G. trilobum* [24]. But, *G. raimondii* showed most synteny blocks with D-subgenome of *G. hirsutum* and *G. barbadense* by comparative genomic analysis, suggested *G. raimondii* is the possible donor species of *G. hirsutum* and *G. barbadense*. So, some researchers thought allotetraploid cotton may be monophyletic. In our study, 5312 single-copy genes were used to construct a phylogenic tree and estimate the evolutionary rate. A-genome and A-subgenomes formed monophyly, and D-genomes and D-subgenomes formed monophyly. Our result support that *G. raimondii* is donor species of D subgenome of *G. hirsutum* and *G. barbadense*. It is consistent with monophyletic evolutionary theory of allotetraploid cotton [7].

Positive selection plays a significant role in the evolution and adaptation of the plants to biotic and abiotic stresses, as gene expression and regulation changes by positive selection have been postulated to be key determinants of the rates of adaptive evolution [25]. Our result manifested wild cotton possessed higher dN/dS ratio (genome-wide accelerated evolution) rather than cultivated cotton, suggesting that wild cotton may have undergone adaptive evolution that allows them to cope with their extremely wide range of terrible conditions and environments. Our study found a hundred genes were positively selected during evolutionary, and those genes also were enriched GO terms related to the abiotic or biotic stress response. And analyses confirmed that positive selection drives the environmental adaptive evolution of wild species. K-means cluster analysis of wild species found that species-special profiles were enriched GO terms related to abiotic stress response, indicated Expression analysis of four wild species also proved that abiotic stresses drive transcriptional diversity among four species [26].

We observed morphological differences between the four species. The different ecosystem of four species leads to the divergence of their morphology. Previous genetic pieces of evidence indicated gene expression alteration is essential to drive phenotypic diversity during evolution [27]. Our results agreed with this conclusion, five species-specific gene expression clusters were identified (profile 4–8), and most of the genes are an enrichment to the GO terms related to environmental adaptation in those five profiles. Similarly, most PSGs of wild species were related to environmental adaption. More than 9.6% of PSGs were overlapped with genes of corresponding DEG sets. Therefore, we speculate evolutionary selection also could drive gene expression alteration to adapt to the environment.

The morphological analysis found that *G. klotzschianum* is tolerant to cold and salt stress. *G. klotzschianum*, *the* natural range is the Galapagos Islands, can adjust itself to the environment. Therefore, we investigated genes in *G. klotzschianum* under positive selection. The numbers of PSGs are low in *G. klotzschianum* (344, ~ 0.9% of all genes in the genome). Nonetheless, 33 PSGs exhibited differential expression under salt and cold stress, indicating that gene expression alteration caused by natural selection might have played an essential role to improve the environmental adaptation.

Combining the analysis of the preceding context that *G. klotzschianum* showed astonishing tolerance under cold and salt stress, we speculate a shared network that involved in cold and salt stress response was formed during the adaptive evolution of *G. klotzschianum*. Thirty-three (~ 10.6%) PSGs were differently expressed under cold or salt stress. In particular, in WGCNA, we found a module that was negatively correlated with salt, cold, and share.T.S conditions. 74% of genes in this module were enriched GO terms related to response to stimuli and 36.3% genes were enrichment GO term related to the signaling process. This result further confirmed our speculation. Simultaneously, we found the stress signaling process is a tentative share regulated network of salt and cold stress response. One hub gene in skyblue3 was found in WGCNA. And, 15 genes with significant correlation to the hub gene were identified. Interesting, *Gorai.006G147500* among 15 high connective genes, were found. These 16 genes of the skyblue3 module were potentially regulated networks under cold and salt stress. The researcher presumes that plant cells must be capable of sensing various environmental signals [28], and some putative sensors were identified in previous studies, such as OSCA (reduced hyperosmolality-induced calcium increase 1) [26], G protein [26], and COLD1 [29]. Salt and cold stress could cause increases in the cytosolic free calcium concentration in plants [30]. The homolog of *Gorai.010G168400* in Arabidopsis, encoding *AtOSCA3.1* protein, play a role in the hyperosmolarity-gated non-selective cation channel that permeates Ca^2+^ ions [31]. And *Gorai.010G168400*, showed a higher expression level under salt and cold stress compared to control groups, was a potential sensor that mediates cold and salt stress in cotton, and calcium ion acts as the second messengers in response to abiotic stress [32]. *Gorai.009G294400*, encoding CBL-interacting protein kinase 18, which involved in Ca^2+^ signal transduction, was also found that different express under cold and salt stress. Core stress-signaling pathways involve protein kinases related to the yeast SNF1 and mammalian AMPK [33]. SNF1/AMPK-related kinases mediate the signaling of various abiotic stresses. Among 171 DEGs of salt and cold group, *Gorai.005G081200* and *Gorai.002G103300* which share homology to SNF1/AMPK in their kinase domains.

The oxidation-reduction process is also involved in salt and cold stress response by keeping the homeostasis of reactive oxygen species [34]. We found that oxidoreductase activity-related genes such as *Gorai.004G093200*, *Gorai.013G025400*, *Gorai.013G059500* and *Gorai.013G176900* were up-regulated under cold and salt stress, correlating with the oxidation-reduction process. PSGs and sky blue-module genes also generated many important candidate genes, such as *Gorai.004G227900* and *Gorai.002G226600*, which are related to keeping the homeostasis of reactive oxygen species. Extensive investigations of these genes under cold and salt stress would help us understand how DEGs regulate homeostasis of reactive oxygen species and signal transduction and their function during cold and salt response, thus helping us to increase yields in cotton.

## Conclusion

Comparative transcriptome analysis of four diploid D-genome kinds of cotton reveals sequence and gene expression variations. Gene evolution analysis of wild and cultivated kinds of cotton identifies positively selected genes which involve in the domestication and evolution of cotton and estimate the evolutionary rates. In this work, we found that evolutionary selection could drive gene expression alteration to adapt environment and gene expression variation is the primary evolutionary event during the divergence of four D-genome species. The expression pattern analysis found that six profiles showed distinct species-specific characteristics and genes of those profiles were involved in response to stimulation. Thus, gene expression variations were essential drivers of the morphological variations related to environmental adaptation during evolution. More DEGs were identified under cold stress in contrast to DEGs under salt stress, indicated cold stress lead to expression change of more genes. *G.* showed *klotzschianum* showed better resistance under cold and salt stress. Compared with the other three species, more PSGs were detected in *G. klotzschianum*, and 9.6% PSGs were differently expressed under cold or salt stress. In *G. klotzschianum*, 27.1% DEGs under salt stress were overlapping with that under cold stress and we found the skyblue3 module was significantly negatively correlated with cold, salt, and share.T.S condition groups. Thus, there are share networks involved in cold and salt stress response, such as signal transduction and oxidation-reduction processes. Based on our multiple analyses, a set of candidate genes involved in cold and salt stress response is putatively proposed, providing genetic resources for multi-abiotic resistant cotton breeding.

## Methods

### Plant growth and sample collection

Four diploid wild species of D-genome cotton were used in this study, including *G. thurberi* (D_1_), *G. klotzschianum* (D_3-k_)*, G. raimondii* (D_5_) and *G. trilobum* (D_8_). There are 13–14 species in diploid D-genome cotton and they are originally distributed from Southwest Mexico to Arizona, with additional disjunct species distributions in Peru and the Galapagos Islands. Here, *G. thurberi*, *G. klotzschianum, G. raimondii* and *G. trilobum* were obtained from USDA-ARS Southern Agricultural Research Centre in College Station, Texas, USA and currently perennially preserved in the National Wild Cotton Nursery, located in Sanya, Hainan province, in the peoples republic of China and managed by the Institute of Cotton Research, Chinese Academy of Agricultural Sciences (ICR-CAAS). The wild cotton species seeds were obtained from the wild cotton nursery which is under the management of the institute of cotton research, Chinese Academy of Agricultural Sciences, (ICR-CAAS), China. The seeds first germinated at 28 °C, 16 h light/8 h dark cycle, and light intensity of 150 μmol m^− 2^ s^− 1^ in 15% water content sands. Three days after germination, the proper plants were potted in soil and placed in a growth room under the same condition. The seedlings were grouped into the three categories, at two simple leaves and one heart-shaped leaf (time point: 0 h) stage. First group put under normal condition (28 °C, 16 h light/8 h dark cycle and light intensity of 150 μmol m^− 2^ s^− 1^ and 15% water content), a second group was watered with 300 mM NaCl solution (28 °C, 16 h light/8 h dark cycle and light intensity of 150 μmol m-2 s-1 and 15% water content) and the third group put at low temperature (4 °C, 16 h light/8 h dark cycle and light intensity of 150 μmol m^− 2^ s^− 1^ and 15% water content). The first group was used to reveal the expression divergence and conservation and leaves of seedling were collected at 0 h, 6 h, and 12 h. Second and third groups were used to reveal regulation under cold and salt stress, and leaves were collected at 12 h. This experiment had two repeats.

### RNA extraction, library construction, and RNA-seq

The RNA from the samples was extracted by use of TRlzol Reagent (Life Technologies, California, USA) in three biological replicates as the instructional manual. RNA quality and concentration was verified by use of Agilent 2100 Bioanalyzer (Agilent Technologies, Inc., Santa Clara, CA, USA). The RNA quality was further checked by NanoDrop 2000 spectrophotometer and electrophoresed on the agarose gel. RNA with quality and purity in the 260/280 ratio of 1.8–2.1, 260/230 ratio ≥ 2.0 were applied for further analysis. The RNA was then transcribed into by use of M-MLV transcriptase kit, obtained from TaKaRa Biotechnology, Dalian, China. Moreover, the mRNAs retrieved by NEBNext Poly (A) mRNA Magnetic Isolation Module (NEB, E7490). The transcribed cDNAs were applied to construct the cDNA libraries using the NEBNext Ultra RNA Library Prep Kit for Illumina (NEB, E7530) and NEBNext Multiplex Oligos for Illumina (NEB, E7500). Briefly, the enriched mRNA was fragmented into RNAs with approximately 200 nt, which were used to synthesize the first-strand cDNA and then the second cDNA. Adaptor ligation procedure was used to analyse the double stranded cDNAs, and the conformed fragments were retrieved by Agencourt AMPure XP beads (Beckman Coulter, Inc.), and enriched by polymerase chain reaction (PCR) amplification. Sequencing of the constructed cDNA libraries was carried out on a flow cell by use of an Illumina HiSeq™ 2500 sequencing platform. Beijing Biomarker Technologies (http://www.biomarker.com.cn) provides experimental procedures and commercially performed it.

### Processing of RNA-seq data

Raw data in fastq format processed through in-house Perl scripts procedure in which the clean data were retrieved by eliminating those reads with ploy-N, adapter and, low quality reads. Moreover, the GC-content, Q20, Q30, and sequence duplication level were evaluated for the retrieved clean reads. All the downstream analyses were based on clean data with high quality. The clean reads were then mapped to the reference genome sequence by use of Hisat2 tool, the perfectly conforming reads were further analysed.

### Gene expression analysis

The evaluation of the quality of the various gene expression profiles, the expression levels were analysed by fragments per kilobase of transcript per million fragments mapped (FPKM) [35]. Differential expression analysis of two conditions/groups was evaluated by using the DESeq2, in which the *P* values modified as per the Benjamini and Hochberg’s approach to regulate the false discovery in which genes with *P*-values of less than 0.01 were designated as differentially expressed genes (DEGs) [36].

### SNP calling

Picard - tools v1.41 and samtools v0.1.18 was employed to sort and remove the duplicated reads and integrate the bam alignment per sample. The Samtools software was employed to carry out the Single nucleotide polymorphisms (SNP) calling. The raw vcffiles were filtered with GATK prescribed standard filter method and other variables. The standard and variables were programmed as clusterWindowSize: 10; *MQ0 > = 4* and (MQ0/ (1.0*DP)) > 0.1; *QUAL < 10*; *QUAL < 30.0* or *QD < 5.0* or *HRun > 5*, and only those SNPs with distance > 5 were used.

### De novo Transcriptome assembly

The left and the right files from all libraries were pooled into one large left and right.fq file. Transcriptome assembly was established based on the left.fq and right.fq by use of Trinity with parameters set with min_kmer_cov set to 2 by default and all other set to default [37]. Expression analysis, SNP calling, and transcriptome assembly was performed using BMKCloud (www.biocloud.net) in this research.

### Ortholog identification, phylogenetic analysis, evolutionary rate estimation and positively selected genes (PSG) identification

High-quality draft genomes of two allotetraploid cotton (*G. hirsutum* and *G. barbadense*) and two diploid kinds of cotton (*G. arboreum* and *G. raimondii*) were obtained from Cottongen database (https://www.cottongen.org). The genome of allotetraploid species contains two subgenomes (A- and D-subgenome). To ensure the accuracy and reliability of ortholog identification, two subgenomes of allotetraploid species were separated. Together with our results of transcriptome assembly (*G. thurberi*, *G. klotzschianum,* and *G. trilobum*), a total of nine genomes were used for phylogenetic analysis and A-genome clades were as out-group (*G. arboreum*, *G. barbadense* A subgenome and *G. hirsutum* A subgenome). Orthofinder [38] used to cluster genes into orthologous gene families with S set diamond and all other parameters set to default. Single-copy orthologous gene pairs with one copy from each genome and subgenome were used for phylogenetic analysis. Protein sequences of single-copy gene families were aligned by MUSCLE (v3.8.1551) [39]. Well-aligned protein sequences were obtained using Gblocks [38, 40]. Based on 4D sites from the coding sequence (CDS) alignments were used to construct a phylogenetic tree by RA x ML [41]. Moreover, a Neighbor-Joining (NJ) tree was built using transcriptome data from four species (*G. thurberi*, *G. klotzschianum*, *G. raimondii,* and *G. trilobum*) SNPs using BMK Cloud (www.biocloud.net). The evolutionary rate of each lineage for the nine gnomes was estimated using the codeml program in the PAML [42] package with a free ration model (*model = 1*). We grabbed the dN, dS, and dN/dS from the result of Codeml. We filter the Genes with dS = 0. If the PSGs *dN/dS > 1* were reported as PSGs.

### Gene clustering and visualization

To determine the expression patterns of the genes over time within each species, K-means clustering was used to visualize genes expression pattern (log_2_-transformed FPKM values) using BMKCloud (www.biocloud.net).

### Weighted gene co-expression network analysis (WGCNA)

The WGCNA [19] package was employed to identify gene co-expression networks and investigate trait-related modules. The WGCNA Was used as described by Xu et al. [43]. The parameter denotes the soft threshold for the correlation matrix, which emphasize denotes a correlations between genes [44]. A value of 5 was selected as the determinant of the soft-thresholding power and the evaluation scale for free topology analysis. The dynamic tree-cutting algorithm and the association of modules with the traits were evaluated as described by Xu et al. [43].

### Validation of the functional analysis of the key genes

The virus induced gene silencing (VIGS) of candidate genes, RALF and FLA, in *G. hirsutum* race Marie-Galante 85 was performed by adopting the laid down procedures [43, 45]. Firstly, we constructed the VIGS vector of TRV: RALF, TRV: FLA and TRV: PDS and introduced into *Agrobacterium tumefaciens* strain GV4104. The Agrobacterium culture was agroinfiltrated into two expanded cotyledons of 10-day-old soil-grown seedling of *G. hirsutum* race Marie Galante 85 (MAR85). The cotton seedlings were planted in the greenhouse with the temperature set at 26 °C, and 16 h light/8 h dark cycle. A total of 24 seedlings were inoculated for each construct. After 14 days of Agrobacterium inoculation, the VIGS-plants, and non-VIGS plants were then exposed to salt-alkali treatment for 6 days. The samples were then collected for morphological and physiological analysis.

### Statistical analysis

All data generated from this experiment was analysed by using the R (v3.5.0) software.

### Data deposition

The RNA-seq data generated in this research work and reported have been deposited in National Center for Biotechnology Information (NCBI) under accession number PRJNA554555 (https://www.ncbi.nlm.nih.gov/sra/ PRJNA554555).

## Supplementary Information

**Additional files 1:** **Table S1.** Summary of RNA-seq data.

**Additional files 2:** **Table S2.** SNPs information of all samples.

**Additional files 3:** **Table S3.** GO enrichment of high functional effects by SNPs in four diploid D-genome kinds of cotton.

**Additional files 4:** **Table S4.** PSGs identified nine genomes.

**Additional files 5:** **Table S5.** GO enrichment analysis of PSGs in wild species.

**Additional files 6:** **Table S6.** GO enrichment analysis of PSGs in cultivated species.

**Additional files 7:** **Table S7.** Gene number of eight import modules in ten GO terms.

**Additional files 8:** **Figure S1.** Determination of soft-thresholding power. (A) Analysis of the scale-free fit index for various soft-thresholding powers (β). (B) Analysis of the mean connectivity for various soft-thresholding powers.

**Additional files 9:** **Figure S2.** Heatmap plot of the topological overlap matrix.

**Additional files 10:** **Figure S3.** Venn diagrams of DEGs; PSGs and genes of skyblue3 module. A-D represents the Venn diagram of GD1, GD3, GD5, and GD8, respectively.

## Data Availability

The datasets generated and/or analysed during the current study are available in the NCBI repository, https://www.ncbi.nlm.nih.gov/bioproject/PRJNA554555.

## Abbreviations

WGCNA: Weighted gene co-expression network analysis
NCBI: National Center for Biotechnology Information
PSG: Positively selected genes
DEG: differentiated expressed genes
SNPs: Single Nucleotide Polymorphisms
