# Supplementary material for: Comparative transcriptome analysis reveals evolutionary divergence and shared network of cold and salt stress response in diploid D-genome cotton
Source: BMC Plant Biol. 2020 Nov 12;20:518. doi: 10.1186/s12870-020-02726-4 (PMC7664088; doi:10.1186/s12870-020-02726-4)
Supplement: Supplementary file 1 — Additional files 1: Table S1. Summary of RNA-seq data. [file 12870_2020_2726_MOESM1_ESM.docx]

Table S1 Summary of RNA-seq data

| **BMK-ID** | **Total Reads** | **GC Content** | **%≥Q30** | **Mapped Reads** | **Uniq Mapped Reads** | **Multiple Map Reads** | **Reads Map to '+'** | **Reads Map to '-'** |
| --- | --- | --- | --- | --- | --- | --- | --- | --- |
| GD1C0R1 | 43,671,536 | 45.91% | 91.59% | 38,542,782 (88.26%) | 37,160,605 (85.09%) | 1,382,177 (3.16%) | 18,920,897 (43.33%) | 18,990,613 (43.49%) |
| GD1C0R2 | 59,275,298 | 45.40% | 93.84% | 50,194,073 (84.68%) | 48,591,149 (81.98%) | 1,602,924 (2.70%) | 24,794,573 (41.83%) | 24,893,069 (42.00%) |
| GD1C12R1 | 34,481,532 | 45.20% | 92.16% | 30,171,643 (87.50%) | 29,332,795 (85.07%) | 838,848 (2.43%) | 14,900,093 (43.21%) | 14,954,926 (43.37%) |
| GD1C12R2 | 66,524,388 | 45.15% | 96.32% | 57,661,387 (86.68%) | 55,972,728 (84.14%) | 1,688,659 (2.54%) | 28,533,260 (42.89%) | 28,581,471 (42.96%) |
| GD1C6R1 | 43,628,618 | 45.95% | 91.33% | 38,240,781 (87.65%) | 37,004,315 (84.82%) | 1,236,466 (2.83%) | 18,818,662 (43.13%) | 18,893,242 (43.30%) |
| GD1C6R2 | 56,059,064 | 45.47% | 96.03% | 48,464,336 (86.45%) | 47,144,313 (84.10%) | 1,320,023 (2.35%) | 24,012,972 (42.84%) | 24,066,893 (42.93%) |
| GD1S12 | 60,403,146 | 45.33% | 96.35% | 51,416,709 (85.12%) | 50,251,450 (83.19%) | 1,165,259 (1.93%) | 25,607,990 (42.40%) | 25,650,642 (42.47%) |
| GD1S6 | 44,193,634 | 44.67% | 95.42% | 38,576,648 (87.29%) | 37,562,089 (84.99%) | 1,014,559 (2.30%) | 19,132,302 (43.29%) | 19,186,435 (43.41%) |
| GD1T12 | 34,985,424 | 46.20% | 92.02% | 29,636,672 (84.71%) | 28,637,437 (81.86%) | 999,235 (2.86%) | 14,568,150 (41.64%) | 14,656,343 (41.89%) |
| GD1T6 | 41,889,552 | 45.75% | 92.03% | 36,235,144 (86.50%) | 35,017,725 (83.60%) | 1,217,419 (2.91%) | 17,823,326 (42.55%) | 17,896,916 (42.72%) |
| GD3C0R1 | 41,871,566 | 44.75% | 89.03% | 36,194,760 (86.44%) | 35,040,899 (83.69%) | 1,153,861 (2.76%) | 17,811,243 (42.54%) | 17,878,676 (42.70%) |
| GD3C0R2 | 71,442,776 | 44.73% | 96.42% | 62,984,468 (88.16%) | 61,100,536 (85.52%) | 1,883,932 (2.64%) | 31,141,180 (43.59%) | 31,174,938 (43.64%) |
| GD3C12R1 | 34,258,174 | 45.31% | 92.30% | 29,753,655 (86.85%) | 29,007,688 (84.67%) | 745,967 (2.18%) | 14,732,130 (43.00%) | 14,774,396 (43.13%) |
| GD3C12R2 | 51,584,892 | 44.98% | 94.24% | 43,956,624 (85.21%) | 42,902,444 (83.17%) | 1,054,180 (2.04%) | 21,824,303 (42.31%) | 21,873,426 (42.40%) |
| GD3C6R1 | 34,112,568 | 45.23% | 91.56% | 29,458,604 (86.36%) | 28,653,514 (84.00%) | 805,090 (2.36%) | 14,565,782 (42.70%) | 14,612,504 (42.84%) |
| GD3C6R2 | 60,582,444 | 44.95% | 96.33% | 52,855,971 (87.25%) | 51,494,110 (85.00%) | 1,361,861 (2.25%) | 26,197,387 (43.24%) | 26,239,467 (43.31%) |
| GD3S12 | 58,348,558 | 44.92% | 94.12% | 48,613,926 (83.32%) | 47,475,583 (81.37%) | 1,138,343 (1.95%) | 24,194,658 (41.47%) | 24,251,062 (41.56%) |
| GD3S6 | 53,608,076 | 44.41% | 96.46% | 46,959,168 (87.60%) | 45,746,921 (85.34%) | 1,212,247 (2.26%) | 23,378,761 (43.61%) | 23,396,773 (43.64%) |
| GD3T12 | 35,471,730 | 45.99% | 91.82% | 30,138,477 (84.96%) | 29,240,992 (82.43%) | 897,485 (2.53%) | 14,878,163 (41.94%) | 14,953,889 (42.16%) |
| GD3T6 | 49,693,390 | 45.01% | 92.13% | 43,976,606 (88.50%) | 42,694,983 (85.92%) | 1,281,623 (2.58%) | 21,733,649 (43.74%) | 21,806,671 (43.88%) |
| GD5C0R1 | 43,358,352 | 45.17% | 91.79% | 40,328,665 (93.01%) | 39,221,515 (90.46%) | 1,107,150 (2.55%) | 19,958,662 (46.03%) | 19,996,414 (46.12%) |
| GD5C0R2 | 69,848,300 | 45.30% | 93.67% | 63,587,566 (91.04%) | 61,619,726 (88.22%) | 1,967,840 (2.82%) | 31,358,686 (44.90%) | 31,486,310 (45.08%) |
| GD5C12R1 | 36,230,744 | 44.48% | 92.23% | 33,892,054 (93.55%) | 33,102,474 (91.37%) | 789,580 (2.18%) | 16,854,857 (46.52%) | 16,880,540 (46.59%) |
| GD5C12R2 | 58,520,840 | 45.23% | 96.57% | 53,841,315 (92.00%) | 52,463,993 (89.65%) | 1,377,322 (2.35%) | 26,735,797 (45.69%) | 26,788,505 (45.78%) |
| GD5C6R1 | 36,889,984 | 45.15% | 92.09% | 34,475,097 (93.45%) | 33,670,333 (91.27%) | 804,764 (2.18%) | 17,118,111 (46.40%) | 17,156,930 (46.51%) |
| GD5C6R2 | 65,344,428 | 45.74% | 96.41% | 58,079,053 (88.88%) | 56,364,379 (86.26%) | 1,714,674 (2.62%) | 28,724,619 (43.96%) | 28,809,905 (44.09%) |
| GD5S12 | 67,444,606 | 44.52% | 94.53% | 62,946,886 (93.33%) | 61,529,098 (91.23%) | 1,417,788 (2.10%) | 31,290,827 (46.39%) | 31,360,578 (46.50%) |
| GD5S6 | 44,584,732 | 44.66% | 96.38% | 41,589,081 (93.28%) | 40,615,550 (91.10%) | 973,531 (2.18%) | 20,681,857 (46.39%) | 20,709,656 (46.45%) |
| GD5T12 | 38,793,502 | 45.66% | 92.37% | 35,172,945 (90.67%) | 34,213,712 (88.19%) | 959,233 (2.47%) | 17,435,362 (44.94%) | 17,476,550 (45.05%) |
| GD5T6 | 37,430,510 | 45.45% | 92.41% | 34,497,921 (92.17%) | 33,570,715 (89.69%) | 927,206 (2.48%) | 17,084,084 (45.64%) | 17,121,982 (45.74%) |
| GD8C0R1 | 40,083,638 | 46.64% | 91.66% | 34,922,429 (87.12%) | 33,563,987 (83.73%) | 1,358,442 (3.39%) | 17,092,332 (42.64%) | 17,178,078 (42.86%) |
| GD8C0R2 | 61,651,140 | 45.39% | 95.85% | 53,807,917 (87.28%) | 51,622,202 (83.73%) | 2,185,715 (3.55%) | 26,343,361 (42.73%) | 26,437,069 (42.88%) |
| GD8C12R1 | 41,639,650 | 45.32% | 91.94% | 36,014,414 (86.49%) | 34,845,958 (83.68%) | 1,168,456 (2.81%) | 17,772,578 (42.68%) | 17,822,127 (42.80%) |
| GD8C12R2 | 62,803,252 | 44.84% | 96.32% | 55,104,955 (87.74%) | 53,442,493 (85.10%) | 1,662,462 (2.65%) | 27,225,488 (43.35%) | 27,295,898 (43.46%) |
| GD8C6R1 | 32,771,674 | 45.60% | 92.31% | 28,593,074 (87.25%) | 27,856,903 (85.00%) | 736,171 (2.25%) | 14,158,396 (43.20%) | 14,215,523 (43.38%) |
| GD8C6R2 | 55,230,440 | 45.54% | 96.40% | 47,772,665 (86.50%) | 46,391,495 (84.00%) | 1,381,170 (2.50%) | 23,630,126 (42.78%) | 23,687,422 (42.89%) |
| GD8S12 | 62,225,106 | 45.05% | 96.22% | 53,147,203 (85.41%) | 51,538,896 (82.83%) | 1,608,307 (2.58%) | 26,329,941 (42.31%) | 26,380,324 (42.39%) |
| GD8S6 | 47,368,988 | 45.46% | 93.72% | 39,536,782 (83.47%) | 38,327,652 (80.91%) | 1,209,130 (2.55%) | 19,557,092 (41.29%) | 19,610,684 (41.40%) |
| GD8T12 | 38,520,342 | 45.91% | 92.22% | 33,721,897 (87.54%) | 32,557,315 (84.52%) | 1,164,582 (3.02%) | 16,547,169 (42.96%) | 16,647,895 (43.22%) |
| GD8T6 | 42,423,350 | 45.68% | 91.55% | 37,487,409 (88.37%) | 36,166,657 (85.25%) | 1,320,752 (3.11%) | 18,403,190 (43.38%) | 18,481,599 (43.56%) |
