# Supplementary material for: Comparative transcriptome analysis reveals evolutionary divergence and shared network of cold and salt stress response in diploid D-genome cotton
Source: BMC Plant Biol. 2020 Nov 12;20:518. doi: 10.1186/s12870-020-02726-4 (PMC7664088; doi:10.1186/s12870-020-02726-4)
Supplement: Supplementary file 2 — Additional files 2: Table S2. SNPs information of all samples. [file 12870_2020_2726_MOESM2_ESM.docx]

Table S2 SNPs information of all samples

| BMK-ID | SNP Number | Genic SNP | Intergenic SNP | Transition | Transversion | Heterozygosity |
| --- | --- | --- | --- | --- | --- | --- |
| GD1C0R1 | 284431 | 266510 | 17921 | 60.63% | 39.37% | 2.99% |
| GD1C0R2 | 325656 | 300975 | 24681 | 60.04% | 39.96% | 2.40% |
| GD1C12R1 | 288308 | 270461 | 17847 | 60.37% | 39.63% | 2.92% |
| GD1C12R2 | 357982 | 328222 | 29760 | 59.96% | 40.04% | 3.80% |
| GD1C6R1 | 291708 | 273279 | 18429 | 60.42% | 39.58% | 2.64% |
| GD1C6R2 | 338341 | 313223 | 25118 | 60.06% | 39.94% | 3.75% |
| GD1S12 | 346216 | 318510 | 27706 | 60.00% | 40.00% | 3.80% |
| GD1S6 | 344903 | 320389 | 24514 | 59.96% | 40.04% | 1.97% |
| GD1T12 | 273931 | 257697 | 16234 | 60.38% | 39.62% | 3.01% |
| GD1T6 | 291494 | 273502 | 17992 | 60.22% | 39.78% | 3.80% |
| GD3C0R1 | 314559 | 292852 | 21707 | 60.31% | 39.69% | 3.87% |
| GD3C0R2 | 392737 | 359967 | 32770 | 59.86% | 40.14% | 5.88% |
| GD3C12R1 | 308799 | 289285 | 19514 | 60.43% | 39.57% | 2.95% |
| GD3C12R2 | 371523 | 341935 | 29588 | 60.12% | 39.88% | 3.73% |
| GD3C6R1 | 300146 | 281584 | 18562 | 60.62% | 39.38% | 2.80% |
| GD3C6R2 | 381903 | 351479 | 30424 | 60.03% | 39.97% | 2.39% |
| GD3S12 | 395664 | 360790 | 34874 | 60.03% | 39.97% | 3.95% |
| GD3S6 | 374548 | 344354 | 30194 | 59.96% | 40.04% | 2.35% |
| GD3T12 | 310236 | 292438 | 17798 | 60.21% | 39.79% | 3.10% |
| GD3T6 | 345741 | 323080 | 22661 | 60.01% | 39.99% | 3.33% |
| GD5C0R1 | 8925 | 6775 | 2150 | 64.40% | 35.60% | 31.78% |
| GD5C0R2 | 10335 | 7235 | 3100 | 64.62% | 35.38% | 23.21% |
| GD5C12R1 | 7815 | 5869 | 1946 | 65.21% | 34.79% | 27.34% |
| GD5C12R2 | 15849 | 12453 | 3396 | 63.18% | 36.82% | 44.23% |
| GD5C6R1 | 7420 | 5358 | 2062 | 65.57% | 34.43% | 22.02% |
| GD5C6R2 | 10428 | 7212 | 3216 | 64.95% | 35.05% | 24.19% |
| GD5S12 | 14849 | 11200 | 3649 | 63.59% | 36.41% | 35.83% |
| GD5S6 | 8438 | 6029 | 2409 | 64.74% | 35.26% | 21.70% |
| GD5T12 | 8935 | 6506 | 2429 | 65.46% | 34.54% | 28.15% |
| GD5T6 | 7374 | 5329 | 2045 | 65.84% | 34.16% | 21.43% |
| GD8C0R1 | 267580 | 252393 | 15187 | 60.44% | 39.56% | 3.89% |
| GD8C0R2 | 336299 | 311357 | 24942 | 59.79% | 40.21% | 3.06% |
| GD8C12R1 | 308865 | 287865 | 21000 | 60.28% | 39.72% | 2.99% |
| GD8C12R2 | 404737 | 371636 | 33101 | 60.02% | 39.98% | 24.94% |
| GD8C6R1 | 277124 | 260541 | 16583 | 60.31% | 39.69% | 2.90% |
| GD8C6R2 | 348626 | 322695 | 25931 | 59.84% | 40.16% | 3.44% |
| GD8S12 | 359243 | 328483 | 30760 | 59.84% | 40.16% | 3.63% |
| GD8S6 | 345302 | 319120 | 26182 | 59.88% | 40.12% | 2.28% |
| GD8T12 | 283506 | 268421 | 15085 | 60.12% | 39.88% | 2.89% |
| GD8T6 | 292320 | 275136 | 17184 | 60.17% | 39.83% | 2.41% |
