# Supplementary material for: Comparative transcriptome analysis reveals evolutionary divergence and shared network of cold and salt stress response in diploid D-genome cotton
Source: BMC Plant Biol. 2020 Nov 12;20:518. doi: 10.1186/s12870-020-02726-4 (PMC7664088; doi:10.1186/s12870-020-02726-4)
Supplement: Supplementary file 3 — Additional files 3: Table S3. GO enrichment of high functional effects by SNPs in four diploid D-genome kinds of cotton. [file 12870_2020_2726_MOESM3_ESM.docx]

Table S3 GO enrichment of high functional effects by SNPs in four diploid D-genome cottons

| GO_acc | term_type | Term | queryitem | querytotal | bgitem | bgtotal | pvalue |
| --- | --- | --- | --- | --- | --- | --- | --- |
| GO:0032501 | P | multicellular organismal process | 143 | 17430 | 175 | 52012 | 1.1E-14 |
| GO:0042221 | P | response to chemical | 241 | 17430 | 395 | 52012 | 5.8E-13 |
| GO:0006464 | P | cellular protein modification process | 1875 | 17430 | 4679 | 52012 | 5.9E-12 |
| GO:0036211 | P | protein modification process | 1875 | 17430 | 4679 | 52012 | 5.9E-12 |
| GO:0043412 | P | macromolecule modification | 1920 | 17430 | 4833 | 52012 | 2.8E-11 |
| GO:0044260 | P | cellular macromolecule metabolic process | 4529 | 17430 | 12324 | 52012 | 6.7E-10 |
| GO:0044267 | P | cellular protein metabolic process | 2645 | 17430 | 6950 | 52012 | 1.4E-09 |
| GO:0043170 | P | macromolecule metabolic process | 4953 | 17430 | 13664 | 52012 | 1.9E-08 |
| GO:0070647 | P | protein modification by small protein conjugation or removal | 128 | 17430 | 203 | 52012 | 3.4E-08 |
| GO:0019538 | P | protein metabolic process | 3032 | 17430 | 8223 | 52012 | 5.5E-07 |
| GO:0055114 | P | oxidation-reduction process | 1478 | 17430 | 3824 | 52012 | 8.5E-07 |
| GO:0044702 | P | single organism reproductive process | 80 | 17430 | 121 | 52012 | 3.2E-06 |
| GO:0006468 | P | protein phosphorylation | 1410 | 17430 | 3669 | 52012 | 3.7E-06 |
| GO:0015979 | P | photosynthesis | 136 | 17430 | 250 | 52012 | 5.7E-06 |
| GO:0007015 | P | actin filament organization | 42 | 17430 | 49 | 52012 | 0.000011 |
| GO:0000375 | P | RNA splicing, via transesterification reactions | 35 | 17430 | 38 | 52012 | 0.000021 |
| GO:0000377 | P | RNA splicing, via transesterification reactions with bulged adenosine as nucleophile | 35 | 17430 | 38 | 52012 | 0.000021 |
| GO:0000398 | P | mRNA splicing, via spliceosome | 35 | 17430 | 38 | 52012 | 0.000021 |
| GO:0032446 | P | protein modification by small protein conjugation | 112 | 17430 | 203 | 52012 | 0.000023 |
| GO:0008380 | P | RNA splicing | 53 | 17430 | 73 | 52012 | 0.000023 |
| GO:0016567 | P | protein ubiquitination | 109 | 17430 | 198 | 52012 | 0.000031 |
| GO:0000003 | P | reproduction | 80 | 17430 | 135 | 52012 | 0.000055 |
| GO:0032984 | P | macromolecular complex disassembly | 42 | 17430 | 54 | 52012 | 0.000052 |
| GO:0043241 | P | protein complex disassembly | 42 | 17430 | 54 | 52012 | 0.000052 |
| GO:0008037 | P | cell recognition | 70 | 17430 | 113 | 52012 | 0.000058 |
| GO:0009856 | P | pollination | 70 | 17430 | 113 | 52012 | 0.000058 |
| GO:0022414 | P | reproductive process | 80 | 17430 | 135 | 52012 | 0.000055 |
| GO:0022411 | P | cellular component disassembly | 42 | 17430 | 54 | 52012 | 0.000052 |
| GO:0043624 | P | cellular protein complex disassembly | 42 | 17430 | 54 | 52012 | 0.000052 |
| GO:0044706 | P | multi-multicellular organism process | 70 | 17430 | 113 | 52012 | 0.000058 |
| GO:0048544 | P | recognition of pollen | 70 | 17430 | 113 | 52012 | 0.000058 |
| GO:0009875 | P | pollen-pistil interaction | 70 | 17430 | 113 | 52012 | 0.000058 |
| GO:0008152 | P | metabolic process | 8406 | 17430 | 24203 | 52012 | 0.000055 |
| GO:0000278 | P | mitotic cell cycle | 35 | 17430 | 42 | 52012 | 0.000083 |
| GO:0010467 | P | gene expression | 2235 | 17430 | 6121 | 52012 | 0.00012 |
| GO:1903047 | P | mitotic cell cycle process | 34 | 17430 | 42 | 52012 | 0.00015 |
| GO:0051704 | P | multi-organism process | 82 | 17430 | 146 | 52012 | 0.00017 |
| GO:0042545 | P | cell wall modification | 62 | 17430 | 101 | 52012 | 0.00018 |
| GO:0008154 | P | actin polymerization or depolymerization | 37 | 17430 | 49 | 52012 | 0.00021 |
| GO:0048519 | P | negative regulation of biological process | 51 | 17430 | 80 | 52012 | 0.00033 |
| GO:0007017 | P | microtubule-based process | 177 | 17430 | 385 | 52012 | 0.00036 |
| GO:0044703 | P | multi-organism reproductive process | 70 | 17430 | 127 | 52012 | 0.00071 |
| GO:0015849 | P | organic acid transport | 37 | 17430 | 54 | 52012 | 0.0008 |
| GO:1905039 | P | carboxylic acid transmembrane transport | 37 | 17430 | 54 | 52012 | 0.0008 |
| GO:0003333 | P | amino acid transmembrane transport | 37 | 17430 | 54 | 52012 | 0.0008 |
| GO:1903825 | P | organic acid transmembrane transport | 37 | 17430 | 54 | 52012 | 0.0008 |
| GO:0006310 | P | DNA recombination | 45 | 17430 | 71 | 52012 | 0.00078 |
| GO:0016310 | P | phosphorylation | 1509 | 17430 | 4109 | 52012 | 0.00085 |
| GO:0055082 | P | cellular chemical homeostasis | 18 | 17430 | 18 | 52012 | 0.0011 |
| GO:0006470 | P | protein dephosphorylation | 145 | 17430 | 317 | 52012 | 0.0013 |
| GO:0009607 | P | response to biotic stimulus | 46 | 17430 | 76 | 52012 | 0.0014 |
| GO:0019725 | P | cellular homeostasis | 158 | 17430 | 351 | 52012 | 0.0014 |
| GO:0007049 | P | cell cycle | 113 | 17430 | 237 | 52012 | 0.0015 |
| GO:0007059 | P | chromosome segregation | 22 | 17430 | 26 | 52012 | 0.0015 |
| GO:0071840 | P | cellular component organization or biogenesis | 501 | 17430 | 1282 | 52012 | 0.0019 |
| GO:0045786 | P | negative regulation of cell cycle | 18 | 17430 | 20 | 52012 | 0.0024 |
| GO:0006996 | P | organelle organization | 276 | 17430 | 671 | 52012 | 0.0025 |
| GO:1902589 | P | single-organism organelle organization | 102 | 17430 | 216 | 52012 | 0.003 |
| GO:0010468 | P | regulation of gene expression | 1235 | 17430 | 3371 | 52012 | 0.0031 |
| GO:0048285 | P | organelle fission | 32 | 17430 | 50 | 52012 | 0.0038 |
| GO:0019684 | P | photosynthesis, light reaction | 50 | 17430 | 91 | 52012 | 0.0039 |
| GO:0006979 | P | response to oxidative stress | 109 | 17430 | 237 | 52012 | 0.0042 |
| GO:0000280 | P | nuclear division | 26 | 17430 | 38 | 52012 | 0.0046 |
| GO:0045017 | P | glycerolipid biosynthetic process | 41 | 17430 | 71 | 52012 | 0.0045 |
| GO:0045229 | P | external encapsulating structure organization | 80 | 17430 | 165 | 52012 | 0.0047 |
| GO:0071555 | P | cell wall organization | 80 | 17430 | 165 | 52012 | 0.0047 |
| GO:0007067 | P | mitotic nuclear division | 22 | 17430 | 30 | 52012 | 0.0049 |
| GO:0009059 | P | macromolecule biosynthetic process | 2178 | 17430 | 6117 | 52012 | 0.0051 |
| GO:0048518 | P | positive regulation of biological process | 48 | 17430 | 88 | 52012 | 0.0052 |
| GO:0034645 | P | cellular macromolecule biosynthetic process | 2177 | 17430 | 6113 | 52012 | 0.005 |
| GO:0045454 | P | cell redox homeostasis | 139 | 17430 | 319 | 52012 | 0.0062 |
| GO:0016043 | P | cellular component organization | 448 | 17430 | 1162 | 52012 | 0.0062 |
| GO:0065008 | P | regulation of biological quality | 214 | 17430 | 521 | 52012 | 0.0072 |
| GO:0032774 | P | RNA biosynthetic process | 1348 | 17430 | 3730 | 52012 | 0.0073 |
| GO:0097659 | P | nucleic acid-templated transcription | 1346 | 17430 | 3727 | 52012 | 0.0078 |
| GO:0006351 | P | transcription, DNA-templated | 1346 | 17430 | 3727 | 52012 | 0.0078 |
| GO:0006914 | P | autophagy | 19 | 17430 | 26 | 52012 | 0.0089 |
| GO:0050789 | P | regulation of biological process | 1828 | 17430 | 5130 | 52012 | 0.0093 |
| GO:0019219 | P | regulation of nucleobase-containing compound metabolic process | 1208 | 17430 | 3339 | 52012 | 0.0099 |
| GO:0009765 | P | photosynthesis, light harvesting | 27 | 17430 | 44 | 52012 | 0.011 |
| GO:0010556 | P | regulation of macromolecule biosynthetic process | 1216 | 17430 | 3366 | 52012 | 0.011 |
| GO:2000112 | P | regulation of cellular macromolecule biosynthetic process | 1216 | 17430 | 3366 | 52012 | 0.011 |
| GO:0009889 | P | regulation of biosynthetic process | 1218 | 17430 | 3376 | 52012 | 0.012 |
| GO:0051171 | P | regulation of nitrogen compound metabolic process | 1223 | 17430 | 3389 | 52012 | 0.012 |
| GO:0051247 | P | positive regulation of protein metabolic process | 18 | 17430 | 25 | 52012 | 0.012 |
| GO:0032270 | P | positive regulation of cellular protein metabolic process | 18 | 17430 | 25 | 52012 | 0.012 |
| GO:0031326 | P | regulation of cellular biosynthetic process | 1218 | 17430 | 3376 | 52012 | 0.012 |
| GO:0006541 | P | glutamine metabolic process | 19 | 17430 | 27 | 52012 | 0.012 |
| GO:0042592 | P | homeostatic process | 164 | 17430 | 396 | 52012 | 0.013 |
| GO:2001141 | P | regulation of RNA biosynthetic process | 1197 | 17430 | 3321 | 52012 | 0.014 |
| GO:0019222 | P | regulation of metabolic process | 1270 | 17430 | 3531 | 52012 | 0.013 |
| GO:0006355 | P | regulation of transcription, DNA-templated | 1197 | 17430 | 3321 | 52012 | 0.014 |
| GO:1903506 | P | regulation of nucleic acid-templated transcription | 1197 | 17430 | 3321 | 52012 | 0.014 |
| GO:0007010 | P | cytoskeleton organization | 89 | 17430 | 198 | 52012 | 0.014 |
| GO:0051052 | P | regulation of DNA metabolic process | 10 | 17430 | 10 | 52012 | 0.014 |
| GO:0022613 | P | ribonucleoprotein complex biogenesis | 61 | 17430 | 127 | 52012 | 0.014 |
| GO:0051169 | P | nuclear transport | 15 | 17430 | 223 | 52012 | 1 |
| GO:0046434 | P | organophosphate catabolic process | 6 | 17430 | 23 | 52012 | 0.77 |
| GO:0006904 | P | vesicle docking involved in exocytosis | 12 | 17430 | 39 | 52012 | 0.65 |
| GO:0046348 | P | amino sugar catabolic process | 15 | 17430 | 37 | 52012 | 0.31 |
| GO:0008064 | P | regulation of actin polymerization or depolymerization | 20 | 17430 | 47 | 52012 | 0.22 |
| GO:0009167 | P | purine ribonucleoside monophosphate metabolic process | 121 | 17430 | 515 | 52012 | 1 |
| GO:0009165 | P | nucleotide biosynthetic process | 96 | 17430 | 380 | 52012 | 1 |
| GO:0009690 | P | cytokinin metabolic process | 12 | 17430 | 22 | 52012 | 0.12 |
| GO:0044281 | P | small molecule metabolic process | 722 | 17430 | 2983 | 52012 | 1 |
| GO:0044282 | P | small molecule catabolic process | 39 | 17430 | 116 | 52012 | 0.52 |
| GO:0044283 | P | small molecule biosynthetic process | 198 | 17430 | 910 | 52012 | 1 |
| GO:0098771 | P | inorganic ion homeostasis | 5 | 17430 | 49 | 52012 | 1 |
| GO:0051656 | P | establishment of organelle localization | 5 | 17430 | 9 | 52012 | 0.26 |
| GO:0051651 | P | maintenance of location in cell | 11 | 17430 | 34 | 52012 | 0.6 |
| GO:0009168 | P | purine ribonucleoside monophosphate biosynthetic process | 53 | 17430 | 251 | 52012 | 1 |
| GO:0051716 | P | cellular response to stimulus | 542 | 17430 | 1838 | 52012 | 1 |
| GO:0009135 | P | purine nucleoside diphosphate metabolic process | 50 | 17430 | 187 | 52012 | 0.94 |
| GO:0006771 | P | riboflavin metabolic process | 9 | 17430 | 37 | 52012 | 0.85 |
| GO:0051493 | P | regulation of cytoskeleton organization | 21 | 17430 | 47 | 52012 | 0.17 |
| GO:0016108 | P | tetraterpenoid metabolic process | 5 | 17430 | 23 | 52012 | 0.87 |
| GO:0016109 | P | tetraterpenoid biosynthetic process | 5 | 17430 | 23 | 52012 | 0.87 |
| GO:0009199 | P | ribonucleoside triphosphate metabolic process | 118 | 17430 | 488 | 52012 | 1 |
| GO:0015696 | P | ammonium transport | 10 | 17430 | 17 | 52012 | 0.12 |
| GO:0051049 | P | regulation of transport | 6 | 17430 | 38 | 52012 | 0.98 |
| GO:0065007 | P | biological regulation | 1885 | 17430 | 5353 | 52012 | 0.026 |
| GO:0007088 | P | regulation of mitotic nuclear division | 7 | 17430 | 7 | 52012 | 0.039 |
| GO:0046500 | P | S-adenosylmethionine metabolic process | 8 | 17430 | 18 | 52012 | 0.32 |
| GO:0033044 | P | regulation of chromosome organization | 7 | 17430 | 7 | 52012 | 0.039 |
| GO:0006284 | P | base-excision repair | 20 | 17430 | 80 | 52012 | 0.9 |
| GO:0006281 | P | DNA repair | 157 | 17430 | 484 | 52012 | 0.65 |
| GO:0045859 | P | regulation of protein kinase activity | 20 | 17430 | 130 | 52012 | 1 |
| GO:0031109 | P | microtubule polymerization or depolymerization | 5 | 17430 | 7 | 52012 | 0.16 |
| GO:0006289 | P | nucleotide-excision repair | 17 | 17430 | 92 | 52012 | 0.99 |
| GO:0050832 | P | defense response to fungus | 5 | 17430 | 7 | 52012 | 0.16 |
| GO:0046488 | P | phosphatidylinositol metabolic process | 67 | 17430 | 273 | 52012 | 0.99 |
| GO:0006839 | P | mitochondrial transport | 18 | 17430 | 42 | 52012 | 0.23 |
| GO:0046486 | P | glycerolipid metabolic process | 81 | 17430 | 280 | 52012 | 0.89 |
| GO:0046483 | P | heterocycle metabolic process | 2247 | 17430 | 6782 | 52012 | 0.7 |
| GO:0042325 | P | regulation of phosphorylation | 24 | 17430 | 141 | 52012 | 1 |
| GO:0006575 | P | cellular modified amino acid metabolic process | 16 | 17430 | 74 | 52012 | 0.96 |
| GO:0006576 | P | cellular biogenic amine metabolic process | 24 | 17430 | 52 | 52012 | 0.12 |
| GO:0006270 | P | DNA replication initiation | 7 | 17430 | 24 | 52012 | 0.69 |
| GO:0051783 | P | regulation of nuclear division | 7 | 17430 | 7 | 52012 | 0.039 |
| GO:0009163 | P | nucleoside biosynthetic process | 56 | 17430 | 250 | 52012 | 1 |
| GO:0006452 | P | translational frameshifting | 10 | 17430 | 25 | 52012 | 0.38 |
| GO:0009892 | P | negative regulation of metabolic process | 28 | 17430 | 56 | 52012 | 0.056 |
| GO:0009893 | P | positive regulation of metabolic process | 22 | 17430 | 80 | 52012 | 0.83 |
| GO:0009890 | P | negative regulation of biosynthetic process | 10 | 17430 | 46 | 52012 | 0.92 |
| GO:0009891 | P | positive regulation of biosynthetic process | 13 | 17430 | 80 | 52012 | 1 |
| GO:0009161 | P | ribonucleoside monophosphate metabolic process | 121 | 17430 | 537 | 52012 | 1 |
| GO:0006596 | P | polyamine biosynthetic process | 11 | 17430 | 17 | 52012 | 0.07 |
| GO:0006597 | P | spermine biosynthetic process | 7 | 17430 | 8 | 52012 | 0.058 |
| GO:0017004 | P | cytochrome complex assembly | 8 | 17430 | 16 | 52012 | 0.24 |
| GO:0006595 | P | polyamine metabolic process | 11 | 17430 | 17 | 52012 | 0.07 |
| GO:0044036 | P | cell wall macromolecule metabolic process | 19 | 17430 | 69 | 52012 | 0.81 |
| GO:1901269 | P | lipooligosaccharide metabolic process | 8 | 17430 | 25 | 52012 | 0.61 |
| GO:0051220 | P | cytoplasmic sequestering of protein | 11 | 17430 | 34 | 52012 | 0.6 |
| GO:0035556 | P | intracellular signal transduction | 209 | 17430 | 863 | 52012 | 1 |
| GO:0071900 | P | regulation of protein serine/threonine kinase activity | 12 | 17430 | 130 | 52012 | 1 |
| GO:1901264 | P | carbohydrate derivative transport | 6 | 17430 | 27 | 52012 | 0.87 |
| GO:0072350 | P | tricarboxylic acid metabolic process | 9 | 17430 | 32 | 52012 | 0.73 |
| GO:0015992 | P | proton transport | 110 | 17430 | 270 | 52012 | 0.049 |
| GO:0015991 | P | ATP hydrolysis coupled proton transport | 51 | 17430 | 146 | 52012 | 0.43 |
| GO:0006886 | P | intracellular protein transport | 197 | 17430 | 991 | 52012 | 1 |
| GO:0006887 | P | exocytosis | 39 | 17430 | 81 | 52012 | 0.041 |
| GO:0042254 | P | ribosome biogenesis | 52 | 17430 | 123 | 52012 | 0.095 |
| GO:0042558 | P | pteridine-containing compound metabolic process | 12 | 17430 | 57 | 52012 | 0.95 |
| GO:0042559 | P | pteridine-containing compound biosynthetic process | 10 | 17430 | 50 | 52012 | 0.96 |
| GO:0046132 | P | pyrimidine ribonucleoside biosynthetic process | 5 | 17430 | 15 | 52012 | 0.59 |
| GO:0046854 | P | phosphatidylinositol phosphorylation | 18 | 17430 | 142 | 52012 | 1 |
| GO:0042822 | P | pyridoxal phosphate metabolic process | 6 | 17430 | 13 | 52012 | 0.34 |
| GO:0042823 | P | pyridoxal phosphate biosynthetic process | 6 | 17430 | 13 | 52012 | 0.34 |
| GO:0032259 | P | methylation | 55 | 17430 | 152 | 52012 | 0.34 |
| GO:0018130 | P | heterocycle biosynthetic process | 1524 | 17430 | 4465 | 52012 | 0.26 |
| GO:0006629 | P | lipid metabolic process | 479 | 17430 | 1561 | 52012 | 0.96 |
| GO:0009308 | P | amine metabolic process | 43 | 17430 | 133 | 52012 | 0.61 |
| GO:0009309 | P | amine biosynthetic process | 16 | 17430 | 30 | 52012 | 0.092 |
| GO:0006625 | P | protein targeting to peroxisome | 6 | 17430 | 22 | 52012 | 0.74 |
| GO:0006626 | P | protein targeting to mitochondrion | 11 | 17430 | 36 | 52012 | 0.66 |
| GO:0006621 | P | protein retention in ER lumen | 11 | 17430 | 27 | 52012 | 0.35 |
| GO:0007050 | P | cell cycle arrest | 10 | 17430 | 20 | 52012 | 0.2 |
| GO:0009260 | P | ribonucleotide biosynthetic process | 63 | 17430 | 280 | 52012 | 1 |
| GO:0009262 | P | deoxyribonucleotide metabolic process | 6 | 17430 | 25 | 52012 | 0.83 |
| GO:0043414 | P | macromolecule methylation | 52 | 17430 | 134 | 52012 | 0.21 |
| GO:0032957 | P | inositol trisphosphate metabolic process | 7 | 17430 | 28 | 52012 | 0.81 |
| GO:0009266 | P | response to temperature stimulus | 8 | 17430 | 48 | 52012 | 0.98 |
| GO:0044723 | P | single-organism carbohydrate metabolic process | 253 | 17430 | 1036 | 52012 | 1 |
| GO:0019395 | P | fatty acid oxidation | 11 | 17430 | 21 | 52012 | 0.16 |
| GO:0006586 | P | indolalkylamine metabolic process | 9 | 17430 | 30 | 52012 | 0.67 |
| GO:0042430 | P | indole-containing compound metabolic process | 9 | 17430 | 30 | 52012 | 0.67 |
| GO:0044724 | P | single-organism carbohydrate catabolic process | 58 | 17430 | 216 | 52012 | 0.94 |
| GO:0044042 | P | glucan metabolic process | 83 | 17430 | 280 | 52012 | 0.85 |
| GO:0016071 | P | mRNA metabolic process | 75 | 17430 | 179 | 52012 | 0.061 |
| GO:0016072 | P | rRNA metabolic process | 24 | 17430 | 82 | 52012 | 0.75 |
| GO:0006783 | P | heme biosynthetic process | 7 | 17430 | 26 | 52012 | 0.76 |
| GO:0010557 | P | positive regulation of macromolecule biosynthetic process | 13 | 17430 | 80 | 52012 | 1 |
| GO:1901293 | P | nucleoside phosphate biosynthetic process | 96 | 17430 | 385 | 52012 | 1 |
| GO:0000160 | P | phosphorelay signal transduction system | 73 | 17430 | 203 | 52012 | 0.32 |
| GO:0006022 | P | aminoglycan metabolic process | 18 | 17430 | 47 | 52012 | 0.36 |
| GO:0006020 | P | inositol metabolic process | 7 | 17430 | 32 | 52012 | 0.89 |
| GO:0006753 | P | nucleoside phosphate metabolic process | 198 | 17430 | 804 | 52012 | 1 |
| GO:0006026 | P | aminoglycan catabolic process | 15 | 17430 | 37 | 52012 | 0.31 |
| GO:0010558 | P | negative regulation of macromolecule biosynthetic process | 10 | 17430 | 46 | 52012 | 0.92 |
| GO:0006757 | P | ATP generation from ADP | 50 | 17430 | 187 | 52012 | 0.94 |
| GO:0034250 | P | positive regulation of cellular amide metabolic process | 10 | 17430 | 25 | 52012 | 0.38 |
| GO:0043413 | P | macromolecule glycosylation | 65 | 17430 | 179 | 52012 | 0.31 |
| GO:1902600 | P | hydrogen ion transmembrane transport | 89 | 17430 | 216 | 52012 | 0.059 |
| GO:0046578 | P | regulation of Ras protein signal transduction | 12 | 17430 | 33 | 52012 | 0.46 |
| GO:0051128 | P | regulation of cellular component organization | 40 | 17430 | 92 | 52012 | 0.1 |
| GO:0009416 | P | response to light stimulus | 10 | 17430 | 115 | 52012 | 1 |
| GO:0031497 | P | chromatin assembly | 25 | 17430 | 163 | 52012 | 1 |
| GO:0009415 | P | response to water | 6 | 17430 | 24 | 52012 | 0.8 |
| GO:0043244 | P | regulation of protein complex disassembly | 13 | 17430 | 25 | 52012 | 0.13 |
| GO:0006308 | P | DNA catabolic process | 7 | 17430 | 17 | 52012 | 0.4 |
| GO:0043243 | P | positive regulation of protein complex disassembly | 10 | 17430 | 25 | 52012 | 0.38 |
| GO:0009396 | P | folic acid-containing compound biosynthetic process | 8 | 17430 | 42 | 52012 | 0.96 |
| GO:0042727 | P | flavin-containing compound biosynthetic process | 9 | 17430 | 37 | 52012 | 0.85 |
| GO:0042726 | P | flavin-containing compound metabolic process | 9 | 17430 | 37 | 52012 | 0.85 |
| GO:0019318 | P | hexose metabolic process | 27 | 17430 | 173 | 52012 | 1 |
| GO:0009094 | P | L-phenylalanine biosynthetic process | 6 | 17430 | 19 | 52012 | 0.63 |
| GO:0009095 | P | aromatic amino acid family biosynthetic process, prephenate pathway | 9 | 17430 | 23 | 52012 | 0.41 |
| GO:0015672 | P | monovalent inorganic cation transport | 145 | 17430 | 494 | 52012 | 0.93 |
| GO:0006879 | P | cellular iron ion homeostasis | 5 | 17430 | 17 | 52012 | 0.68 |
| GO:0008283 | P | cell proliferation | 14 | 17430 | 18 | 52012 | 0.016 |
| GO:0006875 | P | cellular metal ion homeostasis | 5 | 17430 | 18 | 52012 | 0.72 |
| GO:0006072 | P | glycerol-3-phosphate metabolic process | 5 | 17430 | 34 | 52012 | 0.98 |
| GO:0006873 | P | cellular ion homeostasis | 10 | 17430 | 18 | 52012 | 0.14 |
| GO:0006974 | P | cellular response to DNA damage stimulus | 160 | 17430 | 485 | 52012 | 0.58 |
| GO:0034637 | P | cellular carbohydrate biosynthetic process | 76 | 17430 | 329 | 52012 | 1 |
| GO:0051156 | P | glucose 6-phosphate metabolic process | 10 | 17430 | 54 | 52012 | 0.98 |
| GO:0044257 | P | cellular protein catabolic process | 104 | 17430 | 427 | 52012 | 1 |
| GO:0044255 | P | cellular lipid metabolic process | 273 | 17430 | 886 | 52012 | 0.9 |
| GO:0009132 | P | nucleoside diphosphate metabolic process | 58 | 17430 | 214 | 52012 | 0.94 |
| GO:0030258 | P | lipid modification | 34 | 17430 | 197 | 52012 | 1 |
| GO:0030259 | P | lipid glycosylation | 5 | 17430 | 34 | 52012 | 0.98 |
| GO:0051983 | P | regulation of chromosome segregation | 7 | 17430 | 7 | 52012 | 0.039 |
| GO:0022406 | P | membrane docking | 16 | 17430 | 45 | 52012 | 0.47 |
| GO:0072511 | P | divalent inorganic cation transport | 14 | 17430 | 74 | 52012 | 0.99 |
| GO:0006338 | P | chromatin remodeling | 11 | 17430 | 25 | 52012 | 0.28 |
| GO:0006334 | P | nucleosome assembly | 25 | 17430 | 163 | 52012 | 1 |
| GO:0008654 | P | phospholipid biosynthetic process | 52 | 17430 | 149 | 52012 | 0.43 |
| GO:0006333 | P | chromatin assembly or disassembly | 26 | 17430 | 164 | 52012 | 1 |
| GO:0046474 | P | glycerophospholipid biosynthetic process | 29 | 17430 | 71 | 52012 | 0.21 |
| GO:0071941 | P | nitrogen cycle metabolic process | 6 | 17430 | 7 | 52012 | 0.082 |
| GO:0033866 | P | nucleoside bisphosphate biosynthetic process | 5 | 17430 | 19 | 52012 | 0.76 |
| GO:0015969 | P | guanosine tetraphosphate metabolic process | 6 | 17430 | 20 | 52012 | 0.67 |
| GO:0030036 | P | actin cytoskeleton organization | 47 | 17430 | 138 | 52012 | 0.49 |
| GO:0006099 | P | tricarboxylic acid cycle | 11 | 17430 | 61 | 52012 | 0.98 |
| GO:0006098 | P | pentose-phosphate shunt | 10 | 17430 | 54 | 52012 | 0.98 |
| GO:0031328 | P | positive regulation of cellular biosynthetic process | 13 | 17430 | 80 | 52012 | 1 |
| GO:0008216 | P | spermidine metabolic process | 10 | 17430 | 11 | 52012 | 0.021 |
| GO:0008215 | P | spermine metabolic process | 7 | 17430 | 8 | 52012 | 0.058 |
| GO:0051306 | P | mitotic sister chromatid separation | 7 | 17430 | 7 | 52012 | 0.039 |
| GO:0043631 | P | RNA polyadenylation | 12 | 17430 | 32 | 52012 | 0.43 |
| GO:0043632 | P | modification-dependent macromolecule catabolic process | 86 | 17430 | 369 | 52012 | 1 |
| GO:0006090 | P | pyruvate metabolic process | 57 | 17430 | 194 | 52012 | 0.83 |
| GO:0008219 | P | cell death | 5 | 17430 | 54 | 52012 | 1 |
| GO:0051301 | P | cell division | 16 | 17430 | 36 | 52012 | 0.21 |
| GO:0006094 | P | gluconeogenesis | 8 | 17430 | 28 | 52012 | 0.72 |
| GO:0009070 | P | serine family amino acid biosynthetic process | 7 | 17430 | 61 | 52012 | 1 |
| GO:0006529 | P | asparagine biosynthetic process | 8 | 17430 | 25 | 52012 | 0.61 |
| GO:0006528 | P | asparagine metabolic process | 8 | 17430 | 25 | 52012 | 0.61 |
| GO:0006486 | P | protein glycosylation | 65 | 17430 | 179 | 52012 | 0.31 |
| GO:0006487 | P | protein N-linked glycosylation | 13 | 17430 | 44 | 52012 | 0.7 |
| GO:0006396 | P | RNA processing | 214 | 17430 | 655 | 52012 | 0.64 |
| GO:0006520 | P | cellular amino acid metabolic process | 183 | 17430 | 899 | 52012 | 1 |
| GO:2000113 | P | negative regulation of cellular macromolecule biosynthetic process | 10 | 17430 | 46 | 52012 | 0.92 |
| GO:0006525 | P | arginine metabolic process | 6 | 17430 | 31 | 52012 | 0.93 |
| GO:0006096 | P | glycolytic process | 50 | 17430 | 187 | 52012 | 0.94 |
| GO:1990542 | P | mitochondrial transmembrane transport | 11 | 17430 | 35 | 52012 | 0.63 |
| GO:0006241 | P | CTP biosynthetic process | 5 | 17430 | 10 | 52012 | 0.32 |
| GO:1901071 | P | glucosamine-containing compound metabolic process | 15 | 17430 | 37 | 52012 | 0.31 |
| GO:0006461 | P | protein complex assembly | 83 | 17430 | 415 | 52012 | 1 |
| GO:0070085 | P | glycosylation | 70 | 17430 | 213 | 52012 | 0.58 |
| GO:0009220 | P | pyrimidine ribonucleotide biosynthetic process | 5 | 17430 | 15 | 52012 | 0.59 |
| GO:0006465 | P | signal peptide processing | 15 | 17430 | 41 | 52012 | 0.44 |
| GO:0044765 | P | single-organism transport | 382 | 17430 | 1208 | 52012 | 0.85 |
| GO:0044763 | P | single-organism cellular process | 2112 | 17430 | 7018 | 52012 | 1 |
| GO:0019941 | P | modification-dependent protein catabolic process | 86 | 17430 | 369 | 52012 | 1 |
| GO:0006091 | P | generation of precursor metabolites and energy | 132 | 17430 | 444 | 52012 | 0.9 |
| GO:0051273 | P | beta-glucan metabolic process | 46 | 17430 | 150 | 52012 | 0.73 |
| GO:0051276 | P | chromosome organization | 112 | 17430 | 322 | 52012 | 0.38 |
| GO:0051274 | P | beta-glucan biosynthetic process | 46 | 17430 | 148 | 52012 | 0.7 |
| GO:0072348 | P | sulfur compound transport | 19 | 17430 | 43 | 52012 | 0.19 |
| GO:0019693 | P | ribose phosphate metabolic process | 159 | 17430 | 643 | 52012 | 1 |
| GO:0006066 | P | alcohol metabolic process | 17 | 17430 | 109 | 52012 | 1 |
| GO:0006796 | P | phosphate-containing compound metabolic process | 1895 | 17430 | 5379 | 52012 | 0.025 |
| GO:0006790 | P | sulfur compound metabolic process | 39 | 17430 | 135 | 52012 | 0.82 |
| GO:0006793 | P | phosphorus metabolic process | 1901 | 17430 | 5392 | 52012 | 0.023 |
| GO:0006417 | P | regulation of translation | 15 | 17430 | 40 | 52012 | 0.4 |
| GO:0006415 | P | translational termination | 22 | 17430 | 54 | 52012 | 0.26 |
| GO:0006414 | P | translational elongation | 24 | 17430 | 138 | 52012 | 1 |
| GO:0006413 | P | translational initiation | 36 | 17430 | 124 | 52012 | 0.8 |
| GO:0006412 | P | translation | 637 | 17430 | 1731 | 52012 | 0.022 |
| GO:0048523 | P | negative regulation of cellular process | 32 | 17430 | 70 | 52012 | 0.091 |
| GO:0019362 | P | pyridine nucleotide metabolic process | 68 | 17430 | 262 | 52012 | 0.98 |
| GO:0034440 | P | lipid oxidation | 11 | 17430 | 21 | 52012 | 0.16 |
| GO:0008104 | P | protein localization | 258 | 17430 | 1369 | 52012 | 1 |
| GO:0009311 | P | oligosaccharide metabolic process | 54 | 17430 | 163 | 52012 | 0.56 |
| GO:0043603 | P | cellular amide metabolic process | 664 | 17430 | 1839 | 52012 | 0.05 |
| GO:0009451 | P | RNA modification | 46 | 17430 | 127 | 52012 | 0.35 |
| GO:0006739 | P | NADP metabolic process | 15 | 17430 | 54 | 52012 | 0.78 |
| GO:0007165 | P | signal transduction | 366 | 17430 | 1348 | 52012 | 1 |
| GO:0007006 | P | mitochondrial membrane organization | 6 | 17430 | 35 | 52012 | 0.96 |
| GO:0007005 | P | mitochondrion organization | 21 | 17430 | 53 | 52012 | 0.3 |
| GO:0044712 | P | single-organism catabolic process | 105 | 17430 | 435 | 52012 | 1 |
| GO:0042445 | P | hormone metabolic process | 12 | 17430 | 22 | 52012 | 0.12 |
| GO:0044710 | P | single-organism metabolic process | 2510 | 17430 | 7815 | 52012 | 0.98 |
| GO:0044711 | P | single-organism biosynthetic process | 559 | 17430 | 2083 | 52012 | 1 |
| GO:0015937 | P | coenzyme A biosynthetic process | 5 | 17430 | 19 | 52012 | 0.76 |
| GO:0006732 | P | coenzyme metabolic process | 129 | 17430 | 473 | 52012 | 0.98 |
| GO:0042455 | P | ribonucleoside biosynthetic process | 56 | 17430 | 250 | 52012 | 1 |
| GO:0009314 | P | response to radiation | 11 | 17430 | 115 | 52012 | 1 |
| GO:0006661 | P | phosphatidylinositol biosynthetic process | 27 | 17430 | 64 | 52012 | 0.19 |
| GO:0006662 | P | glycerol ether metabolic process | 42 | 17430 | 133 | 52012 | 0.66 |
| GO:0009767 | P | photosynthetic electron transport chain | 16 | 17430 | 41 | 52012 | 0.35 |
| GO:0006664 | P | glycolipid metabolic process | 31 | 17430 | 120 | 52012 | 0.92 |
| GO:0006665 | P | sphingolipid metabolic process | 9 | 17430 | 25 | 52012 | 0.49 |
| GO:0051056 | P | regulation of small GTPase mediated signal transduction | 12 | 17430 | 37 | 52012 | 0.59 |
| GO:0046039 | P | GTP metabolic process | 8 | 17430 | 23 | 52012 | 0.53 |
| GO:0046034 | P | ATP metabolic process | 110 | 17430 | 465 | 52012 | 1 |
| GO:0033365 | P | protein localization to organelle | 56 | 17430 | 147 | 52012 | 0.23 |
| GO:0046036 | P | CTP metabolic process | 5 | 17430 | 10 | 52012 | 0.32 |
| GO:0046031 | P | ADP metabolic process | 50 | 17430 | 187 | 52012 | 0.94 |
| GO:0000394 | P | RNA splicing, via endonucleolytic cleavage and ligation | 12 | 17430 | 28 | 52012 | 0.29 |
| GO:0048278 | P | vesicle docking | 14 | 17430 | 45 | 52012 | 0.64 |
| GO:0023052 | P | signaling | 366 | 17430 | 1348 | 52012 | 1 |
| GO:0010035 | P | response to inorganic substance | 10 | 17430 | 67 | 52012 | 1 |
| GO:0009179 | P | purine ribonucleoside diphosphate metabolic process | 50 | 17430 | 187 | 52012 | 0.94 |
| GO:0009072 | P | aromatic amino acid family metabolic process | 35 | 17430 | 135 | 52012 | 0.93 |
| GO:0016192 | P | vesicle-mediated transport | 199 | 17430 | 682 | 52012 | 0.96 |
| GO:0018205 | P | peptidyl-lysine modification | 30 | 17430 | 68 | 52012 | 0.13 |
| GO:1901072 | P | glucosamine-containing compound catabolic process | 15 | 17430 | 37 | 52012 | 0.31 |
| GO:0016116 | P | carotenoid metabolic process | 5 | 17430 | 23 | 52012 | 0.87 |
| GO:1901070 | P | guanosine-containing compound biosynthetic process | 7 | 17430 | 12 | 52012 | 0.18 |
| GO:1904029 | P | regulation of cyclin-dependent protein kinase activity | 12 | 17430 | 130 | 52012 | 1 |
| GO:0044784 | P | metaphase/anaphase transition of cell cycle | 7 | 17430 | 7 | 52012 | 0.039 |
| GO:0016569 | P | covalent chromatin modification | 32 | 17430 | 77 | 52012 | 0.18 |
| GO:0018022 | P | peptidyl-lysine methylation | 17 | 17430 | 42 | 52012 | 0.3 |
| GO:0015914 | P | phospholipid transport | 18 | 17430 | 43 | 52012 | 0.25 |
| GO:0006298 | P | mismatch repair | 15 | 17430 | 44 | 52012 | 0.53 |
| GO:0046493 | P | lipid A metabolic process | 8 | 17430 | 25 | 52012 | 0.61 |
| GO:0010629 | P | negative regulation of gene expression | 26 | 17430 | 55 | 52012 | 0.095 |
| GO:0006807 | P | nitrogen compound metabolic process | 3039 | 17430 | 9325 | 52012 | 0.93 |
| GO:0046496 | P | nicotinamide nucleotide metabolic process | 68 | 17430 | 262 | 52012 | 0.98 |
| GO:0034660 | P | ncRNA metabolic process | 161 | 17430 | 480 | 52012 | 0.51 |
| GO:0009772 | P | photosynthetic electron transport in photosystem II | 9 | 17430 | 14 | 52012 | 0.098 |
| GO:0051187 | P | cofactor catabolic process | 5 | 17430 | 13 | 52012 | 0.49 |
| GO:0051186 | P | cofactor metabolic process | 163 | 17430 | 606 | 52012 | 1 |
| GO:0046916 | P | cellular transition metal ion homeostasis | 5 | 17430 | 18 | 52012 | 0.72 |
| GO:0051189 | P | prosthetic group metabolic process | 7 | 17430 | 37 | 52012 | 0.95 |
| GO:0051188 | P | cofactor biosynthetic process | 87 | 17430 | 331 | 52012 | 0.98 |
| GO:0006568 | P | tryptophan metabolic process | 9 | 17430 | 30 | 52012 | 0.67 |
| GO:0070271 | P | protein complex biogenesis | 83 | 17430 | 415 | 52012 | 1 |
| GO:0006364 | P | rRNA processing | 24 | 17430 | 64 | 52012 | 0.36 |
| GO:0006367 | P | transcription initiation from RNA polymerase II promoter | 14 | 17430 | 68 | 52012 | 0.97 |
| GO:0006366 | P | transcription from RNA polymerase II promoter | 45 | 17430 | 163 | 52012 | 0.89 |
| GO:0006368 | P | transcription elongation from RNA polymerase II promoter | 7 | 17430 | 13 | 52012 | 0.22 |
| GO:0007186 | P | G-protein coupled receptor signaling pathway | 26 | 17430 | 111 | 52012 | 0.96 |
| GO:0046051 | P | UTP metabolic process | 5 | 17430 | 10 | 52012 | 0.32 |
| GO:0000723 | P | telomere maintenance | 6 | 17430 | 8 | 52012 | 0.11 |
| GO:0019748 | P | secondary metabolic process | 5 | 17430 | 78 | 52012 | 1 |
| GO:0019310 | P | inositol catabolic process | 5 | 17430 | 21 | 52012 | 0.82 |
| GO:0006420 | P | arginyl-tRNA aminoacylation | 6 | 17430 | 27 | 52012 | 0.87 |
| GO:0000271 | P | polysaccharide biosynthetic process | 48 | 17430 | 225 | 52012 | 1 |
| GO:0050790 | P | regulation of catalytic activity | 31 | 17430 | 237 | 52012 | 1 |
| GO:0019319 | P | hexose biosynthetic process | 8 | 17430 | 40 | 52012 | 0.94 |
| GO:0050794 | P | regulation of cellular process | 1791 | 17430 | 5061 | 52012 | 0.019 |
| GO:0019637 | P | organophosphate metabolic process | 314 | 17430 | 1292 | 52012 | 1 |
| GO:0031163 | P | metallo-sulfur cluster assembly | 12 | 17430 | 22 | 52012 | 0.12 |
| GO:0070972 | P | protein localization to endoplasmic reticulum | 25 | 17430 | 59 | 52012 | 0.19 |
| GO:0051235 | P | maintenance of location | 11 | 17430 | 34 | 52012 | 0.6 |
| GO:0051234 | P | establishment of localization | 1511 | 17430 | 5020 | 52012 | 1 |
| GO:0090407 | P | organophosphate biosynthetic process | 158 | 17430 | 584 | 52012 | 0.99 |
| GO:0046148 | P | pigment biosynthetic process | 17 | 17430 | 107 | 52012 | 1 |
| GO:0015985 | P | energy coupled proton transport, down electrochemical gradient | 40 | 17430 | 88 | 52012 | 0.069 |
| GO:0051174 | P | regulation of phosphorus metabolic process | 26 | 17430 | 151 | 52012 | 1 |
| GO:0015986 | P | ATP synthesis coupled proton transport | 40 | 17430 | 88 | 52012 | 0.069 |
| GO:0015980 | P | energy derivation by oxidation of organic compounds | 26 | 17430 | 153 | 52012 | 1 |
| GO:0034220 | P | ion transmembrane transport | 139 | 17430 | 410 | 52012 | 0.47 |
| GO:0046394 | P | carboxylic acid biosynthetic process | 142 | 17430 | 659 | 52012 | 1 |
| GO:0046395 | P | carboxylic acid catabolic process | 21 | 17430 | 87 | 52012 | 0.93 |
| GO:0050896 | P | response to stimulus | 1043 | 17430 | 3207 | 52012 | 0.81 |
| GO:0046390 | P | ribose phosphate biosynthetic process | 63 | 17430 | 280 | 52012 | 1 |
| GO:0015988 | P | energy coupled proton transmembrane transport, against electrochemical gradient | 51 | 17430 | 146 | 52012 | 0.43 |
| GO:0051338 | P | regulation of transferase activity | 24 | 17430 | 130 | 52012 | 1 |
| GO:0033865 | P | nucleoside bisphosphate metabolic process | 20 | 17430 | 51 | 52012 | 0.32 |
| GO:0006102 | P | isocitrate metabolic process | 5 | 17430 | 27 | 52012 | 0.93 |
| GO:0051170 | P | nuclear import | 11 | 17430 | 27 | 52012 | 0.35 |
| GO:0006518 | P | peptide metabolic process | 658 | 17430 | 1802 | 52012 | 0.03 |
| GO:0032956 | P | regulation of actin cytoskeleton organization | 20 | 17430 | 47 | 52012 | 0.22 |
| GO:0006633 | P | fatty acid biosynthetic process | 75 | 17430 | 261 | 52012 | 0.89 |
| GO:0033045 | P | regulation of sister chromatid segregation | 7 | 17430 | 7 | 52012 | 0.039 |
| GO:0006631 | P | fatty acid metabolic process | 104 | 17430 | 307 | 52012 | 0.48 |
| GO:0033047 | P | regulation of mitotic sister chromatid segregation | 7 | 17430 | 7 | 52012 | 0.039 |
| GO:0006635 | P | fatty acid beta-oxidation | 9 | 17430 | 21 | 52012 | 0.33 |
| GO:0033043 | P | regulation of organelle organization | 28 | 17430 | 54 | 52012 | 0.042 |
| GO:0072663 | P | establishment of protein localization to peroxisome | 6 | 17430 | 22 | 52012 | 0.74 |
| GO:0072662 | P | protein localization to peroxisome | 6 | 17430 | 22 | 52012 | 0.74 |
| GO:0051649 | P | establishment of localization in cell | 237 | 17430 | 1069 | 52012 | 1 |
| GO:0045901 | P | positive regulation of translational elongation | 10 | 17430 | 25 | 52012 | 0.38 |
| GO:0009312 | P | oligosaccharide biosynthetic process | 36 | 17430 | 122 | 52012 | 0.78 |
| GO:0042401 | P | cellular biogenic amine biosynthetic process | 16 | 17430 | 30 | 52012 | 0.092 |
| GO:0009250 | P | glucan biosynthetic process | 46 | 17430 | 208 | 52012 | 1 |
| GO:0070887 | P | cellular response to chemical stimulus | 9 | 17430 | 16 | 52012 | 0.15 |
| GO:0006457 | P | protein folding | 117 | 17430 | 705 | 52012 | 1 |
| GO:0009259 | P | ribonucleotide metabolic process | 149 | 17430 | 589 | 52012 | 1 |
| GO:0044699 | P | single-organism process | 3863 | 17430 | 11479 | 52012 | 0.4 |
| GO:0071806 | P | protein transmembrane transport | 19 | 17430 | 43 | 52012 | 0.19 |
| GO:0071804 | P | cellular potassium ion transport | 25 | 17430 | 94 | 52012 | 0.87 |
| GO:0071805 | P | potassium ion transmembrane transport | 25 | 17430 | 94 | 52012 | 0.87 |
| GO:0051246 | P | regulation of protein metabolic process | 40 | 17430 | 210 | 52012 | 1 |
| GO:0009057 | P | macromolecule catabolic process | 161 | 17430 | 664 | 52012 | 1 |
| GO:0051640 | P | organelle localization | 5 | 17430 | 9 | 52012 | 0.26 |
| GO:0031399 | P | regulation of protein modification process | 22 | 17430 | 151 | 52012 | 1 |
| GO:0006030 | P | chitin metabolic process | 15 | 17430 | 37 | 52012 | 0.31 |
| GO:0006032 | P | chitin catabolic process | 15 | 17430 | 37 | 52012 | 0.31 |
| GO:1901700 | P | response to oxygen-containing compound | 19 | 17430 | 29 | 52012 | 0.019 |
| GO:0042168 | P | heme metabolic process | 11 | 17430 | 37 | 52012 | 0.69 |
| GO:0072599 | P | establishment of protein localization to endoplasmic reticulum | 14 | 17430 | 32 | 52012 | 0.25 |
| GO:1902593 | P | single-organism nuclear import | 11 | 17430 | 27 | 52012 | 0.35 |
| GO:0072595 | P | maintenance of protein localization in organelle | 11 | 17430 | 27 | 52012 | 0.35 |
| GO:0072594 | P | establishment of protein localization to organelle | 44 | 17430 | 120 | 52012 | 0.33 |
| GO:0072593 | P | reactive oxygen species metabolic process | 10 | 17430 | 52 | 52012 | 0.97 |
| GO:0034248 | P | regulation of cellular amide metabolic process | 15 | 17430 | 40 | 52012 | 0.4 |
| GO:0006928 | P | movement of cell or subcellular component | 92 | 17430 | 328 | 52012 | 0.94 |
| GO:0043574 | P | peroxisomal transport | 6 | 17430 | 22 | 52012 | 0.74 |
| GO:0007031 | P | peroxisome organization | 14 | 17430 | 41 | 52012 | 0.53 |
| GO:0070838 | P | divalent metal ion transport | 14 | 17430 | 74 | 52012 | 0.99 |
| GO:0045185 | P | maintenance of protein location | 11 | 17430 | 34 | 52012 | 0.6 |
| GO:1901137 | P | carbohydrate derivative biosynthetic process | 171 | 17430 | 617 | 52012 | 0.99 |
| GO:1901136 | P | carbohydrate derivative catabolic process | 24 | 17430 | 80 | 52012 | 0.72 |
| GO:1901135 | P | carbohydrate derivative metabolic process | 322 | 17430 | 1166 | 52012 | 1 |
| GO:0042493 | P | response to drug | 63 | 17430 | 165 | 52012 | 0.21 |
| GO:0033692 | P | cellular polysaccharide biosynthetic process | 48 | 17430 | 225 | 52012 | 1 |
| GO:0009089 | P | lysine biosynthetic process via diaminopimelate | 5 | 17430 | 28 | 52012 | 0.94 |
| GO:0090150 | P | establishment of protein localization to membrane | 32 | 17430 | 88 | 52012 | 0.38 |
| GO:0009085 | P | lysine biosynthetic process | 5 | 17430 | 28 | 52012 | 0.94 |
| GO:0009084 | P | glutamine family amino acid biosynthetic process | 13 | 17430 | 66 | 52012 | 0.98 |
| GO:0009086 | P | methionine biosynthetic process | 8 | 17430 | 50 | 52012 | 0.99 |
| GO:0042157 | P | lipoprotein metabolic process | 22 | 17430 | 71 | 52012 | 0.66 |
| GO:0030243 | P | cellulose metabolic process | 30 | 17430 | 110 | 52012 | 0.87 |
| GO:0046364 | P | monosaccharide biosynthetic process | 8 | 17430 | 40 | 52012 | 0.94 |
| GO:0030244 | P | cellulose biosynthetic process | 30 | 17430 | 108 | 52012 | 0.84 |
| GO:0072528 | P | pyrimidine-containing compound biosynthetic process | 28 | 17430 | 95 | 52012 | 0.76 |
| GO:0009108 | P | coenzyme biosynthetic process | 55 | 17430 | 203 | 52012 | 0.93 |
| GO:0034622 | P | cellular macromolecular complex assembly | 82 | 17430 | 415 | 52012 | 1 |
| GO:0008033 | P | tRNA processing | 65 | 17430 | 170 | 52012 | 0.2 |
| GO:0072522 | P | purine-containing compound biosynthetic process | 70 | 17430 | 330 | 52012 | 1 |
| GO:0009100 | P | glycoprotein metabolic process | 68 | 17430 | 179 | 52012 | 0.21 |
| GO:0009101 | P | glycoprotein biosynthetic process | 68 | 17430 | 179 | 52012 | 0.21 |
| GO:0072527 | P | pyrimidine-containing compound metabolic process | 32 | 17430 | 108 | 52012 | 0.76 |
| GO:0006325 | P | chromatin organization | 74 | 17430 | 267 | 52012 | 0.94 |
| GO:0006323 | P | DNA packaging | 32 | 17430 | 165 | 52012 | 1 |
| GO:0072330 | P | monocarboxylic acid biosynthetic process | 78 | 17430 | 272 | 52012 | 0.9 |
| GO:0065004 | P | protein-DNA complex assembly | 30 | 17430 | 163 | 52012 | 1 |
| GO:0046164 | P | alcohol catabolic process | 5 | 17430 | 21 | 52012 | 0.82 |
| GO:0045934 | P | negative regulation of nucleobase-containing compound metabolic process | 7 | 17430 | 37 | 52012 | 0.95 |
| GO:0030029 | P | actin filament-based process | 47 | 17430 | 138 | 52012 | 0.49 |
| GO:0006418 | P | tRNA aminoacylation for protein translation | 70 | 17430 | 235 | 52012 | 0.82 |
| GO:0031123 | P | RNA 3'-end processing | 15 | 17430 | 37 | 52012 | 0.31 |
| GO:0010817 | P | regulation of hormone levels | 15 | 17430 | 22 | 52012 | 0.028 |
| GO:0043628 | P | ncRNA 3'-end processing | 5 | 17430 | 5 | 52012 | 0.079 |
| GO:0006888 | P | ER to Golgi vesicle-mediated transport | 37 | 17430 | 106 | 52012 | 0.45 |
| GO:0007265 | P | Ras protein signal transduction | 12 | 17430 | 33 | 52012 | 0.46 |
| GO:0043623 | P | cellular protein complex assembly | 45 | 17430 | 238 | 52012 | 1 |
| GO:0034754 | P | cellular hormone metabolic process | 12 | 17430 | 22 | 52012 | 0.12 |
| GO:0008610 | P | lipid biosynthetic process | 241 | 17430 | 628 | 52012 | 0.04 |
| GO:0006497 | P | protein lipidation | 22 | 17430 | 71 | 52012 | 0.66 |
| GO:0044238 | P | primary metabolic process | 6117 | 17430 | 18025 | 52012 | 0.15 |
| GO:0005975 | P | carbohydrate metabolic process | 629 | 17430 | 2274 | 52012 | 1 |
| GO:0006558 | P | L-phenylalanine metabolic process | 7 | 17430 | 34 | 52012 | 0.92 |
| GO:0005976 | P | polysaccharide metabolic process | 99 | 17430 | 346 | 52012 | 0.93 |
| GO:0006556 | P | S-adenosylmethionine biosynthetic process | 8 | 17430 | 18 | 52012 | 0.32 |
| GO:0009620 | P | response to fungus | 5 | 17430 | 7 | 52012 | 0.16 |
| GO:0006555 | P | methionine metabolic process | 10 | 17430 | 60 | 52012 | 0.99 |
| GO:0032200 | P | telomere organization | 6 | 17430 | 8 | 52012 | 0.11 |
| GO:0044237 | P | cellular metabolic process | 5614 | 17430 | 16458 | 52012 | 0.084 |
| GO:0055072 | P | iron ion homeostasis | 5 | 17430 | 17 | 52012 | 0.68 |
| GO:0006399 | P | tRNA metabolic process | 135 | 17430 | 403 | 52012 | 0.52 |
| GO:0008299 | P | isoprenoid biosynthetic process | 47 | 17430 | 137 | 52012 | 0.47 |
| GO:0019363 | P | pyridine nucleotide biosynthetic process | 8 | 17430 | 21 | 52012 | 0.45 |
| GO:0006259 | P | DNA metabolic process | 239 | 17430 | 795 | 52012 | 0.94 |
| GO:0045892 | P | negative regulation of transcription, DNA-templated | 5 | 17430 | 36 | 52012 | 0.99 |
| GO:0048522 | P | positive regulation of cellular process | 43 | 17430 | 88 | 52012 | 0.029 |
| GO:0008295 | P | spermidine biosynthetic process | 10 | 17430 | 11 | 52012 | 0.021 |
| GO:0006397 | P | mRNA processing | 64 | 17430 | 151 | 52012 | 0.068 |
| GO:0006479 | P | protein methylation | 34 | 17430 | 71 | 52012 | 0.057 |
| GO:0019220 | P | regulation of phosphate metabolic process | 26 | 17430 | 151 | 52012 | 1 |
| GO:0060249 | P | anatomical structure homeostasis | 6 | 17430 | 8 | 52012 | 0.11 |
| GO:0009218 | P | pyrimidine ribonucleotide metabolic process | 5 | 17430 | 15 | 52012 | 0.59 |
| GO:0048583 | P | regulation of response to stimulus | 25 | 17430 | 76 | 52012 | 0.57 |
| GO:0006778 | P | porphyrin-containing compound metabolic process | 21 | 17430 | 105 | 52012 | 0.99 |
| GO:0006779 | P | porphyrin-containing compound biosynthetic process | 16 | 17430 | 92 | 52012 | 1 |
| GO:0046134 | P | pyrimidine nucleoside biosynthetic process | 5 | 17430 | 15 | 52012 | 0.59 |
| GO:0010965 | P | regulation of mitotic sister chromatid separation | 7 | 17430 | 7 | 52012 | 0.039 |
| GO:0008643 | P | carbohydrate transport | 13 | 17430 | 48 | 52012 | 0.79 |
| GO:1901362 | P | organic cyclic compound biosynthetic process | 1598 | 17430 | 4573 | 52012 | 0.068 |
| GO:0046131 | P | pyrimidine ribonucleoside metabolic process | 8 | 17430 | 22 | 52012 | 0.49 |
| GO:1901360 | P | organic cyclic compound metabolic process | 2315 | 17430 | 6972 | 52012 | 0.66 |
| GO:1901361 | P | organic cyclic compound catabolic process | 32 | 17430 | 152 | 52012 | 1 |
| GO:0051205 | P | protein insertion into membrane | 11 | 17430 | 21 | 52012 | 0.16 |
| GO:0006122 | P | mitochondrial electron transport, ubiquinol to cytochrome c | 7 | 17430 | 20 | 52012 | 0.53 |
| GO:0016998 | P | cell wall macromolecule catabolic process | 17 | 17430 | 59 | 52012 | 0.75 |
| GO:0009966 | P | regulation of signal transduction | 18 | 17430 | 71 | 52012 | 0.88 |
| GO:0006787 | P | porphyrin-containing compound catabolic process | 5 | 17430 | 13 | 52012 | 0.49 |
| GO:0044275 | P | cellular carbohydrate catabolic process | 5 | 17430 | 23 | 52012 | 0.87 |
| GO:0006075 | P | (1->3)-beta-D-glucan biosynthetic process | 16 | 17430 | 40 | 52012 | 0.32 |
| GO:0006074 | P | (1->3)-beta-D-glucan metabolic process | 16 | 17430 | 40 | 52012 | 0.32 |
| GO:0022900 | P | electron transport chain | 35 | 17430 | 120 | 52012 | 0.79 |
| GO:0055086 | P | nucleobase-containing small molecule metabolic process | 224 | 17430 | 897 | 52012 | 1 |
| GO:0006073 | P | cellular glucan metabolic process | 83 | 17430 | 280 | 52012 | 0.85 |
| GO:0022904 | P | respiratory electron transport chain | 13 | 17430 | 62 | 52012 | 0.96 |
| GO:0019359 | P | nicotinamide nucleotide biosynthetic process | 8 | 17430 | 21 | 52012 | 0.45 |
| GO:0034470 | P | ncRNA processing | 91 | 17430 | 225 | 52012 | 0.074 |
| GO:0048017 | P | inositol lipid-mediated signaling | 5 | 17430 | 12 | 52012 | 0.43 |
| GO:0006606 | P | protein import into nucleus | 11 | 17430 | 27 | 52012 | 0.35 |
| GO:0006605 | P | protein targeting | 50 | 17430 | 140 | 52012 | 0.38 |
| GO:0045184 | P | establishment of protein localization | 247 | 17430 | 1323 | 52012 | 1 |
| GO:0072657 | P | protein localization to membrane | 32 | 17430 | 88 | 52012 | 0.38 |
| GO:0072655 | P | establishment of protein localization to mitochondrion | 11 | 17430 | 36 | 52012 | 0.66 |
| GO:0043436 | P | oxoacid metabolic process | 404 | 17430 | 1570 | 52012 | 1 |
| GO:0042451 | P | purine nucleoside biosynthetic process | 54 | 17430 | 242 | 52012 | 1 |
| GO:1902099 | P | regulation of metaphase/anaphase transition of cell cycle | 7 | 17430 | 7 | 52012 | 0.039 |
| GO:0044700 | P | single organism signaling | 366 | 17430 | 1348 | 52012 | 1 |
| GO:1901564 | P | organonitrogen compound metabolic process | 1143 | 17430 | 3938 | 52012 | 1 |
| GO:1901565 | P | organonitrogen compound catabolic process | 41 | 17430 | 147 | 52012 | 0.87 |
| GO:1901566 | P | organonitrogen compound biosynthetic process | 903 | 17430 | 2975 | 52012 | 1 |
| GO:0055076 | P | transition metal ion homeostasis | 5 | 17430 | 18 | 52012 | 0.72 |
| GO:0016054 | P | organic acid catabolic process | 26 | 17430 | 87 | 52012 | 0.73 |
| GO:0016052 | P | carbohydrate catabolic process | 72 | 17430 | 267 | 52012 | 0.96 |
| GO:0016053 | P | organic acid biosynthetic process | 165 | 17430 | 773 | 52012 | 1 |
| GO:0016050 | P | vesicle organization | 5 | 17430 | 10 | 52012 | 0.32 |
| GO:0016051 | P | carbohydrate biosynthetic process | 94 | 17430 | 396 | 52012 | 1 |
| GO:0002376 | P | immune system process | 10 | 17430 | 19 | 52012 | 0.17 |
| GO:0007154 | P | cell communication | 451 | 17430 | 1521 | 52012 | 0.99 |
| GO:0015977 | P | carbon fixation | 9 | 17430 | 16 | 52012 | 0.15 |
| GO:0033554 | P | cellular response to stress | 168 | 17430 | 490 | 52012 | 0.41 |
| GO:0006511 | P | ubiquitin-dependent protein catabolic process | 86 | 17430 | 369 | 52012 | 1 |
| GO:0006119 | P | oxidative phosphorylation | 9 | 17430 | 46 | 52012 | 0.96 |
| GO:0006672 | P | ceramide metabolic process | 5 | 17430 | 24 | 52012 | 0.89 |
| GO:0032535 | P | regulation of cellular component size | 21 | 17430 | 49 | 52012 | 0.21 |
| GO:0009148 | P | pyrimidine nucleoside triphosphate biosynthetic process | 5 | 17430 | 10 | 52012 | 0.32 |
| GO:0022607 | P | cellular component assembly | 147 | 17430 | 463 | 52012 | 0.73 |
| GO:0042398 | P | cellular modified amino acid biosynthetic process | 12 | 17430 | 81 | 52012 | 1 |
| GO:0009141 | P | nucleoside triphosphate metabolic process | 119 | 17430 | 489 | 52012 | 1 |
| GO:0009142 | P | nucleoside triphosphate biosynthetic process | 47 | 17430 | 211 | 52012 | 1 |
| GO:0006825 | P | copper ion transport | 12 | 17430 | 26 | 52012 | 0.23 |
| GO:0009144 | P | purine nucleoside triphosphate metabolic process | 118 | 17430 | 488 | 52012 | 1 |
| GO:0009145 | P | purine nucleoside triphosphate biosynthetic process | 47 | 17430 | 211 | 52012 | 1 |
| GO:0009147 | P | pyrimidine nucleoside triphosphate metabolic process | 6 | 17430 | 11 | 52012 | 0.24 |
| GO:0009063 | P | cellular amino acid catabolic process | 12 | 17430 | 66 | 52012 | 0.99 |
| GO:0009062 | P | fatty acid catabolic process | 9 | 17430 | 21 | 52012 | 0.33 |
| GO:0009060 | P | aerobic respiration | 13 | 17430 | 65 | 52012 | 0.97 |
| GO:0009067 | P | aspartate family amino acid biosynthetic process | 21 | 17430 | 104 | 52012 | 0.99 |
| GO:0009066 | P | aspartate family amino acid metabolic process | 23 | 17430 | 115 | 52012 | 0.99 |
| GO:0009065 | P | glutamine family amino acid catabolic process | 6 | 17430 | 14 | 52012 | 0.39 |
| GO:0009064 | P | glutamine family amino acid metabolic process | 31 | 17430 | 92 | 52012 | 0.52 |
| GO:0042158 | P | lipoprotein biosynthetic process | 22 | 17430 | 71 | 52012 | 0.66 |
| GO:0090662 | P | ATP hydrolysis coupled transmembrane transport | 51 | 17430 | 146 | 52012 | 0.43 |
| GO:0044271 | P | cellular nitrogen compound biosynthetic process | 2167 | 17430 | 6174 | 52012 | 0.025 |
| GO:0043545 | P | molybdopterin cofactor metabolic process | 7 | 17430 | 37 | 52012 | 0.95 |
| GO:0042440 | P | pigment metabolic process | 22 | 17430 | 120 | 52012 | 1 |
| GO:0043549 | P | regulation of kinase activity | 24 | 17430 | 130 | 52012 | 1 |
| GO:0090066 | P | regulation of anatomical structure size | 21 | 17430 | 49 | 52012 | 0.21 |
| GO:1901068 | P | guanosine-containing compound metabolic process | 16 | 17430 | 45 | 52012 | 0.47 |
| GO:0016571 | P | histone methylation | 17 | 17430 | 42 | 52012 | 0.3 |
| GO:0016570 | P | histone modification | 32 | 17430 | 77 | 52012 | 0.18 |
| GO:0045727 | P | positive regulation of translation | 10 | 17430 | 25 | 52012 | 0.38 |
| GO:0071669 | P | plant-type cell wall organization or biogenesis | 20 | 17430 | 64 | 52012 | 0.65 |
| GO:0006818 | P | hydrogen transport | 110 | 17430 | 270 | 52012 | 0.049 |
| GO:0006813 | P | potassium ion transport | 29 | 17430 | 149 | 52012 | 1 |
| GO:0006812 | P | cation transport | 344 | 17430 | 1083 | 52012 | 0.82 |
| GO:0006811 | P | ion transport | 445 | 17430 | 1293 | 52012 | 0.32 |
| GO:0006810 | P | transport | 1500 | 17430 | 4999 | 52012 | 1 |
| GO:0046700 | P | heterocycle catabolic process | 32 | 17430 | 86 | 52012 | 0.34 |
| GO:0006952 | P | defense response | 76 | 17430 | 473 | 52012 | 1 |
| GO:0012501 | P | programmed cell death | 5 | 17430 | 8 | 52012 | 0.21 |
| GO:0006950 | P | response to stress | 452 | 17430 | 1440 | 52012 | 0.9 |
| GO:0006189 | P | 'de novo' IMP biosynthetic process | 5 | 17430 | 22 | 52012 | 0.84 |
| GO:0006188 | P | IMP biosynthetic process | 9 | 17430 | 43 | 52012 | 0.93 |
| GO:0006955 | P | immune response | 10 | 17430 | 19 | 52012 | 0.17 |
| GO:0034654 | P | nucleobase-containing compound biosynthetic process | 1451 | 17430 | 4164 | 52012 | 0.094 |
| GO:0034655 | P | nucleobase-containing compound catabolic process | 22 | 17430 | 65 | 52012 | 0.53 |
| GO:0006814 | P | sodium ion transport | 7 | 17430 | 75 | 52012 | 1 |
| GO:0048015 | P | phosphatidylinositol-mediated signaling | 5 | 17430 | 12 | 52012 | 0.43 |
| GO:0006183 | P | GTP biosynthetic process | 5 | 17430 | 10 | 52012 | 0.32 |
| GO:0043094 | P | cellular metabolic compound salvage | 6 | 17430 | 59 | 52012 | 1 |
| GO:0051603 | P | proteolysis involved in cellular protein catabolic process | 104 | 17430 | 427 | 52012 | 1 |
| GO:0046903 | P | secretion | 39 | 17430 | 83 | 52012 | 0.053 |
| GO:0051606 | P | detection of stimulus | 5 | 17430 | 14 | 52012 | 0.54 |
| GO:0044270 | P | cellular nitrogen compound catabolic process | 33 | 17430 | 86 | 52012 | 0.28 |
| GO:0046907 | P | intracellular transport | 237 | 17430 | 1069 | 52012 | 1 |
| GO:0044272 | P | sulfur compound biosynthetic process | 20 | 17430 | 50 | 52012 | 0.29 |
| GO:0006213 | P | pyrimidine nucleoside metabolic process | 8 | 17430 | 22 | 52012 | 0.49 |
| GO:0006354 | P | DNA-templated transcription, elongation | 9 | 17430 | 29 | 52012 | 0.64 |
| GO:0006357 | P | regulation of transcription from RNA polymerase II promoter | 24 | 17430 | 83 | 52012 | 0.77 |
| GO:0006352 | P | DNA-templated transcription, initiation | 42 | 17430 | 177 | 52012 | 0.98 |
| GO:0006544 | P | glycine metabolic process | 7 | 17430 | 84 | 52012 | 1 |
| GO:0045333 | P | cellular respiration | 26 | 17430 | 125 | 52012 | 0.99 |
| GO:1901616 | P | organic hydroxy compound catabolic process | 5 | 17430 | 21 | 52012 | 0.82 |
| GO:1901617 | P | organic hydroxy compound biosynthetic process | 6 | 17430 | 15 | 52012 | 0.44 |
| GO:1901615 | P | organic hydroxy compound metabolic process | 21 | 17430 | 115 | 52012 | 1 |
| GO:0006433 | P | prolyl-tRNA aminoacylation | 6 | 17430 | 19 | 52012 | 0.63 |
| GO:0035437 | P | maintenance of protein localization in endoplasmic reticulum | 11 | 17430 | 27 | 52012 | 0.35 |
| GO:0043043 | P | peptide biosynthetic process | 640 | 17430 | 1750 | 52012 | 0.029 |
| GO:0035434 | P | copper ion transmembrane transport | 10 | 17430 | 12 | 52012 | 0.03 |
| GO:0046174 | P | polyol catabolic process | 5 | 17430 | 21 | 52012 | 0.82 |
| GO:0000272 | P | polysaccharide catabolic process | 14 | 17430 | 51 | 52012 | 0.79 |
| GO:0006139 | P | nucleobase-containing compound metabolic process | 2155 | 17430 | 6446 | 52012 | 0.55 |
| GO:1902531 | P | regulation of intracellular signal transduction | 12 | 17430 | 37 | 52012 | 0.59 |
| GO:0051321 | P | meiotic cell cycle | 5 | 17430 | 8 | 52012 | 0.21 |
| GO:0043161 | P | proteasome-mediated ubiquitin-dependent protein catabolic process | 8 | 17430 | 34 | 52012 | 0.86 |
| GO:0042278 | P | purine nucleoside metabolic process | 140 | 17430 | 551 | 52012 | 1 |
| GO:0033875 | P | ribonucleoside bisphosphate metabolic process | 20 | 17430 | 51 | 52012 | 0.32 |
| GO:0005985 | P | sucrose metabolic process | 15 | 17430 | 55 | 52012 | 0.8 |
| GO:0043933 | P | macromolecular complex subunit organization | 183 | 17430 | 587 | 52012 | 0.82 |
| GO:0034968 | P | histone lysine methylation | 17 | 17430 | 42 | 52012 | 0.3 |
| GO:0046836 | P | glycolipid transport | 6 | 17430 | 14 | 52012 | 0.39 |
| GO:0032271 | P | regulation of protein polymerization | 20 | 17430 | 47 | 52012 | 0.22 |
| GO:0006506 | P | GPI anchor biosynthetic process | 18 | 17430 | 64 | 52012 | 0.78 |
| GO:0006508 | P | proteolysis | 502 | 17430 | 1664 | 52012 | 0.98 |
| GO:0046351 | P | disaccharide biosynthetic process | 26 | 17430 | 93 | 52012 | 0.82 |
| GO:0008652 | P | cellular amino acid biosynthetic process | 58 | 17430 | 370 | 52012 | 1 |
| GO:0071495 | P | cellular response to endogenous stimulus | 7 | 17430 | 11 | 52012 | 0.14 |
| GO:1903509 | P | liposaccharide metabolic process | 31 | 17430 | 123 | 52012 | 0.94 |
| GO:0006644 | P | phospholipid metabolic process | 96 | 17430 | 380 | 52012 | 1 |
| GO:0006643 | P | membrane lipid metabolic process | 40 | 17430 | 132 | 52012 | 0.74 |
| GO:0009664 | P | plant-type cell wall organization | 18 | 17430 | 64 | 52012 | 0.78 |
| GO:0006721 | P | terpenoid metabolic process | 15 | 17430 | 52 | 52012 | 0.74 |
| GO:0006720 | P | isoprenoid metabolic process | 47 | 17430 | 137 | 52012 | 0.47 |
| GO:0032970 | P | regulation of actin filament-based process | 20 | 17430 | 47 | 52012 | 0.22 |
| GO:0009247 | P | glycolipid biosynthetic process | 28 | 17430 | 99 | 52012 | 0.81 |
| GO:1903507 | P | negative regulation of nucleic acid-templated transcription | 5 | 17430 | 36 | 52012 | 0.99 |
| GO:0032870 | P | cellular response to hormone stimulus | 7 | 17430 | 11 | 52012 | 0.14 |
| GO:0019752 | P | carboxylic acid metabolic process | 398 | 17430 | 1567 | 52012 | 1 |
| GO:0016485 | P | protein processing | 20 | 17430 | 59 | 52012 | 0.53 |
| GO:0044744 | P | protein targeting to nucleus | 11 | 17430 | 27 | 52012 | 0.35 |
| GO:0032879 | P | regulation of localization | 6 | 17430 | 38 | 52012 | 0.98 |
| GO:0044743 | P | intracellular protein transmembrane import | 15 | 17430 | 43 | 52012 | 0.5 |
| GO:0090304 | P | nucleic acid metabolic process | 1900 | 17430 | 5491 | 52012 | 0.1 |
| GO:0033036 | P | macromolecule localization | 286 | 17430 | 1630 | 52012 | 1 |
| GO:0051604 | P | protein maturation | 20 | 17430 | 59 | 52012 | 0.53 |
| GO:0051253 | P | negative regulation of RNA metabolic process | 5 | 17430 | 36 | 52012 | 0.99 |
| GO:0051252 | P | regulation of RNA metabolic process | 1198 | 17430 | 3329 | 52012 | 0.015 |
| GO:0001932 | P | regulation of protein phosphorylation | 20 | 17430 | 141 | 52012 | 1 |
| GO:0030071 | P | regulation of mitotic metaphase/anaphase transition | 7 | 17430 | 7 | 52012 | 0.039 |
| GO:0000956 | P | nuclear-transcribed mRNA catabolic process | 5 | 17430 | 14 | 52012 | 0.54 |
| GO:0046040 | P | IMP metabolic process | 9 | 17430 | 43 | 52012 | 0.93 |
| GO:0006006 | P | glucose metabolic process | 18 | 17430 | 85 | 52012 | 0.98 |
| GO:0034504 | P | protein localization to nucleus | 11 | 17430 | 27 | 52012 | 0.35 |
| GO:0009185 | P | ribonucleoside diphosphate metabolic process | 50 | 17430 | 187 | 52012 | 0.94 |
| GO:0005992 | P | trehalose biosynthetic process | 24 | 17430 | 74 | 52012 | 0.59 |
| GO:0006767 | P | water-soluble vitamin metabolic process | 24 | 17430 | 74 | 52012 | 0.59 |
| GO:0072524 | P | pyridine-containing compound metabolic process | 74 | 17430 | 265 | 52012 | 0.93 |
| GO:0009432 | P | SOS response | 6 | 17430 | 8 | 52012 | 0.11 |
| GO:0072521 | P | purine-containing compound metabolic process | 156 | 17430 | 658 | 52012 | 1 |
| GO:0000105 | P | histidine biosynthetic process | 12 | 17430 | 51 | 52012 | 0.9 |
| GO:0043269 | P | regulation of ion transport | 6 | 17430 | 38 | 52012 | 0.98 |
| GO:0006760 | P | folic acid-containing compound metabolic process | 10 | 17430 | 46 | 52012 | 0.92 |
| GO:0030832 | P | regulation of actin filament length | 20 | 17430 | 47 | 52012 | 0.22 |
| GO:0030833 | P | regulation of actin filament polymerization | 20 | 17430 | 47 | 52012 | 0.22 |
| GO:0001522 | P | pseudouridine synthesis | 21 | 17430 | 52 | 52012 | 0.27 |
| GO:0006733 | P | oxidoreduction coenzyme metabolic process | 69 | 17430 | 277 | 52012 | 0.99 |
| GO:0072525 | P | pyridine-containing compound biosynthetic process | 14 | 17430 | 24 | 52012 | 0.073 |
| GO:0071705 | P | nitrogen compound transport | 55 | 17430 | 220 | 52012 | 0.98 |
| GO:0071704 | P | organic substance metabolic process | 6408 | 17430 | 19075 | 52012 | 0.42 |
| GO:0071310 | P | cellular response to organic substance | 7 | 17430 | 11 | 52012 | 0.14 |
| GO:0071702 | P | organic substance transport | 340 | 17430 | 1805 | 52012 | 1 |
| GO:0015698 | P | inorganic anion transport | 42 | 17430 | 94 | 52012 | 0.075 |
| GO:0071824 | P | protein-DNA complex subunit organization | 31 | 17430 | 163 | 52012 | 1 |
| GO:0006855 | P | drug transmembrane transport | 63 | 17430 | 165 | 52012 | 0.21 |
| GO:0034613 | P | cellular protein localization | 219 | 17430 | 1046 | 52012 | 1 |
| GO:0006913 | P | nucleocytoplasmic transport | 15 | 17430 | 223 | 52012 | 1 |
| GO:0018298 | P | protein-chromophore linkage | 5 | 17430 | 14 | 52012 | 0.54 |
| GO:0016226 | P | iron-sulfur cluster assembly | 12 | 17430 | 22 | 52012 | 0.12 |
| GO:1902679 | P | negative regulation of RNA biosynthetic process | 5 | 17430 | 36 | 52012 | 0.99 |
| GO:0009112 | P | nucleobase metabolic process | 5 | 17430 | 53 | 52012 | 1 |
| GO:0009058 | P | biosynthetic process | 2848 | 17430 | 8504 | 52012 | 0.52 |
| GO:0009110 | P | vitamin biosynthetic process | 24 | 17430 | 67 | 52012 | 0.43 |
| GO:0009117 | P | nucleotide metabolic process | 195 | 17430 | 793 | 52012 | 1 |
| GO:0009116 | P | nucleoside metabolic process | 167 | 17430 | 631 | 52012 | 1 |
| GO:0051172 | P | negative regulation of nitrogen compound metabolic process | 11 | 17430 | 46 | 52012 | 0.88 |
| GO:0051173 | P | positive regulation of nitrogen compound metabolic process | 14 | 17430 | 80 | 52012 | 0.99 |
| GO:0009052 | P | pentose-phosphate shunt, non-oxidative branch | 6 | 17430 | 7 | 52012 | 0.082 |
| GO:0009119 | P | ribonucleoside metabolic process | 145 | 17430 | 566 | 52012 | 1 |
| GO:0009208 | P | pyrimidine ribonucleoside triphosphate metabolic process | 5 | 17430 | 10 | 52012 | 0.32 |
| GO:0009056 | P | catabolic process | 277 | 17430 | 1185 | 52012 | 1 |
| GO:0051179 | P | localization | 1522 | 17430 | 5066 | 52012 | 1 |
| GO:1902578 | P | single-organism localization | 404 | 17430 | 1263 | 52012 | 0.8 |
| GO:0051641 | P | cellular localization | 259 | 17430 | 1124 | 52012 | 1 |
| GO:0018342 | P | protein prenylation | 5 | 17430 | 8 | 52012 | 0.21 |
| GO:0042180 | P | cellular ketone metabolic process | 9 | 17430 | 28 | 52012 | 0.61 |
| GO:0051726 | P | regulation of cell cycle | 50 | 17430 | 168 | 52012 | 0.79 |
| GO:0090151 | P | establishment of protein localization to mitochondrial membrane | 6 | 17430 | 35 | 52012 | 0.96 |
| GO:0006754 | P | ATP biosynthetic process | 42 | 17430 | 201 | 52012 | 1 |
| GO:0000079 | P | regulation of cyclin-dependent protein serine/threonine kinase activity | 12 | 17430 | 130 | 52012 | 1 |
| GO:1901657 | P | glycosyl compound metabolic process | 167 | 17430 | 631 | 52012 | 1 |
| GO:0046451 | P | diaminopimelate metabolic process | 5 | 17430 | 28 | 52012 | 0.94 |
| GO:1902582 | P | single-organism intracellular transport | 40 | 17430 | 102 | 52012 | 0.23 |
| GO:1902580 | P | single-organism cellular localization | 51 | 17430 | 123 | 52012 | 0.12 |
| GO:0007091 | P | metaphase/anaphase transition of mitotic cell cycle | 7 | 17430 | 7 | 52012 | 0.039 |
| GO:1901659 | P | glycosyl compound biosynthetic process | 56 | 17430 | 250 | 52012 | 1 |
| GO:0045905 | P | positive regulation of translational termination | 10 | 17430 | 25 | 52012 | 0.38 |
| GO:0080090 | P | regulation of primary metabolic process | 1244 | 17430 | 3507 | 52012 | 0.039 |
| GO:0018904 | P | ether metabolic process | 42 | 17430 | 133 | 52012 | 0.66 |
| GO:0001510 | P | RNA methylation | 15 | 17430 | 34 | 52012 | 0.23 |
| GO:0061024 | P | membrane organization | 38 | 17430 | 109 | 52012 | 0.45 |
| GO:0006820 | P | anion transport | 76 | 17430 | 181 | 52012 | 0.058 |
| GO:0006821 | P | chloride transport | 12 | 17430 | 35 | 52012 | 0.53 |
| GO:0006826 | P | iron ion transport | 8 | 17430 | 28 | 52012 | 0.72 |
| GO:0008272 | P | sulfate transport | 19 | 17430 | 43 | 52012 | 0.19 |
| GO:0009755 | P | hormone-mediated signaling pathway | 7 | 17430 | 11 | 52012 | 0.14 |
| GO:0071554 | P | cell wall organization or biogenesis | 101 | 17430 | 237 | 52012 | 0.026 |
| GO:0010605 | P | negative regulation of macromolecule metabolic process | 28 | 17430 | 56 | 52012 | 0.056 |
| GO:0010604 | P | positive regulation of macromolecule metabolic process | 22 | 17430 | 80 | 52012 | 0.83 |
| GO:0070727 | P | cellular macromolecule localization | 219 | 17430 | 1046 | 52012 | 1 |
| GO:0010608 | P | posttranscriptional regulation of gene expression | 18 | 17430 | 40 | 52012 | 0.18 |
| GO:0006547 | P | histidine metabolic process | 12 | 17430 | 54 | 52012 | 0.93 |
| GO:0018193 | P | peptidyl-amino acid modification | 73 | 17430 | 171 | 52012 | 0.05 |
| GO:0006542 | P | glutamine biosynthetic process | 7 | 17430 | 19 | 52012 | 0.49 |
| GO:0006265 | P | DNA topological change | 10 | 17430 | 25 | 52012 | 0.38 |
| GO:0006260 | P | DNA replication | 59 | 17430 | 187 | 52012 | 0.68 |
| GO:0006261 | P | DNA-dependent DNA replication | 11 | 17430 | 33 | 52012 | 0.56 |
| GO:0006388 | P | tRNA splicing, via endonucleolytic cleavage and ligation | 12 | 17430 | 28 | 52012 | 0.29 |
| GO:0006383 | P | transcription from RNA polymerase III promoter | 7 | 17430 | 10 | 52012 | 0.11 |
| GO:0009206 | P | purine ribonucleoside triphosphate biosynthetic process | 47 | 17430 | 211 | 52012 | 1 |
| GO:0009205 | P | purine ribonucleoside triphosphate metabolic process | 118 | 17430 | 488 | 52012 | 1 |
| GO:0006766 | P | vitamin metabolic process | 24 | 17430 | 74 | 52012 | 0.59 |
| GO:0006448 | P | regulation of translational elongation | 13 | 17430 | 35 | 52012 | 0.43 |
| GO:0006449 | P | regulation of translational termination | 10 | 17430 | 25 | 52012 | 0.38 |
| GO:0009201 | P | ribonucleoside triphosphate biosynthetic process | 47 | 17430 | 211 | 52012 | 1 |
| GO:0043648 | P | dicarboxylic acid metabolic process | 11 | 17430 | 147 | 52012 | 1 |
| GO:0060255 | P | regulation of macromolecule metabolic process | 1262 | 17430 | 3517 | 52012 | 0.016 |
| GO:0009209 | P | pyrimidine ribonucleoside triphosphate biosynthetic process | 5 | 17430 | 10 | 52012 | 0.32 |
| GO:0032787 | P | monocarboxylic acid metabolic process | 173 | 17430 | 513 | 52012 | 0.49 |
| GO:0030163 | P | protein catabolic process | 115 | 17430 | 518 | 52012 | 1 |
| GO:0072329 | P | monocarboxylic acid catabolic process | 9 | 17430 | 21 | 52012 | 0.33 |
| GO:0030041 | P | actin filament polymerization | 20 | 17430 | 49 | 52012 | 0.27 |
| GO:0046129 | P | purine ribonucleoside biosynthetic process | 54 | 17430 | 242 | 52012 | 1 |
| GO:0046128 | P | purine ribonucleoside metabolic process | 140 | 17430 | 551 | 52012 | 1 |
| GO:0010876 | P | lipid localization | 26 | 17430 | 254 | 52012 | 1 |
| GO:0070585 | P | protein localization to mitochondrion | 11 | 17430 | 36 | 52012 | 0.66 |
| GO:1901271 | P | lipooligosaccharide biosynthetic process | 8 | 17430 | 25 | 52012 | 0.61 |
| GO:0006040 | P | amino sugar metabolic process | 15 | 17430 | 46 | 52012 | 0.59 |
| GO:0007205 | P | protein kinase C-activating G-protein coupled receptor signaling pathway | 8 | 17430 | 42 | 52012 | 0.96 |
| GO:0043647 | P | inositol phosphate metabolic process | 8 | 17430 | 28 | 52012 | 0.72 |
| GO:0048878 | P | chemical homeostasis | 18 | 17430 | 49 | 52012 | 0.41 |
| GO:0006163 | P | purine nucleotide metabolic process | 154 | 17430 | 617 | 52012 | 1 |
| GO:0043038 | P | amino acid activation | 72 | 17430 | 235 | 52012 | 0.77 |
| GO:0043039 | P | tRNA aminoacylation | 72 | 17430 | 235 | 52012 | 0.77 |
| GO:0006165 | P | nucleoside diphosphate phosphorylation | 55 | 17430 | 197 | 52012 | 0.9 |
| GO:0006164 | P | purine nucleotide biosynthetic process | 68 | 17430 | 291 | 52012 | 1 |
| GO:0019439 | P | aromatic compound catabolic process | 33 | 17430 | 150 | 52012 | 0.99 |
| GO:0019438 | P | aromatic compound biosynthetic process | 1521 | 17430 | 4449 | 52012 | 0.25 |
| GO:0006613 | P | cotranslational protein targeting to membrane | 10 | 17430 | 32 | 52012 | 0.63 |
| GO:0006612 | P | protein targeting to membrane | 15 | 17430 | 32 | 52012 | 0.18 |
| GO:0006614 | P | SRP-dependent cotranslational protein targeting to membrane | 10 | 17430 | 32 | 52012 | 0.63 |
| GO:0072488 | P | ammonium transmembrane transport | 10 | 17430 | 17 | 52012 | 0.12 |
| GO:0032940 | P | secretion by cell | 39 | 17430 | 83 | 52012 | 0.053 |
| GO:0031365 | P | N-terminal protein amino acid modification | 5 | 17430 | 7 | 52012 | 0.16 |
| GO:1901576 | P | organic substance biosynthetic process | 2721 | 17430 | 8122 | 52012 | 0.51 |
| GO:1901575 | P | organic substance catabolic process | 273 | 17430 | 1139 | 52012 | 1 |
| GO:0006553 | P | lysine metabolic process | 5 | 17430 | 28 | 52012 | 0.94 |
| GO:0016042 | P | lipid catabolic process | 18 | 17430 | 73 | 52012 | 0.9 |
| GO:0065003 | P | macromolecular complex assembly | 95 | 17430 | 429 | 52012 | 1 |
| GO:0065002 | P | intracellular protein transmembrane transport | 19 | 17430 | 43 | 52012 | 0.19 |
| GO:0033015 | P | tetrapyrrole catabolic process | 5 | 17430 | 13 | 52012 | 0.49 |
| GO:0033014 | P | tetrapyrrole biosynthetic process | 27 | 17430 | 121 | 52012 | 0.98 |
| GO:0033013 | P | tetrapyrrole metabolic process | 32 | 17430 | 134 | 52012 | 0.97 |
| GO:0098662 | P | inorganic cation transmembrane transport | 127 | 17430 | 389 | 52012 | 0.62 |
| GO:0098660 | P | inorganic ion transmembrane transport | 129 | 17430 | 393 | 52012 | 0.6 |
| GO:0032011 | P | ARF protein signal transduction | 12 | 17430 | 33 | 52012 | 0.46 |
| GO:0065009 | P | regulation of molecular function | 34 | 17430 | 248 | 52012 | 1 |
| GO:0006505 | P | GPI anchor metabolic process | 21 | 17430 | 72 | 52012 | 0.75 |
| GO:0009156 | P | ribonucleoside monophosphate biosynthetic process | 53 | 17430 | 273 | 52012 | 1 |
| GO:0009152 | P | purine ribonucleotide biosynthetic process | 63 | 17430 | 275 | 52012 | 1 |
| GO:0009150 | P | purine ribonucleotide metabolic process | 149 | 17430 | 584 | 52012 | 1 |
| GO:0016117 | P | carotenoid biosynthetic process | 5 | 17430 | 23 | 52012 | 0.87 |
| GO:0044802 | P | single-organism membrane organization | 33 | 17430 | 90 | 52012 | 0.36 |
| GO:0043254 | P | regulation of protein complex assembly | 20 | 17430 | 47 | 52012 | 0.22 |
| GO:0016114 | P | terpenoid biosynthetic process | 15 | 17430 | 52 | 52012 | 0.74 |
| GO:0045047 | P | protein targeting to ER | 14 | 17430 | 32 | 52012 | 0.25 |
| GO:0009069 | P | serine family amino acid metabolic process | 11 | 17430 | 140 | 52012 | 1 |
| GO:0034728 | P | nucleosome organization | 26 | 17430 | 163 | 52012 | 1 |
| GO:0015936 | P | coenzyme A metabolic process | 14 | 17430 | 31 | 52012 | 0.22 |
| GO:0006869 | P | lipid transport | 26 | 17430 | 254 | 52012 | 1 |
| GO:0016311 | P | dephosphorylation | 145 | 17430 | 347 | 52012 | 0.015 |
| GO:0005984 | P | disaccharide metabolic process | 41 | 17430 | 132 | 52012 | 0.69 |
| GO:0050801 | P | ion homeostasis | 10 | 17430 | 49 | 52012 | 0.95 |
| GO:0055065 | P | metal ion homeostasis | 5 | 17430 | 18 | 52012 | 0.72 |
| GO:0016070 | P | RNA metabolic process | 1673 | 17430 | 4744 | 52012 | 0.031 |
| GO:0044248 | P | cellular catabolic process | 186 | 17430 | 767 | 52012 | 1 |
| GO:0044249 | P | cellular biosynthetic process | 2670 | 17430 | 8039 | 52012 | 0.67 |
| GO:0034641 | P | cellular nitrogen compound metabolic process | 2836 | 17430 | 8317 | 52012 | 0.19 |
| GO:0023051 | P | regulation of signaling | 18 | 17430 | 71 | 52012 | 0.88 |
| GO:0010498 | P | proteasomal protein catabolic process | 8 | 17430 | 34 | 52012 | 0.86 |
| GO:0009123 | P | nucleoside monophosphate metabolic process | 124 | 17430 | 550 | 52012 | 1 |
| GO:0044242 | P | cellular lipid catabolic process | 9 | 17430 | 34 | 52012 | 0.79 |
| GO:0010646 | P | regulation of cell communication | 18 | 17430 | 71 | 52012 | 0.88 |
| GO:0009126 | P | purine nucleoside monophosphate metabolic process | 121 | 17430 | 515 | 52012 | 1 |
| GO:0009127 | P | purine nucleoside monophosphate biosynthetic process | 53 | 17430 | 251 | 52012 | 1 |
| GO:0009124 | P | nucleoside monophosphate biosynthetic process | 56 | 17430 | 286 | 52012 | 1 |
| GO:0006220 | P | pyrimidine nucleotide metabolic process | 20 | 17430 | 61 | 52012 | 0.58 |
| GO:0006221 | P | pyrimidine nucleotide biosynthetic process | 19 | 17430 | 60 | 52012 | 0.63 |
| GO:0007264 | P | small GTPase mediated signal transduction | 108 | 17430 | 478 | 52012 | 1 |
| GO:0046834 | P | lipid phosphorylation | 18 | 17430 | 142 | 52012 | 1 |
| GO:0006228 | P | UTP biosynthetic process | 5 | 17430 | 10 | 52012 | 0.32 |
| GO:0071103 | P | DNA conformation change | 42 | 17430 | 190 | 52012 | 1 |
| GO:0000041 | P | transition metal ion transport | 20 | 17430 | 71 | 52012 | 0.79 |
| GO:0046939 | P | nucleotide phosphorylation | 55 | 17430 | 229 | 52012 | 0.99 |
| GO:0022618 | P | ribonucleoprotein complex assembly | 11 | 17430 | 14 | 52012 | 0.03 |
| GO:0046467 | P | membrane lipid biosynthetic process | 28 | 17430 | 99 | 52012 | 0.81 |
| GO:1901605 | P | alpha-amino acid metabolic process | 78 | 17430 | 392 | 52012 | 1 |
| GO:1901607 | P | alpha-amino acid biosynthetic process | 45 | 17430 | 246 | 52012 | 1 |
| GO:1901606 | P | alpha-amino acid catabolic process | 15 | 17430 | 35 | 52012 | 0.26 |
| GO:0052646 | P | alditol phosphate metabolic process | 5 | 17430 | 34 | 52012 | 0.98 |
| GO:0009628 | P | response to abiotic stimulus | 27 | 17430 | 187 | 52012 | 1 |
| GO:0017038 | P | protein import | 28 | 17430 | 78 | 52012 | 0.41 |
| GO:0044106 | P | cellular amine metabolic process | 24 | 17430 | 55 | 52012 | 0.17 |
| GO:0030001 | P | metal ion transport | 174 | 17430 | 575 | 52012 | 0.89 |
| GO:0001101 | P | response to acid chemical | 11 | 17430 | 24 | 52012 | 0.25 |
| GO:0030003 | P | cellular cation homeostasis | 5 | 17430 | 18 | 52012 | 0.72 |
| GO:0055080 | P | cation homeostasis | 5 | 17430 | 49 | 52012 | 1 |
| GO:0034035 | P | purine ribonucleoside bisphosphate metabolic process | 6 | 17430 | 20 | 52012 | 0.67 |
| GO:0034030 | P | ribonucleoside bisphosphate biosynthetic process | 5 | 17430 | 19 | 52012 | 0.76 |
| GO:0055085 | P | transmembrane transport | 741 | 17430 | 2073 | 52012 | 0.065 |
| GO:0034032 | P | purine nucleoside bisphosphate metabolic process | 20 | 17430 | 51 | 52012 | 0.32 |
| GO:0034033 | P | purine nucleoside bisphosphate biosynthetic process | 5 | 17430 | 19 | 52012 | 0.76 |
| GO:0043604 | P | amide biosynthetic process | 640 | 17430 | 1753 | 52012 | 0.032 |
| GO:0032268 | P | regulation of cellular protein metabolic process | 37 | 17430 | 191 | 52012 | 1 |
| GO:0006081 | P | cellular aldehyde metabolic process | 17 | 17430 | 86 | 52012 | 0.99 |
| GO:0006082 | P | organic acid metabolic process | 447 | 17430 | 1768 | 52012 | 1 |
| GO:0005996 | P | monosaccharide metabolic process | 28 | 17430 | 201 | 52012 | 1 |
| GO:0051258 | P | protein polymerization | 24 | 17430 | 155 | 52012 | 1 |
| GO:0042364 | P | water-soluble vitamin biosynthetic process | 24 | 17430 | 67 | 52012 | 0.43 |
| GO:0005991 | P | trehalose metabolic process | 26 | 17430 | 77 | 52012 | 0.52 |
| GO:0008213 | P | protein alkylation | 34 | 17430 | 71 | 52012 | 0.057 |
| GO:0010628 | P | positive regulation of gene expression | 13 | 17430 | 80 | 52012 | 1 |
| GO:0009987 | P | cellular process | 6786 | 17430 | 20063 | 52012 | 0.2 |
| GO:0006801 | P | superoxide metabolic process | 10 | 17430 | 52 | 52012 | 0.97 |
| GO:0006650 | P | glycerophospholipid metabolic process | 69 | 17430 | 280 | 52012 | 0.99 |
| GO:0032012 | P | regulation of ARF protein signal transduction | 12 | 17430 | 33 | 52012 | 0.46 |
| GO:0031327 | P | negative regulation of cellular biosynthetic process | 10 | 17430 | 46 | 52012 | 0.92 |
| GO:0031325 | P | positive regulation of cellular metabolic process | 22 | 17430 | 80 | 52012 | 0.83 |
| GO:0031324 | P | negative regulation of cellular metabolic process | 11 | 17430 | 46 | 52012 | 0.88 |
| GO:0031323 | P | regulation of cellular metabolic process | 1244 | 17430 | 3502 | 52012 | 0.035 |
| GO:0019751 | P | polyol metabolic process | 17 | 17430 | 88 | 52012 | 0.99 |
| GO:0009231 | P | riboflavin biosynthetic process | 9 | 17430 | 37 | 52012 | 0.85 |
| GO:0044770 | P | cell cycle phase transition | 8 | 17430 | 9 | 52012 | 0.041 |
| GO:0044772 | P | mitotic cell cycle phase transition | 8 | 17430 | 9 | 52012 | 0.041 |
| GO:0046112 | P | nucleobase biosynthetic process | 5 | 17430 | 46 | 52012 | 1 |
| GO:0032507 | P | maintenance of protein location in cell | 11 | 17430 | 34 | 52012 | 0.6 |
| GO:0015893 | P | drug transport | 63 | 17430 | 165 | 52012 | 0.21 |
| GO:0030488 | P | tRNA methylation | 10 | 17430 | 15 | 52012 | 0.073 |
| GO:0071826 | P | ribonucleoprotein complex subunit organization | 11 | 17430 | 14 | 52012 | 0.03 |
| GO:0071822 | P | protein complex subunit organization | 123 | 17430 | 469 | 52012 | 0.99 |
| GO:0019682 | P | glyceraldehyde-3-phosphate metabolic process | 13 | 17430 | 59 | 52012 | 0.94 |
| GO:0006013 | P | mannose metabolic process | 7 | 17430 | 33 | 52012 | 0.91 |
| GO:0097354 | P | prenylation | 5 | 17430 | 8 | 52012 | 0.21 |
| GO:0006400 | P | tRNA modification | 18 | 17430 | 47 | 52012 | 0.36 |
| GO:0006401 | P | RNA catabolic process | 10 | 17430 | 41 | 52012 | 0.86 |
| GO:0006402 | P | mRNA catabolic process | 10 | 17430 | 23 | 52012 | 0.3 |
| GO:0009581 | P | detection of external stimulus | 5 | 17430 | 14 | 52012 | 0.54 |
| GO:0009582 | P | detection of abiotic stimulus | 5 | 17430 | 14 | 52012 | 0.54 |
| GO:0009583 | P | detection of light stimulus | 5 | 17430 | 14 | 52012 | 0.54 |
| GO:0009584 | P | detection of visible light | 5 | 17430 | 14 | 52012 | 0.54 |
| GO:0048193 | P | Golgi vesicle transport | 62 | 17430 | 153 | 52012 | 0.12 |
| GO:0009073 | P | aromatic amino acid family biosynthetic process | 22 | 17430 | 79 | 52012 | 0.81 |
| GO:0006725 | P | cellular aromatic compound metabolic process | 2257 | 17430 | 6894 | 52012 | 0.85 |
| GO:0015748 | P | organophosphate ester transport | 18 | 17430 | 43 | 52012 | 0.25 |
| GO:0044264 | P | cellular polysaccharide metabolic process | 85 | 17430 | 297 | 52012 | 0.91 |
| GO:0044265 | P | cellular macromolecule catabolic process | 121 | 17430 | 485 | 52012 | 1 |
| GO:0009245 | P | lipid A biosynthetic process | 8 | 17430 | 25 | 52012 | 0.61 |
| GO:0044262 | P | cellular carbohydrate metabolic process | 144 | 17430 | 537 | 52012 | 0.99 |
| GO:0042775 | P | mitochondrial ATP synthesis coupled electron transport | 7 | 17430 | 21 | 52012 | 0.58 |
| GO:0042773 | P | ATP synthesis coupled electron transport | 9 | 17430 | 46 | 52012 | 0.96 |
| GO:0007018 | P | microtubule-based movement | 92 | 17430 | 328 | 52012 | 0.94 |
| GO:0044087 | P | regulation of cellular component biogenesis | 21 | 17430 | 48 | 52012 | 0.19 |
| GO:0044085 | P | cellular component biogenesis | 201 | 17430 | 589 | 52012 | 0.43 |
| GO:0002252 | P | immune effector process | 5 | 17430 | 19 | 52012 | 0.76 |
| GO:0015031 | P | protein transport | 236 | 17430 | 1302 | 52012 | 1 |
| GO:0098655 | P | cation transmembrane transport | 137 | 17430 | 406 | 52012 | 0.49 |
| GO:0043531 | F | ADP binding | 239 | 17430 | 379 | 52012 | 5.8E-14 |
| GO:0020037 | F | heme binding | 427 | 17430 | 842 | 52012 | 4E-12 |
| GO:0046906 | F | tetrapyrrole binding | 434 | 17430 | 864 | 52012 | 7.3E-12 |
| GO:0030246 | F | carbohydrate binding | 194 | 17430 | 314 | 52012 | 4.5E-11 |
| GO:0008092 | F | cytoskeletal protein binding | 138 | 17430 | 201 | 52012 | 2E-10 |
| GO:0004672 | F | protein kinase activity | 1413 | 17430 | 3556 | 52012 | 1.5E-08 |
| GO:0060589 | F | nucleoside-triphosphatase regulator activity | 48 | 17430 | 48 | 52012 | 1.3E-07 |
| GO:0030234 | F | enzyme regulator activity | 223 | 17430 | 430 | 52012 | 1.3E-07 |
| GO:0001871 | F | pattern binding | 106 | 17430 | 166 | 52012 | 3.1E-07 |
| GO:0030247 | F | polysaccharide binding | 106 | 17430 | 166 | 52012 | 3.1E-07 |
| GO:0004722 | F | protein serine/threonine phosphatase activity | 116 | 17430 | 191 | 52012 | 5.8E-07 |
| GO:0019787 | F | ubiquitin-like protein transferase activity | 130 | 17430 | 229 | 52012 | 1.8E-06 |
| GO:0098772 | F | molecular function regulator | 252 | 17430 | 524 | 52012 | 2.1E-06 |
| GO:0016491 | F | oxidoreductase activity | 1648 | 17430 | 4329 | 52012 | 2.6E-06 |
| GO:0004842 | F | ubiquitin-protein transferase activity | 127 | 17430 | 229 | 52012 | 5.7E-06 |
| GO:0047134 | F | protein-disulfide reductase activity | 79 | 17430 | 123 | 52012 | 7.9E-06 |
| GO:0016758 | F | transferase activity, transferring hexosyl groups | 375 | 17430 | 878 | 52012 | 0.000053 |
| GO:0004857 | F | enzyme inhibitor activity | 120 | 17430 | 230 | 52012 | 0.000075 |
| GO:0016301 | F | kinase activity | 1551 | 17430 | 4158 | 52012 | 0.0001 |
| GO:0016773 | F | phosphotransferase activity, alcohol group as acceptor | 1550 | 17430 | 4173 | 52012 | 0.00018 |
| GO:0042393 | F | histone binding | 30 | 17430 | 35 | 52012 | 0.00019 |
| GO:0016740 | F | transferase activity | 3055 | 17430 | 8540 | 52012 | 0.00038 |
| GO:0016667 | F | oxidoreductase activity, acting on a sulfur group of donors | 192 | 17430 | 439 | 52012 | 0.0014 |
| GO:0003735 | F | structural constituent of ribosome | 479 | 17430 | 1218 | 52012 | 0.0016 |
| GO:0016757 | F | transferase activity, transferring glycosyl groups | 458 | 17430 | 1169 | 52012 | 0.0025 |
| GO:0016668 | F | oxidoreductase activity, acting on a sulfur group of donors, NAD(P) as acceptor | 79 | 17430 | 158 | 52012 | 0.0027 |
| GO:0008234 | F | cysteine-type peptidase activity | 96 | 17430 | 208 | 52012 | 0.0063 |
| GO:0061733 | F | peptide-lysine-N-acetyltransferase activity | 15 | 17430 | 25 | 52012 | 0.056 |
| GO:0030291 | F | protein serine/threonine kinase inhibitor activity | 10 | 17430 | 20 | 52012 | 0.2 |
| GO:0042085 | F | 5-methyltetrahydropteroyltri-L-glutamate-dependent methyltransferase activity | 6 | 17430 | 26 | 52012 | 0.85 |
| GO:0031072 | F | heat shock protein binding | 10 | 17430 | 387 | 52012 | 1 |
| GO:0005488 | F | binding | 8914 | 17430 | 34020 | 52012 | 1 |
| GO:0016876 | F | ligase activity, forming aminoacyl-tRNA and related compounds | 67 | 17430 | 235 | 52012 | 0.89 |
| GO:0016877 | F | ligase activity, forming carbon-sulfur bonds | 17 | 17430 | 45 | 52012 | 0.38 |
| GO:0004536 | F | deoxyribonuclease activity | 8 | 17430 | 17 | 52012 | 0.28 |
| GO:0008026 | F | ATP-dependent helicase activity | 15 | 17430 | 326 | 52012 | 1 |
| GO:0000287 | F | magnesium ion binding | 136 | 17430 | 426 | 52012 | 0.7 |
| GO:0004448 | F | isocitrate dehydrogenase activity | 5 | 17430 | 38 | 52012 | 0.99 |
| GO:0008289 | F | lipid binding | 101 | 17430 | 412 | 52012 | 1 |
| GO:0048037 | F | cofactor binding | 370 | 17430 | 1648 | 52012 | 1 |
| GO:0048038 | F | quinone binding | 12 | 17430 | 72 | 52012 | 0.99 |
| GO:0004664 | F | prephenate dehydratase activity | 6 | 17430 | 19 | 52012 | 0.63 |
| GO:0004197 | F | cysteine-type endopeptidase activity | 20 | 17430 | 155 | 52012 | 1 |
| GO:0004190 | F | aspartic-type endopeptidase activity | 83 | 17430 | 212 | 52012 | 0.13 |
| GO:0003899 | F | DNA-directed RNA polymerase activity | 72 | 17430 | 200 | 52012 | 0.32 |
| GO:0003872 | F | 6-phosphofructokinase activity | 10 | 17430 | 33 | 52012 | 0.67 |
| GO:0003871 | F | 5-methyltetrahydropteroyltriglutamate-homocysteine S-methyltransferase activity | 6 | 17430 | 26 | 52012 | 0.85 |
| GO:0015215 | F | nucleotide transmembrane transporter activity | 5 | 17430 | 12 | 52012 | 0.43 |
| GO:0015217 | F | ADP transmembrane transporter activity | 5 | 17430 | 12 | 52012 | 0.43 |
| GO:0015216 | F | purine nucleotide transmembrane transporter activity | 5 | 17430 | 12 | 52012 | 0.43 |
| GO:0004066 | F | asparagine synthase (glutamine-hydrolyzing) activity | 8 | 17430 | 25 | 52012 | 0.61 |
| GO:0019210 | F | kinase inhibitor activity | 10 | 17430 | 20 | 52012 | 0.2 |
| GO:0019213 | F | deacetylase activity | 6 | 17430 | 27 | 52012 | 0.87 |
| GO:0016638 | F | oxidoreductase activity, acting on the CH-NH2 group of donors | 13 | 17430 | 61 | 52012 | 0.95 |
| GO:0016634 | F | oxidoreductase activity, acting on the CH-CH group of donors, oxygen as acceptor | 12 | 17430 | 49 | 52012 | 0.87 |
| GO:0016987 | F | sigma factor activity | 8 | 17430 | 30 | 52012 | 0.77 |
| GO:0016742 | F | hydroxymethyl-, formyl- and related transferase activity | 8 | 17430 | 70 | 52012 | 1 |
| GO:0016741 | F | transferase activity, transferring one-carbon groups | 277 | 17430 | 818 | 52012 | 0.45 |
| GO:0016746 | F | transferase activity, transferring acyl groups | 271 | 17430 | 777 | 52012 | 0.29 |
| GO:0016747 | F | transferase activity, transferring acyl groups other than amino-acyl groups | 213 | 17430 | 617 | 52012 | 0.37 |
| GO:0016744 | F | transferase activity, transferring aldehyde or ketonic groups | 6 | 17430 | 38 | 52012 | 0.98 |
| GO:0016168 | F | chlorophyll binding | 7 | 17430 | 22 | 52012 | 0.62 |
| GO:0016160 | F | amylase activity | 21 | 17430 | 68 | 52012 | 0.67 |
| GO:0016161 | F | beta-amylase activity | 14 | 17430 | 49 | 52012 | 0.74 |
| GO:0016298 | F | lipase activity | 10 | 17430 | 218 | 52012 | 1 |
| GO:0051540 | F | metal cluster binding | 69 | 17430 | 249 | 52012 | 0.93 |
| GO:0022890 | F | inorganic cation transmembrane transporter activity | 222 | 17430 | 710 | 52012 | 0.83 |
| GO:0022892 | F | substrate-specific transporter activity | 467 | 17430 | 1691 | 52012 | 1 |
| GO:0036442 | F | hydrogen-exporting ATPase activity | 26 | 17430 | 97 | 52012 | 0.87 |
| GO:0004525 | F | ribonuclease III activity | 13 | 17430 | 41 | 52012 | 0.62 |
| GO:0000774 | F | adenyl-nucleotide exchange factor activity | 6 | 17430 | 13 | 52012 | 0.34 |
| GO:0004527 | F | exonuclease activity | 27 | 17430 | 113 | 52012 | 0.96 |
| GO:0004521 | F | endoribonuclease activity | 30 | 17430 | 114 | 52012 | 0.9 |
| GO:0004520 | F | endodeoxyribonuclease activity | 6 | 17430 | 14 | 52012 | 0.39 |
| GO:0016875 | F | ligase activity, forming carbon-oxygen bonds | 67 | 17430 | 235 | 52012 | 0.89 |
| GO:0005215 | F | transporter activity | 914 | 17430 | 2603 | 52012 | 0.11 |
| GO:0005216 | F | ion channel activity | 69 | 17430 | 217 | 52012 | 0.67 |
| GO:0031625 | F | ubiquitin protein ligase binding | 18 | 17430 | 51 | 52012 | 0.47 |
| GO:0000295 | F | adenine nucleotide transmembrane transporter activity | 5 | 17430 | 12 | 52012 | 0.43 |
| GO:0004298 | F | threonine-type endopeptidase activity | 35 | 17430 | 106 | 52012 | 0.56 |
| GO:0004450 | F | isocitrate dehydrogenase (NADP+) activity | 5 | 17430 | 27 | 52012 | 0.93 |
| GO:0005452 | F | inorganic anion exchanger activity | 12 | 17430 | 28 | 52012 | 0.29 |
| GO:0005102 | F | receptor binding | 13 | 17430 | 18 | 52012 | 0.03 |
| GO:0008725 | F | DNA-3-methyladenine glycosylase activity | 8 | 17430 | 34 | 52012 | 0.86 |
| GO:0004180 | F | carboxypeptidase activity | 38 | 17430 | 156 | 52012 | 0.97 |
| GO:0004185 | F | serine-type carboxypeptidase activity | 36 | 17430 | 132 | 52012 | 0.88 |
| GO:0003887 | F | DNA-directed DNA polymerase activity | 13 | 17430 | 64 | 52012 | 0.97 |
| GO:0004674 | F | protein serine/threonine kinase activity | 34 | 17430 | 2636 | 52012 | 1 |
| GO:0003883 | F | CTP synthase activity | 7 | 17430 | 16 | 52012 | 0.35 |
| GO:0004673 | F | protein histidine kinase activity | 20 | 17430 | 91 | 52012 | 0.97 |
| GO:1901363 | F | heterocyclic compound binding | 4994 | 17430 | 18860 | 52012 | 1 |
| GO:0016628 | F | oxidoreductase activity, acting on the CH-CH group of donors, NAD or NADP as acceptor | 7 | 17430 | 42 | 52012 | 0.98 |
| GO:0016624 | F | oxidoreductase activity, acting on the aldehyde or oxo group of donors, disulfide as acceptor | 17 | 17430 | 33 | 52012 | 0.1 |
| GO:0016627 | F | oxidoreductase activity, acting on the CH-CH group of donors | 45 | 17430 | 178 | 52012 | 0.96 |
| GO:0016620 | F | oxidoreductase activity, acting on the aldehyde or oxo group of donors, NAD or NADP as acceptor | 26 | 17430 | 157 | 52012 | 1 |
| GO:0046873 | F | metal ion transmembrane transporter activity | 87 | 17430 | 366 | 52012 | 1 |
| GO:0046872 | F | metal ion binding | 1890 | 17430 | 7694 | 52012 | 1 |
| GO:0016755 | F | transferase activity, transferring amino-acyl groups | 6 | 17430 | 21 | 52012 | 0.71 |
| GO:0000990 | F | transcription factor activity, core RNA polymerase binding | 8 | 17430 | 30 | 52012 | 0.77 |
| GO:0015405 | F | P-P-bond-hydrolysis-driven transmembrane transporter activity | 106 | 17430 | 518 | 52012 | 1 |
| GO:0000996 | F | core DNA-dependent RNA polymerase binding promoter specificity activity | 8 | 17430 | 30 | 52012 | 0.77 |
| GO:0033897 | F | ribonuclease T2 activity | 12 | 17430 | 35 | 52012 | 0.53 |
| GO:0003676 | F | nucleic acid binding | 2183 | 17430 | 8571 | 52012 | 1 |
| GO:0003677 | F | DNA binding | 1348 | 17430 | 4922 | 52012 | 1 |
| GO:0000049 | F | tRNA binding | 5 | 17430 | 15 | 52012 | 0.59 |
| GO:0046527 | F | glucosyltransferase activity | 53 | 17430 | 241 | 52012 | 1 |
| GO:0097159 | F | organic cyclic compound binding | 4994 | 17430 | 18864 | 52012 | 1 |
| GO:0042578 | F | phosphoric ester hydrolase activity | 216 | 17430 | 829 | 52012 | 1 |
| GO:0060590 | F | ATPase regulator activity | 10 | 17430 | 19 | 52012 | 0.17 |
| GO:0043168 | F | anion binding | 109 | 17430 | 590 | 52012 | 1 |
| GO:0043169 | F | cation binding | 1899 | 17430 | 8202 | 52012 | 1 |
| GO:0043167 | F | ion binding | 2005 | 17430 | 8723 | 52012 | 1 |
| GO:0017150 | F | tRNA dihydrouridine synthase activity | 6 | 17430 | 29 | 52012 | 0.9 |
| GO:0003690 | F | double-stranded DNA binding | 56 | 17430 | 111 | 52012 | 0.009 |
| GO:0003697 | F | single-stranded DNA binding | 16 | 17430 | 65 | 52012 | 0.89 |
| GO:0004860 | F | protein kinase inhibitor activity | 10 | 17430 | 20 | 52012 | 0.2 |
| GO:0004861 | F | cyclin-dependent protein serine/threonine kinase inhibitor activity | 10 | 17430 | 20 | 52012 | 0.2 |
| GO:0004866 | F | endopeptidase inhibitor activity | 22 | 17430 | 38 | 52012 | 0.031 |
| GO:0004867 | F | serine-type endopeptidase inhibitor activity | 5 | 17430 | 13 | 52012 | 0.49 |
| GO:0004869 | F | cysteine-type endopeptidase inhibitor activity | 9 | 17430 | 16 | 52012 | 0.15 |
| GO:0004518 | F | nuclease activity | 115 | 17430 | 478 | 52012 | 1 |
| GO:0004519 | F | endonuclease activity | 52 | 17430 | 236 | 52012 | 1 |
| GO:0044389 | F | ubiquitin-like protein ligase binding | 18 | 17430 | 51 | 52012 | 0.47 |
| GO:0008759 | F | UDP-3-O-[3-hydroxymyristoyl] N-acetylglucosamine deacetylase activity | 6 | 17430 | 15 | 52012 | 0.44 |
| GO:0008757 | F | S-adenosylmethionine-dependent methyltransferase activity | 41 | 17430 | 178 | 52012 | 0.99 |
| GO:0008199 | F | ferric iron binding | 6 | 17430 | 16 | 52012 | 0.49 |
| GO:0008192 | F | RNA guanylyltransferase activity | 5 | 17430 | 17 | 52012 | 0.68 |
| GO:0008194 | F | UDP-glycosyltransferase activity | 139 | 17430 | 345 | 52012 | 0.038 |
| GO:0004645 | F | phosphorylase activity | 8 | 17430 | 13 | 52012 | 0.13 |
| GO:0004888 | F | transmembrane signaling receptor activity | 23 | 17430 | 77 | 52012 | 0.72 |
| GO:0008378 | F | galactosyltransferase activity | 29 | 17430 | 81 | 52012 | 0.42 |
| GO:0008374 | F | O-acyltransferase activity | 31 | 17430 | 106 | 52012 | 0.78 |
| GO:0008375 | F | acetylglucosaminyltransferase activity | 50 | 17430 | 109 | 52012 | 0.042 |
| GO:0003857 | F | 3-hydroxyacyl-CoA dehydrogenase activity | 9 | 17430 | 21 | 52012 | 0.33 |
| GO:0008373 | F | sialyltransferase activity | 5 | 17430 | 9 | 52012 | 0.26 |
| GO:0015238 | F | drug transmembrane transporter activity | 63 | 17430 | 165 | 52012 | 0.21 |
| GO:0004089 | F | carbonate dehydratase activity | 10 | 17430 | 47 | 52012 | 0.93 |
| GO:0016614 | F | oxidoreductase activity, acting on CH-OH group of donors | 182 | 17430 | 530 | 52012 | 0.4 |
| GO:0016615 | F | malate dehydrogenase activity | 24 | 17430 | 70 | 52012 | 0.5 |
| GO:0051213 | F | dioxygenase activity | 28 | 17430 | 485 | 52012 | 1 |
| GO:0008408 | F | 3'-5' exonuclease activity | 18 | 17430 | 44 | 52012 | 0.28 |
| GO:0016765 | F | transferase activity, transferring alkyl or aryl (other than methyl) groups | 45 | 17430 | 156 | 52012 | 0.83 |
| GO:0019239 | F | deaminase activity | 8 | 17430 | 36 | 52012 | 0.89 |
| GO:0019238 | F | cyclohydrolase activity | 11 | 17430 | 42 | 52012 | 0.81 |
| GO:0016760 | F | cellulose synthase (UDP-forming) activity | 20 | 17430 | 81 | 52012 | 0.91 |
| GO:0016762 | F | xyloglucan:xyloglucosyl transferase activity | 37 | 17430 | 70 | 52012 | 0.018 |
| GO:0000988 | F | transcription factor activity, protein binding | 60 | 17430 | 146 | 52012 | 0.11 |
| GO:0050113 | F | inositol oxygenase activity | 5 | 17430 | 21 | 52012 | 0.82 |
| GO:0015144 | F | carbohydrate transmembrane transporter activity | 13 | 17430 | 48 | 52012 | 0.79 |
| GO:0016149 | F | translation release factor activity, codon specific | 8 | 17430 | 19 | 52012 | 0.36 |
| GO:0045735 | F | nutrient reservoir activity | 13 | 17430 | 78 | 52012 | 1 |
| GO:0035639 | F | purine ribonucleoside triphosphate binding | 2068 | 17430 | 7777 | 52012 | 1 |
| GO:0001104 | F | RNA polymerase II transcription cofactor activity | 15 | 17430 | 54 | 52012 | 0.78 |
| GO:0061505 | F | DNA topoisomerase II activity | 6 | 17430 | 17 | 52012 | 0.54 |
| GO:0005506 | F | iron ion binding | 367 | 17430 | 983 | 52012 | 0.041 |
| GO:0005507 | F | copper ion binding | 85 | 17430 | 282 | 52012 | 0.82 |
| GO:0003700 | F | transcription factor activity, sequence-specific DNA binding | 647 | 17430 | 1741 | 52012 | 0.012 |
| GO:0003684 | F | damaged DNA binding | 14 | 17430 | 66 | 52012 | 0.96 |
| GO:0004872 | F | receptor activity | 43 | 17430 | 159 | 52012 | 0.91 |
| GO:0004871 | F | signal transducer activity | 74 | 17430 | 536 | 52012 | 1 |
| GO:0050662 | F | coenzyme binding | 267 | 17430 | 1021 | 52012 | 1 |
| GO:0050661 | F | NADP binding | 29 | 17430 | 165 | 52012 | 1 |
| GO:0050660 | F | flavin adenine dinucleotide binding | 108 | 17430 | 386 | 52012 | 0.96 |
| GO:0050664 | F | oxidoreductase activity, acting on NAD(P)H, oxygen as acceptor | 11 | 17430 | 27 | 52012 | 0.35 |
| GO:0004478 | F | methionine adenosyltransferase activity | 8 | 17430 | 18 | 52012 | 0.32 |
| GO:0004470 | F | malic enzyme activity | 20 | 17430 | 43 | 52012 | 0.14 |
| GO:0005402 | F | cation:sugar symporter activity | 10 | 17430 | 35 | 52012 | 0.73 |
| GO:0005471 | F | ATP:ADP antiporter activity | 5 | 17430 | 12 | 52012 | 0.43 |
| GO:0004252 | F | serine-type endopeptidase activity | 89 | 17430 | 304 | 52012 | 0.88 |
| GO:0008964 | F | phosphoenolpyruvate carboxylase activity | 6 | 17430 | 9 | 52012 | 0.15 |
| GO:0004659 | F | prenyltransferase activity | 13 | 17430 | 41 | 52012 | 0.62 |
| GO:0004652 | F | polynucleotide adenylyltransferase activity | 7 | 17430 | 32 | 52012 | 0.89 |
| GO:0004650 | F | polygalacturonase activity | 24 | 17430 | 117 | 52012 | 0.99 |
| GO:0000989 | F | transcription factor activity, transcription factor binding | 52 | 17430 | 116 | 52012 | 0.051 |
| GO:0016763 | F | transferase activity, transferring pentosyl groups | 12 | 17430 | 112 | 52012 | 1 |
| GO:0003843 | F | 1,3-beta-D-glucan synthase activity | 16 | 17430 | 40 | 52012 | 0.32 |
| GO:0016818 | F | hydrolase activity, acting on acid anhydrides, in phosphorus-containing anhydrides | 568 | 17430 | 2759 | 52012 | 1 |
| GO:0016810 | F | hydrolase activity, acting on carbon-nitrogen (but not peptide) bonds | 57 | 17430 | 239 | 52012 | 0.99 |
| GO:0016811 | F | hydrolase activity, acting on carbon-nitrogen (but not peptide) bonds, in linear amides | 23 | 17430 | 75 | 52012 | 0.68 |
| GO:0016817 | F | hydrolase activity, acting on acid anhydrides | 584 | 17430 | 2832 | 52012 | 1 |
| GO:0016814 | F | hydrolase activity, acting on carbon-nitrogen (but not peptide) bonds, in cyclic amidines | 18 | 17430 | 76 | 52012 | 0.93 |
| GO:0016769 | F | transferase activity, transferring nitrogenous groups | 20 | 17430 | 180 | 52012 | 1 |
| GO:0004097 | F | catechol oxidase activity | 6 | 17430 | 8 | 52012 | 0.11 |
| GO:0003995 | F | acyl-CoA dehydrogenase activity | 14 | 17430 | 41 | 52012 | 0.53 |
| GO:0003997 | F | acyl-CoA oxidase activity | 9 | 17430 | 25 | 52012 | 0.49 |
| GO:0003993 | F | acid phosphatase activity | 12 | 17430 | 106 | 52012 | 1 |
| GO:0008565 | F | protein transporter activity | 33 | 17430 | 217 | 52012 | 1 |
| GO:0015399 | F | primary active transmembrane transporter activity | 106 | 17430 | 518 | 52012 | 1 |
| GO:0019887 | F | protein kinase regulator activity | 23 | 17430 | 67 | 52012 | 0.5 |
| GO:0019888 | F | protein phosphatase regulator activity | 36 | 17430 | 90 | 52012 | 0.21 |
| GO:0016779 | F | nucleotidyltransferase activity | 157 | 17430 | 553 | 52012 | 0.97 |
| GO:0016778 | F | diphosphotransferase activity | 8 | 17430 | 28 | 52012 | 0.72 |
| GO:0051539 | F | 4 iron, 4 sulfur cluster binding | 10 | 17430 | 88 | 52012 | 1 |
| GO:0016772 | F | transferase activity, transferring phosphorus-containing groups | 1794 | 17430 | 5055 | 52012 | 0.015 |
| GO:0051537 | F | 2 iron, 2 sulfur cluster binding | 13 | 17430 | 74 | 52012 | 0.99 |
| GO:0016776 | F | phosphotransferase activity, phosphate group as acceptor | 11 | 17430 | 78 | 52012 | 1 |
| GO:0008417 | F | fucosyltransferase activity | 5 | 17430 | 11 | 52012 | 0.37 |
| GO:0070566 | F | adenylyltransferase activity | 11 | 17430 | 74 | 52012 | 1 |
| GO:0015171 | F | amino acid transmembrane transporter activity | 21 | 17430 | 54 | 52012 | 0.32 |
| GO:0070568 | F | guanylyltransferase activity | 5 | 17430 | 17 | 52012 | 0.68 |
| GO:0016157 | F | sucrose synthase activity | 13 | 17430 | 25 | 52012 | 0.13 |
| GO:0015605 | F | organophosphate ester transmembrane transporter activity | 5 | 17430 | 12 | 52012 | 0.43 |
| GO:0043565 | F | sequence-specific DNA binding | 356 | 17430 | 1132 | 52012 | 0.86 |
| GO:0005048 | F | signal sequence binding | 11 | 17430 | 29 | 52012 | 0.42 |
| GO:0015211 | F | purine nucleoside transmembrane transporter activity | 5 | 17430 | 12 | 52012 | 0.43 |
| GO:0004402 | F | histone acetyltransferase activity | 15 | 17430 | 25 | 52012 | 0.056 |
| GO:0005319 | F | lipid transporter activity | 25 | 17430 | 93 | 52012 | 0.86 |
| GO:0004222 | F | metalloendopeptidase activity | 45 | 17430 | 186 | 52012 | 0.98 |
| GO:0008172 | F | S-methyltransferase activity | 6 | 17430 | 54 | 52012 | 1 |
| GO:0008170 | F | N-methyltransferase activity | 24 | 17430 | 86 | 52012 | 0.82 |
| GO:0008171 | F | O-methyltransferase activity | 45 | 17430 | 99 | 52012 | 0.056 |
| GO:0008175 | F | tRNA methyltransferase activity | 17 | 17430 | 37 | 52012 | 0.18 |
| GO:0008889 | F | glycerophosphodiester phosphodiesterase activity | 5 | 17430 | 22 | 52012 | 0.84 |
| GO:0000155 | F | phosphorelay sensor kinase activity | 20 | 17430 | 91 | 52012 | 0.97 |
| GO:0099600 | F | transmembrane receptor activity | 23 | 17430 | 80 | 52012 | 0.77 |
| GO:0008312 | F | 7S RNA binding | 7 | 17430 | 21 | 52012 | 0.58 |
| GO:0003830 | F | beta-1,4-mannosylglycoprotein 4-beta-N-acetylglucosaminyltransferase activity | 6 | 17430 | 14 | 52012 | 0.39 |
| GO:0046912 | F | transferase activity, transferring acyl groups, acyl groups converted into alkyl on transfer | 12 | 17430 | 37 | 52012 | 0.59 |
| GO:0008083 | F | growth factor activity | 13 | 17430 | 18 | 52012 | 0.03 |
| GO:0008080 | F | N-acetyltransferase activity | 54 | 17430 | 143 | 52012 | 0.25 |
| GO:0008081 | F | phosphoric diester hydrolase activity | 18 | 17430 | 131 | 52012 | 1 |
| GO:0019899 | F | enzyme binding | 51 | 17430 | 185 | 52012 | 0.91 |
| GO:0016709 | F | oxidoreductase activity, acting on paired donors, with incorporation or reduction of molecular oxygen, NAD(P)H as one donor, and incorporation of one atom of oxygen | 15 | 17430 | 72 | 52012 | 0.97 |
| GO:0016705 | F | oxidoreductase activity, acting on paired donors, with incorporation or reduction of molecular oxygen | 357 | 17430 | 1093 | 52012 | 0.67 |
| GO:0016702 | F | oxidoreductase activity, acting on single donors with incorporation of molecular oxygen, incorporation of two atoms of oxygen | 26 | 17430 | 84 | 52012 | 0.67 |
| GO:0016703 | F | oxidoreductase activity, acting on single donors with incorporation of molecular oxygen, incorporation of one atom of oxygen (internal monooxygenases or internal mixed function oxidases) | 9 | 17430 | 17 | 52012 | 0.18 |
| GO:0016701 | F | oxidoreductase activity, acting on single donors with incorporation of molecular oxygen | 40 | 17430 | 123 | 52012 | 0.6 |
| GO:0050136 | F | NADH dehydrogenase (quinone) activity | 10 | 17430 | 59 | 52012 | 0.99 |
| GO:0016881 | F | acid-amino acid ligase activity | 12 | 17430 | 374 | 52012 | 1 |
| GO:0016880 | F | acid-ammonia (or amide) ligase activity | 15 | 17430 | 29 | 52012 | 0.12 |
| GO:0016884 | F | carbon-nitrogen ligase activity, with glutamine as amido-N-donor | 30 | 17430 | 108 | 52012 | 0.84 |
| GO:0016887 | F | ATPase activity | 274 | 17430 | 1071 | 52012 | 1 |
| GO:0005092 | F | GDP-dissociation inhibitor activity | 5 | 17430 | 29 | 52012 | 0.95 |
| GO:0005094 | F | Rho GDP-dissociation inhibitor activity | 5 | 17430 | 12 | 52012 | 0.43 |
| GO:0008168 | F | methyltransferase activity | 267 | 17430 | 743 | 52012 | 0.17 |
| GO:0042625 | F | ATPase coupled ion transmembrane transporter activity | 30 | 17430 | 280 | 52012 | 1 |
| GO:0042626 | F | ATPase activity, coupled to transmembrane movement of substances | 92 | 17430 | 425 | 52012 | 1 |
| GO:0042623 | F | ATPase activity, coupled | 145 | 17430 | 829 | 52012 | 1 |
| GO:0005253 | F | anion channel activity | 16 | 17430 | 71 | 52012 | 0.95 |
| GO:0005254 | F | chloride channel activity | 11 | 17430 | 33 | 52012 | 0.56 |
| GO:0005524 | F | ATP binding | 1796 | 17430 | 6846 | 52012 | 1 |
| GO:0005525 | F | GTP binding | 272 | 17430 | 948 | 52012 | 0.99 |
| GO:0004129 | F | cytochrome-c oxidase activity | 16 | 17430 | 44 | 52012 | 0.44 |
| GO:0003824 | F | catalytic activity | 7660 | 17430 | 24009 | 52012 | 1 |
| GO:0008308 | F | voltage-gated anion channel activity | 16 | 17430 | 71 | 52012 | 0.95 |
| GO:0008094 | F | DNA-dependent ATPase activity | 22 | 17430 | 71 | 52012 | 0.66 |
| GO:0008097 | F | 5S rRNA binding | 8 | 17430 | 19 | 52012 | 0.36 |
| GO:0016838 | F | carbon-oxygen lyase activity, acting on phosphates | 51 | 17430 | 111 | 52012 | 0.04 |
| GO:0016835 | F | carbon-oxygen lyase activity | 97 | 17430 | 335 | 52012 | 0.91 |
| GO:0016836 | F | hydro-lyase activity | 34 | 17430 | 170 | 52012 | 1 |
| GO:0016837 | F | carbon-oxygen lyase activity, acting on polysaccharides | 8 | 17430 | 25 | 52012 | 0.61 |
| GO:0016830 | F | carbon-carbon lyase activity | 71 | 17430 | 235 | 52012 | 0.8 |
| GO:0016831 | F | carboxy-lyase activity | 51 | 17430 | 137 | 52012 | 0.29 |
| GO:0016832 | F | aldehyde-lyase activity | 10 | 17430 | 30 | 52012 | 0.57 |
| GO:0016833 | F | oxo-acid-lyase activity | 6 | 17430 | 22 | 52012 | 0.74 |
| GO:0008238 | F | exopeptidase activity | 58 | 17430 | 246 | 52012 | 0.99 |
| GO:0003951 | F | NAD+ kinase activity | 5 | 17430 | 24 | 52012 | 0.89 |
| GO:0003950 | F | NAD+ ADP-ribosyltransferase activity | 6 | 17430 | 39 | 52012 | 0.98 |
| GO:0008233 | F | peptidase activity | 462 | 17430 | 1440 | 52012 | 0.8 |
| GO:0003954 | F | NADH dehydrogenase activity | 10 | 17430 | 59 | 52012 | 0.99 |
| GO:0008237 | F | metallopeptidase activity | 53 | 17430 | 286 | 52012 | 1 |
| GO:0008236 | F | serine-type peptidase activity | 150 | 17430 | 515 | 52012 | 0.94 |
| GO:0051087 | F | chaperone binding | 23 | 17430 | 55 | 52012 | 0.22 |
| GO:0051082 | F | unfolded protein binding | 47 | 17430 | 465 | 52012 | 1 |
| GO:0042802 | F | identical protein binding | 6 | 17430 | 124 | 52012 | 1 |
| GO:0042803 | F | protein homodimerization activity | 6 | 17430 | 32 | 52012 | 0.94 |
| GO:0016717 | F | oxidoreductase activity, acting on paired donors, with oxidation of a pair of donors resulting in the reduction of molecular oxygen to two molecules of water | 17 | 17430 | 43 | 52012 | 0.33 |
| GO:0015116 | F | sulfate transmembrane transporter activity | 19 | 17430 | 43 | 52012 | 0.19 |
| GO:0031406 | F | carboxylic acid binding | 27 | 17430 | 197 | 52012 | 1 |
| GO:0015020 | F | glucuronosyltransferase activity | 12 | 17430 | 21 | 52012 | 0.1 |
| GO:0019842 | F | vitamin binding | 14 | 17430 | 117 | 52012 | 1 |
| GO:0019843 | F | rRNA binding | 24 | 17430 | 59 | 52012 | 0.25 |
| GO:0017069 | F | snRNA binding | 10 | 17430 | 17 | 52012 | 0.12 |
| GO:0005089 | F | Rho guanyl-nucleotide exchange factor activity | 16 | 17430 | 52 | 52012 | 0.66 |
| GO:0005088 | F | Ras guanyl-nucleotide exchange factor activity | 16 | 17430 | 52 | 52012 | 0.66 |
| GO:0005085 | F | guanyl-nucleotide exchange factor activity | 29 | 17430 | 93 | 52012 | 0.67 |
| GO:0005086 | F | ARF guanyl-nucleotide exchange factor activity | 12 | 17430 | 33 | 52012 | 0.46 |
| GO:0019829 | F | cation-transporting ATPase activity | 30 | 17430 | 163 | 52012 | 1 |
| GO:0004420 | F | hydroxymethylglutaryl-CoA reductase (NADPH) activity | 9 | 17430 | 12 | 52012 | 0.057 |
| GO:0004427 | F | inorganic diphosphatase activity | 22 | 17430 | 76 | 52012 | 0.76 |
| GO:0010277 | F | chlorophyllide a oxygenase [overall] activity | 7 | 17430 | 10 | 52012 | 0.11 |
| GO:0022803 | F | passive transmembrane transporter activity | 69 | 17430 | 217 | 52012 | 0.67 |
| GO:0022804 | F | active transmembrane transporter activity | 270 | 17430 | 976 | 52012 | 1 |
| GO:0005337 | F | nucleoside transmembrane transporter activity | 15 | 17430 | 29 | 52012 | 0.12 |
| GO:0004332 | F | fructose-bisphosphate aldolase activity | 7 | 17430 | 21 | 52012 | 0.58 |
| GO:0005247 | F | voltage-gated chloride channel activity | 11 | 17430 | 33 | 52012 | 0.56 |
| GO:0005244 | F | voltage-gated ion channel activity | 16 | 17430 | 115 | 52012 | 1 |
| GO:0016820 | F | hydrolase activity, acting on acid anhydrides, catalyzing transmembrane movement of substances | 107 | 17430 | 457 | 52012 | 1 |
| GO:0046933 | F | proton-transporting ATP synthase activity, rotational mechanism | 20 | 17430 | 78 | 52012 | 0.88 |
| GO:0016829 | F | lyase activity | 206 | 17430 | 763 | 52012 | 1 |
| GO:0004751 | F | ribose-5-phosphate isomerase activity | 6 | 17430 | 7 | 52012 | 0.082 |
| GO:0015295 | F | solute:proton symporter activity | 10 | 17430 | 35 | 52012 | 0.73 |
| GO:0015294 | F | solute:cation symporter activity | 10 | 17430 | 69 | 52012 | 1 |
| GO:0015297 | F | antiporter activity | 106 | 17430 | 271 | 52012 | 0.099 |
| GO:0015291 | F | secondary active transmembrane transporter activity | 149 | 17430 | 426 | 52012 | 0.34 |
| GO:0015293 | F | symporter activity | 10 | 17430 | 69 | 52012 | 1 |
| GO:0015299 | F | solute:proton antiporter activity | 38 | 17430 | 94 | 52012 | 0.19 |
| GO:0015298 | F | solute:cation antiporter activity | 38 | 17430 | 94 | 52012 | 0.19 |
| GO:0003968 | F | RNA-directed RNA polymerase activity | 7 | 17430 | 12 | 52012 | 0.18 |
| GO:0038023 | F | signaling receptor activity | 43 | 17430 | 154 | 52012 | 0.87 |
| GO:0008443 | F | phosphofructokinase activity | 11 | 17430 | 42 | 52012 | 0.81 |
| GO:0015103 | F | inorganic anion transmembrane transporter activity | 51 | 17430 | 110 | 52012 | 0.036 |
| GO:0019104 | F | DNA N-glycosylase activity | 11 | 17430 | 47 | 52012 | 0.89 |
| GO:0015035 | F | protein disulfide oxidoreductase activity | 97 | 17430 | 225 | 52012 | 0.023 |
| GO:0015036 | F | disulfide oxidoreductase activity | 97 | 17430 | 236 | 52012 | 0.053 |
| GO:0016211 | F | ammonia ligase activity | 15 | 17430 | 29 | 52012 | 0.12 |
| GO:0034212 | F | peptide N-acetyltransferase activity | 15 | 17430 | 25 | 52012 | 0.056 |
| GO:0030594 | F | neurotransmitter receptor activity | 21 | 17430 | 57 | 52012 | 0.4 |
| GO:0030599 | F | pectinesterase activity | 62 | 17430 | 197 | 52012 | 0.69 |
| GO:0030170 | F | pyridoxal phosphate binding | 80 | 17430 | 389 | 52012 | 1 |
| GO:0003779 | F | actin binding | 29 | 17430 | 193 | 52012 | 1 |
| GO:0043021 | F | ribonucleoprotein complex binding | 14 | 17430 | 39 | 52012 | 0.46 |
| GO:0017076 | F | purine nucleotide binding | 2320 | 17430 | 8193 | 52012 | 1 |
| GO:0043022 | F | ribosome binding | 12 | 17430 | 35 | 52012 | 0.53 |
| GO:0003774 | F | motor activity | 124 | 17430 | 345 | 52012 | 0.27 |
| GO:0003777 | F | microtubule motor activity | 92 | 17430 | 266 | 52012 | 0.42 |
| GO:0016725 | F | oxidoreductase activity, acting on CH or CH2 groups | 6 | 17430 | 13 | 52012 | 0.34 |
| GO:0004435 | F | phosphatidylinositol phospholipase C activity | 6 | 17430 | 28 | 52012 | 0.89 |
| GO:0035091 | F | phosphatidylinositol binding | 14 | 17430 | 99 | 52012 | 1 |
| GO:0030570 | F | pectate lyase activity | 8 | 17430 | 25 | 52012 | 0.61 |
| GO:0005346 | F | purine ribonucleotide transmembrane transporter activity | 5 | 17430 | 12 | 52012 | 0.43 |
| GO:0005347 | F | ATP transmembrane transporter activity | 5 | 17430 | 12 | 52012 | 0.43 |
| GO:0005342 | F | organic acid transmembrane transporter activity | 21 | 17430 | 86 | 52012 | 0.93 |
| GO:0008146 | F | sulfotransferase activity | 30 | 17430 | 64 | 52012 | 0.082 |
| GO:2001070 | F | starch binding | 6 | 17430 | 41 | 52012 | 0.99 |
| GO:0000166 | F | nucleotide binding | 2571 | 17430 | 11390 | 52012 | 1 |
| GO:0004325 | F | ferrochelatase activity | 6 | 17430 | 19 | 52012 | 0.63 |
| GO:0005509 | F | calcium ion binding | 115 | 17430 | 778 | 52012 | 1 |
| GO:0004106 | F | chorismate mutase activity | 5 | 17430 | 21 | 52012 | 0.82 |
| GO:0008324 | F | cation transmembrane transporter activity | 258 | 17430 | 825 | 52012 | 0.84 |
| GO:0046923 | F | ER retention sequence binding | 11 | 17430 | 27 | 52012 | 0.35 |
| GO:0016854 | F | racemase and epimerase activity | 8 | 17430 | 70 | 52012 | 1 |
| GO:0016853 | F | isomerase activity | 155 | 17430 | 602 | 52012 | 1 |
| GO:0031420 | F | alkali metal ion binding | 19 | 17430 | 50 | 52012 | 0.36 |
| GO:0016859 | F | cis-trans isomerase activity | 41 | 17430 | 119 | 52012 | 0.47 |
| GO:0004749 | F | ribose phosphate diphosphokinase activity | 5 | 17430 | 17 | 52012 | 0.68 |
| GO:0004743 | F | pyruvate kinase activity | 19 | 17430 | 50 | 52012 | 0.36 |
| GO:0001883 | F | purine nucleoside binding | 2307 | 17430 | 8163 | 52012 | 1 |
| GO:0001882 | F | nucleoside binding | 2322 | 17430 | 8191 | 52012 | 1 |
| GO:0010181 | F | FMN binding | 11 | 17430 | 148 | 52012 | 1 |
| GO:0044769 | F | ATPase activity, coupled to transmembrane movement of ions, rotational mechanism | 29 | 17430 | 103 | 52012 | 0.82 |
| GO:0017089 | F | glycolipid transporter activity | 6 | 17430 | 14 | 52012 | 0.39 |
| GO:0070011 | F | peptidase activity, acting on L-amino acid peptides | 436 | 17430 | 1379 | 52012 | 0.86 |
| GO:0019139 | F | cytokinin dehydrogenase activity | 12 | 17430 | 22 | 52012 | 0.12 |
| GO:0047325 | F | inositol tetrakisphosphate 1-kinase activity | 7 | 17430 | 28 | 52012 | 0.81 |
| GO:0016597 | F | amino acid binding | 27 | 17430 | 138 | 52012 | 1 |
| GO:0016616 | F | oxidoreductase activity, acting on the CH-OH group of donors, NAD or NADP as acceptor | 162 | 17430 | 477 | 52012 | 0.46 |
| GO:0015002 | F | heme-copper terminal oxidase activity | 16 | 17430 | 44 | 52012 | 0.44 |
| GO:0016209 | F | antioxidant activity | 126 | 17430 | 327 | 52012 | 0.1 |
| GO:0033926 | F | glycopeptide alpha-N-acetylgalactosaminidase activity | 13 | 17430 | 34 | 52012 | 0.4 |
| GO:0019825 | F | oxygen binding | 5 | 17430 | 8 | 52012 | 0.21 |
| GO:1901677 | F | phosphate transmembrane transporter activity | 7 | 17430 | 27 | 52012 | 0.79 |
| GO:0034062 | F | RNA polymerase activity | 79 | 17430 | 212 | 52012 | 0.23 |
| GO:0034061 | F | DNA polymerase activity | 15 | 17430 | 78 | 52012 | 0.99 |
| GO:0030145 | F | manganese ion binding | 9 | 17430 | 115 | 52012 | 1 |
| GO:0003746 | F | translation elongation factor activity | 21 | 17430 | 100 | 52012 | 0.98 |
| GO:0003747 | F | translation release factor activity | 12 | 17430 | 29 | 52012 | 0.32 |
| GO:0003743 | F | translation initiation factor activity | 60 | 17430 | 127 | 52012 | 0.019 |
| GO:0022824 | F | transmitter-gated ion channel activity | 21 | 17430 | 57 | 52012 | 0.4 |
| GO:0005351 | F | sugar:proton symporter activity | 10 | 17430 | 35 | 52012 | 0.73 |
| GO:0008138 | F | protein tyrosine/serine/threonine phosphatase activity | 26 | 17430 | 90 | 52012 | 0.78 |
| GO:0008137 | F | NADH dehydrogenase (ubiquinone) activity | 10 | 17430 | 59 | 52012 | 0.99 |
| GO:0008134 | F | transcription factor binding | 6 | 17430 | 18 | 52012 | 0.58 |
| GO:0008135 | F | translation factor activity, RNA binding | 93 | 17430 | 256 | 52012 | 0.27 |
| GO:0008131 | F | primary amine oxidase activity | 7 | 17430 | 32 | 52012 | 0.89 |
| GO:0004356 | F | glutamate-ammonia ligase activity | 15 | 17430 | 29 | 52012 | 0.12 |
| GO:0005516 | F | calmodulin binding | 32 | 17430 | 53 | 52012 | 0.007 |
| GO:0005515 | F | protein binding | 3243 | 17430 | 12774 | 52012 | 1 |
| GO:0004176 | F | ATP-dependent peptidase activity | 10 | 17430 | 25 | 52012 | 0.38 |
| GO:0004177 | F | aminopeptidase activity | 11 | 17430 | 68 | 52012 | 0.99 |
| GO:0004175 | F | endopeptidase activity | 272 | 17430 | 963 | 52012 | 1 |
| GO:0016844 | F | strictosidine synthase activity | 7 | 17430 | 32 | 52012 | 0.89 |
| GO:0016846 | F | carbon-sulfur lyase activity | 11 | 17430 | 97 | 52012 | 1 |
| GO:0016840 | F | carbon-nitrogen lyase activity | 9 | 17430 | 54 | 52012 | 0.99 |
| GO:0016843 | F | amine-lyase activity | 7 | 17430 | 32 | 52012 | 0.89 |
| GO:0000062 | F | fatty-acyl-CoA binding | 10 | 17430 | 33 | 52012 | 0.67 |
| GO:0004003 | F | ATP-dependent DNA helicase activity | 14 | 17430 | 29 | 52012 | 0.17 |
| GO:0016903 | F | oxidoreductase activity, acting on the aldehyde or oxo group of donors | 43 | 17430 | 190 | 52012 | 0.99 |
| GO:0004635 | F | phosphoribosyl-AMP cyclohydrolase activity | 5 | 17430 | 21 | 52012 | 0.82 |
| GO:0008661 | F | 1-deoxy-D-xylulose-5-phosphate synthase activity | 6 | 17430 | 16 | 52012 | 0.49 |
| GO:0070001 | F | aspartic-type peptidase activity | 83 | 17430 | 212 | 52012 | 0.13 |
| GO:0070003 | F | threonine-type peptidase activity | 35 | 17430 | 106 | 52012 | 0.56 |
| GO:0070008 | F | serine-type exopeptidase activity | 41 | 17430 | 140 | 52012 | 0.8 |
| GO:0019901 | F | protein kinase binding | 12 | 17430 | 130 | 52012 | 1 |
| GO:0019900 | F | kinase binding | 12 | 17430 | 130 | 52012 | 1 |
| GO:0019904 | F | protein domain specific binding | 24 | 17430 | 56 | 52012 | 0.19 |
| GO:0052725 | F | inositol-1,3,4-trisphosphate 6-kinase activity | 7 | 17430 | 28 | 52012 | 0.81 |
| GO:0015018 | F | galactosylgalactosylxylosylprotein 3-beta-glucuronosyltransferase activity | 10 | 17430 | 21 | 52012 | 0.23 |
| GO:0016278 | F | lysine N-methyltransferase activity | 18 | 17430 | 66 | 52012 | 0.82 |
| GO:0016279 | F | protein-lysine N-methyltransferase activity | 18 | 17430 | 66 | 52012 | 0.82 |
| GO:0030976 | F | thiamine pyrophosphate binding | 11 | 17430 | 40 | 52012 | 0.77 |
| GO:0035251 | F | UDP-glucosyltransferase activity | 52 | 17430 | 209 | 52012 | 0.98 |
| GO:0016307 | F | phosphatidylinositol phosphate kinase activity | 20 | 17430 | 59 | 52012 | 0.53 |
| GO:0008601 | F | protein phosphatase type 2A regulator activity | 34 | 17430 | 80 | 52012 | 0.15 |
| GO:0030151 | F | molybdenum ion binding | 8 | 17430 | 24 | 52012 | 0.57 |
| GO:0042277 | F | peptide binding | 11 | 17430 | 29 | 52012 | 0.42 |
| GO:0003755 | F | peptidyl-prolyl cis-trans isomerase activity | 41 | 17430 | 119 | 52012 | 0.47 |
| GO:0009982 | F | pseudouridine synthase activity | 16 | 17430 | 50 | 52012 | 0.61 |
| GO:0030554 | F | adenyl nucleotide binding | 2041 | 17430 | 7245 | 52012 | 1 |
| GO:0022832 | F | voltage-gated channel activity | 16 | 17430 | 115 | 52012 | 1 |
| GO:0022836 | F | gated channel activity | 46 | 17430 | 175 | 52012 | 0.94 |
| GO:0022835 | F | transmitter-gated channel activity | 21 | 17430 | 57 | 52012 | 0.4 |
| GO:0022834 | F | ligand-gated channel activity | 21 | 17430 | 60 | 52012 | 0.47 |
| GO:0022838 | F | substrate-specific channel activity | 69 | 17430 | 217 | 52012 | 0.67 |
| GO:0051536 | F | iron-sulfur cluster binding | 69 | 17430 | 249 | 52012 | 0.93 |
| GO:0016775 | F | phosphotransferase activity, nitrogenous group as acceptor | 20 | 17430 | 91 | 52012 | 0.97 |
| GO:0045156 | F | electron transporter, transferring electrons within the cyclic electron transport pathway of photosynthesis activity | 9 | 17430 | 17 | 52012 | 0.18 |
| GO:0004345 | F | glucose-6-phosphate dehydrogenase activity | 7 | 17430 | 26 | 52012 | 0.76 |
| GO:0010333 | F | terpene synthase activity | 47 | 17430 | 90 | 52012 | 0.01 |
| GO:0015077 | F | monovalent inorganic cation transmembrane transporter activity | 161 | 17430 | 545 | 52012 | 0.93 |
| GO:0016671 | F | oxidoreductase activity, acting on a sulfur group of donors, disulfide as acceptor | 11 | 17430 | 32 | 52012 | 0.53 |
| GO:0032559 | F | adenyl ribonucleotide binding | 2035 | 17430 | 7232 | 52012 | 1 |
| GO:0090484 | F | drug transporter activity | 63 | 17430 | 165 | 52012 | 0.21 |
| GO:0016879 | F | ligase activity, forming carbon-nitrogen bonds | 75 | 17430 | 599 | 52012 | 1 |
| GO:0016874 | F | ligase activity | 171 | 17430 | 989 | 52012 | 1 |
| GO:0046943 | F | carboxylic acid transmembrane transporter activity | 21 | 17430 | 86 | 52012 | 0.93 |
| GO:0009055 | F | electron carrier activity | 168 | 17430 | 1056 | 52012 | 1 |
| GO:0051287 | F | NAD binding | 64 | 17430 | 234 | 52012 | 0.94 |
| GO:0004012 | F | phospholipid-translocating ATPase activity | 18 | 17430 | 43 | 52012 | 0.25 |
| GO:0004014 | F | adenosylmethionine decarboxylase activity | 7 | 17430 | 8 | 52012 | 0.058 |
| GO:0003917 | F | DNA topoisomerase type I activity | 6 | 17430 | 17 | 52012 | 0.54 |
| GO:0008271 | F | secondary active sulfate transmembrane transporter activity | 19 | 17430 | 43 | 52012 | 0.19 |
| GO:0008270 | F | zinc ion binding | 871 | 17430 | 4422 | 52012 | 1 |
| GO:0003918 | F | DNA topoisomerase type II (ATP-hydrolyzing) activity | 6 | 17430 | 17 | 52012 | 0.54 |
| GO:0045300 | F | acyl-[acyl-carrier-protein] desaturase activity | 9 | 17430 | 11 | 52012 | 0.042 |
| GO:0070035 | F | purine NTP-dependent helicase activity | 15 | 17430 | 326 | 52012 | 1 |
| GO:1901476 | F | carbohydrate transporter activity | 13 | 17430 | 48 | 52012 | 0.79 |
| GO:0032549 | F | ribonucleoside binding | 2321 | 17430 | 8189 | 52012 | 1 |
| GO:0016684 | F | oxidoreductase activity, acting on peroxide as acceptor | 111 | 17430 | 262 | 52012 | 0.023 |
| GO:0016682 | F | oxidoreductase activity, acting on diphenols and related substances as donors, oxygen as acceptor | 10 | 17430 | 74 | 52012 | 1 |
| GO:0051861 | F | glycolipid binding | 6 | 17430 | 14 | 52012 | 0.39 |
| GO:0051765 | F | inositol tetrakisphosphate kinase activity | 7 | 17430 | 28 | 52012 | 0.81 |
| GO:0051766 | F | inositol trisphosphate kinase activity | 10 | 17430 | 34 | 52012 | 0.7 |
| GO:0043492 | F | ATPase activity, coupled to movement of substances | 110 | 17430 | 425 | 52012 | 0.99 |
| GO:0016462 | F | pyrophosphatase activity | 559 | 17430 | 2710 | 52012 | 1 |
| GO:0003905 | F | alkylbase DNA N-glycosylase activity | 9 | 17430 | 36 | 52012 | 0.83 |
| GO:1901505 | F | carbohydrate derivative transporter activity | 21 | 17430 | 56 | 52012 | 0.37 |
| GO:0015932 | F | nucleobase-containing compound transmembrane transporter activity | 15 | 17430 | 42 | 52012 | 0.47 |
| GO:0072509 | F | divalent inorganic cation transmembrane transporter activity | 14 | 17430 | 74 | 52012 | 0.99 |
| GO:0030414 | F | peptidase inhibitor activity | 22 | 17430 | 38 | 52012 | 0.031 |
| GO:0003723 | F | RNA binding | 388 | 17430 | 1378 | 52012 | 1 |
| GO:0036094 | F | small molecule binding | 2597 | 17430 | 11530 | 52012 | 1 |
| GO:0004970 | F | ionotropic glutamate receptor activity | 21 | 17430 | 57 | 52012 | 0.4 |
| GO:0005198 | F | structural molecule activity | 510 | 17430 | 1500 | 52012 | 0.4 |
| GO:0008113 | F | peptide-methionine (S)-S-oxide reductase activity | 5 | 17430 | 10 | 52012 | 0.32 |
| GO:0004807 | F | triose-phosphate isomerase activity | 5 | 17430 | 9 | 52012 | 0.26 |
| GO:0004809 | F | tRNA (guanine-N2-)-methyltransferase activity | 5 | 17430 | 8 | 52012 | 0.21 |
| GO:0004571 | F | mannosyl-oligosaccharide 1,2-alpha-mannosidase activity | 6 | 17430 | 23 | 52012 | 0.77 |
| GO:0004576 | F | oligosaccharyl transferase activity | 10 | 17430 | 50 | 52012 | 0.96 |
| GO:0004575 | F | sucrose alpha-glucosidase activity | 5 | 17430 | 15 | 52012 | 0.59 |
| GO:0004579 | F | dolichyl-diphosphooligosaccharide-protein glycotransferase activity | 6 | 17430 | 39 | 52012 | 0.98 |
| GO:0004396 | F | hexokinase activity | 8 | 17430 | 16 | 52012 | 0.24 |
| GO:0008066 | F | glutamate receptor activity | 21 | 17430 | 57 | 52012 | 0.4 |
| GO:0008061 | F | chitin binding | 9 | 17430 | 13 | 52012 | 0.076 |
| GO:0016868 | F | intramolecular transferase activity, phosphotransferases | 15 | 17430 | 61 | 52012 | 0.89 |
| GO:0016866 | F | intramolecular transferase activity | 48 | 17430 | 199 | 52012 | 0.99 |
| GO:0016861 | F | intramolecular oxidoreductase activity, interconverting aldoses and ketoses | 19 | 17430 | 46 | 52012 | 0.26 |
| GO:0016860 | F | intramolecular oxidoreductase activity | 23 | 17430 | 52 | 52012 | 0.16 |
| GO:0005375 | F | copper ion transmembrane transporter activity | 10 | 17430 | 19 | 52012 | 0.17 |
| GO:0003924 | F | GTPase activity | 76 | 17430 | 610 | 52012 | 1 |
| GO:0022891 | F | substrate-specific transmembrane transporter activity | 412 | 17430 | 1510 | 52012 | 1 |
| GO:0004629 | F | phospholipase C activity | 6 | 17430 | 55 | 52012 | 1 |
| GO:0004620 | F | phospholipase activity | 9 | 17430 | 97 | 52012 | 1 |
| GO:0008483 | F | transaminase activity | 20 | 17430 | 95 | 52012 | 0.98 |
| GO:1901265 | F | nucleoside phosphate binding | 2571 | 17430 | 11390 | 52012 | 1 |
| GO:0008641 | F | small protein activating enzyme activity | 17 | 17430 | 24 | 52012 | 0.016 |
| GO:0016787 | F | hydrolase activity | 2247 | 17430 | 8107 | 52012 | 1 |
| GO:0016782 | F | transferase activity, transferring sulfur-containing groups | 34 | 17430 | 92 | 52012 | 0.34 |
| GO:0016780 | F | phosphotransferase activity, for other substituted phosphate groups | 13 | 17430 | 40 | 52012 | 0.59 |
| GO:0016788 | F | hydrolase activity, acting on ester bonds | 531 | 17430 | 2096 | 52012 | 1 |
| GO:0015075 | F | ion transmembrane transporter activity | 367 | 17430 | 1175 | 52012 | 0.89 |
| GO:0016676 | F | oxidoreductase activity, acting on a heme group of donors, oxygen as acceptor | 16 | 17430 | 44 | 52012 | 0.44 |
| GO:0016675 | F | oxidoreductase activity, acting on a heme group of donors | 16 | 17430 | 44 | 52012 | 0.44 |
| GO:0032555 | F | purine ribonucleotide binding | 2307 | 17430 | 8163 | 52012 | 1 |
| GO:0016679 | F | oxidoreductase activity, acting on diphenols and related substances as donors | 18 | 17430 | 107 | 52012 | 1 |
| GO:0015079 | F | potassium ion transmembrane transporter activity | 25 | 17430 | 138 | 52012 | 1 |
| GO:0015078 | F | hydrogen ion transmembrane transporter activity | 136 | 17430 | 375 | 52012 | 0.23 |
| GO:0032553 | F | ribonucleotide binding | 2318 | 17430 | 8311 | 52012 | 1 |
| GO:1901681 | F | sulfur compound binding | 12 | 17430 | 33 | 52012 | 0.46 |
| GO:0030955 | F | potassium ion binding | 19 | 17430 | 50 | 52012 | 0.36 |
| GO:1901682 | F | sulfur compound transmembrane transporter activity | 19 | 17430 | 43 | 52012 | 0.19 |
| GO:0016410 | F | N-acyltransferase activity | 58 | 17430 | 150 | 52012 | 0.2 |
| GO:0016411 | F | acylglycerol O-acyltransferase activity | 13 | 17430 | 33 | 52012 | 0.36 |
| GO:0015926 | F | glucosidase activity | 5 | 17430 | 19 | 52012 | 0.76 |
| GO:0015924 | F | mannosyl-oligosaccharide mannosidase activity | 6 | 17430 | 23 | 52012 | 0.77 |
| GO:0015923 | F | mannosidase activity | 16 | 17430 | 44 | 52012 | 0.44 |
| GO:0032550 | F | purine ribonucleoside binding | 2307 | 17430 | 8163 | 52012 | 1 |
| GO:0004721 | F | phosphoprotein phosphatase activity | 147 | 17430 | 367 | 52012 | 0.038 |
| GO:0016641 | F | oxidoreductase activity, acting on the CH-NH2 group of donors, oxygen as acceptor | 8 | 17430 | 39 | 52012 | 0.93 |
| GO:0022857 | F | transmembrane transporter activity | 677 | 17430 | 1956 | 52012 | 0.24 |
| GO:0022853 | F | active ion transmembrane transporter activity | 30 | 17430 | 283 | 52012 | 1 |
| GO:0004497 | F | monooxygenase activity | 24 | 17430 | 153 | 52012 | 1 |
| GO:0004499 | F | N,N-dimethylaniline monooxygenase activity | 12 | 17430 | 34 | 52012 | 0.49 |
| GO:0004367 | F | glycerol-3-phosphate dehydrogenase [NAD+] activity | 5 | 17430 | 25 | 52012 | 0.9 |
| GO:0004812 | F | aminoacyl-tRNA ligase activity | 66 | 17430 | 235 | 52012 | 0.91 |
| GO:0004814 | F | arginine-tRNA ligase activity | 6 | 17430 | 27 | 52012 | 0.87 |
| GO:0005543 | F | phospholipid binding | 49 | 17430 | 305 | 52012 | 1 |
| GO:0005544 | F | calcium-dependent phospholipid binding | 11 | 17430 | 36 | 52012 | 0.66 |
| GO:0052726 | F | inositol-1,3,4-trisphosphate 5-kinase activity | 7 | 17430 | 28 | 52012 | 0.81 |
| GO:0004568 | F | chitinase activity | 15 | 17430 | 37 | 52012 | 0.31 |
| GO:0008079 | F | translation termination factor activity | 12 | 17430 | 29 | 52012 | 0.32 |
| GO:0051119 | F | sugar transmembrane transporter activity | 13 | 17430 | 48 | 52012 | 0.79 |
| GO:0004386 | F | helicase activity | 77 | 17430 | 522 | 52012 | 1 |
| GO:0016892 | F | endoribonuclease activity, producing 3'-phosphomonoesters | 14 | 17430 | 38 | 52012 | 0.43 |
| GO:0016893 | F | endonuclease activity, active with either ribo- or deoxyribonucleic acids and producing 5'-phosphomonoesters | 20 | 17430 | 84 | 52012 | 0.94 |
| GO:0016891 | F | endoribonuclease activity, producing 5'-phosphomonoesters | 16 | 17430 | 76 | 52012 | 0.97 |
| GO:0016894 | F | endonuclease activity, active with either ribo- or deoxyribonucleic acids and producing 3'-phosphomonoesters | 14 | 17430 | 38 | 52012 | 0.43 |
| GO:0018024 | F | histone-lysine N-methyltransferase activity | 18 | 17430 | 66 | 52012 | 0.82 |
| GO:0009678 | F | hydrogen-translocating pyrophosphatase activity | 11 | 17430 | 38 | 52012 | 0.72 |
| GO:0004144 | F | diacylglycerol O-acyltransferase activity | 13 | 17430 | 28 | 52012 | 0.21 |
| GO:0004143 | F | diacylglycerol kinase activity | 8 | 17430 | 42 | 52012 | 0.96 |
| GO:0004634 | F | phosphopyruvate hydratase activity | 5 | 17430 | 23 | 52012 | 0.87 |
| GO:0016759 | F | cellulose synthase activity | 20 | 17430 | 81 | 52012 | 0.91 |
| GO:0008276 | F | protein methyltransferase activity | 22 | 17430 | 100 | 52012 | 0.98 |
| GO:0019001 | F | guanyl nucleotide binding | 279 | 17430 | 965 | 52012 | 0.99 |
| GO:0015267 | F | channel activity | 69 | 17430 | 217 | 52012 | 0.67 |
| GO:0046983 | F | protein dimerization activity | 328 | 17430 | 1009 | 52012 | 0.7 |
| GO:0016791 | F | phosphatase activity | 185 | 17430 | 622 | 52012 | 0.93 |
| GO:0016790 | F | thiolester hydrolase activity | 14 | 17430 | 67 | 52012 | 0.96 |
| GO:0016799 | F | hydrolase activity, hydrolyzing N-glycosyl compounds | 14 | 17430 | 51 | 52012 | 0.79 |
| GO:0016798 | F | hydrolase activity, acting on glycosyl bonds | 384 | 17430 | 1048 | 52012 | 0.07 |
| GO:0032561 | F | guanyl ribonucleotide binding | 272 | 17430 | 948 | 52012 | 0.99 |
| GO:0033218 | F | amide binding | 12 | 17430 | 47 | 52012 | 0.84 |
| GO:0016407 | F | acetyltransferase activity | 58 | 17430 | 153 | 52012 | 0.23 |
| GO:0030983 | F | mismatched DNA binding | 15 | 17430 | 44 | 52012 | 0.53 |
| GO:0016538 | F | cyclin-dependent protein serine/threonine kinase regulator activity | 15 | 17430 | 25 | 52012 | 0.056 |
| GO:0061135 | F | endopeptidase regulator activity | 22 | 17430 | 38 | 52012 | 0.031 |
| GO:0061134 | F | peptidase regulator activity | 22 | 17430 | 38 | 52012 | 0.031 |
| GO:0017111 | F | nucleoside-triphosphatase activity | 536 | 17430 | 2615 | 52012 | 1 |
| GO:0060089 | F | molecular transducer activity | 43 | 17430 | 159 | 52012 | 0.91 |
| GO:0003713 | F | transcription coactivator activity | 5 | 17430 | 8 | 52012 | 0.21 |
| GO:0004827 | F | proline-tRNA ligase activity | 6 | 17430 | 19 | 52012 | 0.63 |
| GO:0004556 | F | alpha-amylase activity | 7 | 17430 | 19 | 52012 | 0.49 |
| GO:0004550 | F | nucleoside diphosphate kinase activity | 5 | 17430 | 10 | 52012 | 0.32 |
| GO:0004553 | F | hydrolase activity, hydrolyzing O-glycosyl compounds | 360 | 17430 | 963 | 52012 | 0.04 |
| GO:0004559 | F | alpha-mannosidase activity | 12 | 17430 | 43 | 52012 | 0.76 |
| GO:0005548 | F | phospholipid transporter activity | 18 | 17430 | 43 | 52012 | 0.25 |
| GO:0004045 | F | aminoacyl-tRNA hydrolase activity | 8 | 17430 | 43 | 52012 | 0.96 |
| GO:0004601 | F | peroxidase activity | 109 | 17430 | 259 | 52012 | 0.027 |
| GO:0004602 | F | glutathione peroxidase activity | 8 | 17430 | 26 | 52012 | 0.65 |
| GO:0008509 | F | anion transmembrane transporter activity | 56 | 17430 | 156 | 52012 | 0.35 |
| GO:0015276 | F | ligand-gated ion channel activity | 21 | 17430 | 60 | 52012 | 0.47 |
| GO:0004564 | F | beta-fructofuranosidase activity | 5 | 17430 | 15 | 52012 | 0.59 |
| GO:0017171 | F | serine hydrolase activity | 150 | 17430 | 515 | 52012 | 0.94 |
| GO:0016655 | F | oxidoreductase activity, acting on NAD(P)H, quinone or similar compound as acceptor | 14 | 17430 | 73 | 52012 | 0.98 |
| GO:0016651 | F | oxidoreductase activity, acting on NAD(P)H | 113 | 17430 | 275 | 52012 | 0.04 |
| GO:0042054 | F | histone methyltransferase activity | 18 | 17430 | 66 | 52012 | 0.82 |
| GO:0090599 | F | alpha-glucosidase activity | 5 | 17430 | 15 | 52012 | 0.59 |
| GO:0008173 | F | RNA methyltransferase activity | 30 | 17430 | 86 | 52012 | 0.46 |
| GO:0046961 | F | proton-transporting ATPase activity, rotational mechanism | 26 | 17430 | 97 | 52012 | 0.87 |
| GO:0001076 | F | transcription factor activity, RNA polymerase II transcription factor binding | 15 | 17430 | 54 | 52012 | 0.78 |
| GO:0001071 | F | nucleic acid binding transcription factor activity | 647 | 17430 | 1741 | 52012 | 0.012 |
| GO:0003712 | F | transcription cofactor activity | 49 | 17430 | 116 | 52012 | 0.1 |
| GO:0097367 | F | carbohydrate derivative binding | 2348 | 17430 | 8366 | 52012 | 1 |
| GO:0015108 | F | chloride transmembrane transporter activity | 11 | 17430 | 35 | 52012 | 0.63 |
| GO:0004540 | F | ribonuclease activity | 39 | 17430 | 158 | 52012 | 0.97 |
| GO:0005230 | F | extracellular ligand-gated ion channel activity | 21 | 17430 | 60 | 52012 | 0.47 |
| GO:0005231 | F | excitatory extracellular ligand-gated ion channel activity | 7 | 17430 | 57 | 52012 | 1 |
| GO:0005234 | F | extracellular-glutamate-gated ion channel activity | 7 | 17430 | 57 | 52012 | 1 |
| GO:0008519 | F | ammonium transmembrane transporter activity | 10 | 17430 | 17 | 52012 | 0.12 |
| GO:0003916 | F | DNA topoisomerase activity | 10 | 17430 | 25 | 52012 | 0.38 |
| GO:0004611 | F | phosphoenolpyruvate carboxykinase activity | 10 | 17430 | 25 | 52012 | 0.38 |
| GO:0004616 | F | phosphogluconate dehydrogenase (decarboxylating) activity | 13 | 17430 | 34 | 52012 | 0.4 |
| GO:0004619 | F | phosphoglycerate mutase activity | 7 | 17430 | 8 | 52012 | 0.058 |
| GO:0032296 | F | double-stranded RNA-specific ribonuclease activity | 13 | 17430 | 41 | 52012 | 0.62 |
| GO:0052689 | F | carboxylic ester hydrolase activity | 80 | 17430 | 386 | 52012 | 1 |
| GO:0050308 | F | sugar-phosphatase activity | 9 | 17430 | 28 | 52012 | 0.61 |
| GO:0019203 | F | carbohydrate phosphatase activity | 9 | 17430 | 28 | 52012 | 0.61 |
| GO:0019200 | F | carbohydrate kinase activity | 19 | 17430 | 90 | 52012 | 0.98 |
| GO:0019201 | F | nucleotide kinase activity | 6 | 17430 | 64 | 52012 | 1 |
| GO:0019207 | F | kinase regulator activity | 23 | 17430 | 67 | 52012 | 0.5 |
| GO:0019205 | F | nucleobase-containing compound kinase activity | 23 | 17430 | 95 | 52012 | 0.94 |
| GO:0019208 | F | phosphatase regulator activity | 37 | 17430 | 92 | 52012 | 0.2 |
| GO:0043733 | F | DNA-3-methylbase glycosylase activity | 8 | 17430 | 34 | 52012 | 0.86 |
| GO:0016646 | F | oxidoreductase activity, acting on the CH-NH group of donors, NAD or NADP as acceptor | 10 | 17430 | 42 | 52012 | 0.87 |
| GO:0016645 | F | oxidoreductase activity, acting on the CH-NH group of donors | 26 | 17430 | 74 | 52012 | 0.46 |
| GO:0046914 | F | transition metal ion binding | 1344 | 17430 | 5835 | 52012 | 1 |
| GO:0016429 | F | tRNA (adenine-N1-)-methyltransferase activity | 9 | 17430 | 15 | 52012 | 0.12 |
| GO:0016426 | F | tRNA (adenine) methyltransferase activity | 9 | 17430 | 15 | 52012 | 0.12 |
| GO:0046915 | F | transition metal ion transmembrane transporter activity | 13 | 17430 | 48 | 52012 | 0.79 |
| GO:0016423 | F | tRNA (guanine) methyltransferase activity | 8 | 17430 | 19 | 52012 | 0.36 |
| GO:0005856 | C | cytoskeleton | 245 | 17430 | 322 | 52012 | 3.2E-21 |
| GO:0043232 | C | intracellular non-membrane-bounded organelle | 861 | 17430 | 1875 | 52012 | 1.1E-14 |
| GO:0043228 | C | non-membrane-bounded organelle | 861 | 17430 | 1875 | 52012 | 1.1E-14 |
| GO:0044446 | C | intracellular organelle part | 749 | 17430 | 1677 | 52012 | 3.7E-11 |
| GO:0044422 | C | organelle part | 749 | 17430 | 1680 | 52012 | 4.8E-11 |
| GO:0044425 | C | membrane part | 1355 | 17430 | 3420 | 52012 | 4.9E-08 |
| GO:0031224 | C | intrinsic component of membrane | 1042 | 17430 | 2581 | 52012 | 1.5E-07 |
| GO:0016021 | C | integral component of membrane | 1025 | 17430 | 2545 | 52012 | 2.6E-07 |
| GO:0032991 | C | macromolecular complex | 1461 | 17430 | 3839 | 52012 | 0.000011 |
| GO:0099512 | C | supramolecular fiber | 51 | 17430 | 70 | 52012 | 0.00003 |
| GO:0099513 | C | polymeric cytoskeletal fiber | 51 | 17430 | 70 | 52012 | 0.00003 |
| GO:0044436 | C | thylakoid part | 103 | 17430 | 188 | 52012 | 0.000058 |
| GO:0009579 | C | thylakoid | 103 | 17430 | 188 | 52012 | 0.000058 |
| GO:0009521 | C | photosystem | 87 | 17430 | 152 | 52012 | 0.000068 |
| GO:0015629 | C | actin cytoskeleton | 69 | 17430 | 116 | 52012 | 0.00016 |
| GO:0005874 | C | microtubule | 47 | 17430 | 70 | 52012 | 0.00024 |
| GO:0005739 | C | mitochondrion | 137 | 17430 | 282 | 52012 | 0.00028 |
| GO:0098800 | C | inner mitochondrial membrane protein complex | 33 | 17430 | 45 | 52012 | 0.00066 |
| GO:0030529 | C | intracellular ribonucleoprotein complex | 524 | 17430 | 1328 | 52012 | 0.00082 |
| GO:1990904 | C | ribonucleoprotein complex | 524 | 17430 | 1328 | 52012 | 0.00082 |
| GO:0071944 | C | cell periphery | 177 | 17430 | 395 | 52012 | 0.00089 |
| GO:0044427 | C | chromosomal part | 109 | 17430 | 224 | 52012 | 0.001 |
| GO:0009523 | C | photosystem II | 47 | 17430 | 77 | 52012 | 0.0011 |
| GO:0031011 | C | Ino80 complex | 17 | 17430 | 17 | 52012 | 0.0015 |
| GO:0070603 | C | SWI/SNF superfamily-type complex | 17 | 17430 | 17 | 52012 | 0.0015 |
| GO:0033202 | C | DNA helicase complex | 17 | 17430 | 17 | 52012 | 0.0015 |
| GO:0044454 | C | nuclear chromosome part | 25 | 17430 | 32 | 52012 | 0.0015 |
| GO:0097346 | C | INO80-type complex | 17 | 17430 | 17 | 52012 | 0.0015 |
| GO:0000790 | C | nuclear chromatin | 17 | 17430 | 17 | 52012 | 0.0015 |
| GO:0099023 | C | tethring complex | 36 | 17430 | 54 | 52012 | 0.0013 |
| GO:0034357 | C | photosynthetic membrane | 91 | 17430 | 185 | 52012 | 0.002 |
| GO:0043234 | C | protein complex | 939 | 17430 | 2520 | 52012 | 0.0025 |
| GO:0005840 | C | ribosome | 475 | 17430 | 1220 | 52012 | 0.0029 |
| GO:0005618 | C | cell wall | 100 | 17430 | 215 | 52012 | 0.0046 |
| GO:0030312 | C | external encapsulating structure | 100 | 17430 | 216 | 52012 | 0.0051 |
| GO:0000145 | C | exocyst | 31 | 17430 | 50 | 52012 | 0.006 |
| GO:0044428 | C | nuclear part | 174 | 17430 | 411 | 52012 | 0.006 |
| GO:0044448 | C | cell cortex part | 31 | 17430 | 50 | 52012 | 0.006 |
| GO:0009522 | C | photosystem I | 33 | 17430 | 55 | 52012 | 0.0067 |
| GO:0031981 | C | nuclear lumen | 110 | 17430 | 249 | 52012 | 0.01 |
| GO:0031974 | C | membrane-enclosed lumen | 120 | 17430 | 282 | 52012 | 0.017 |
| GO:0070013 | C | intracellular organelle lumen | 120 | 17430 | 282 | 52012 | 0.017 |
| GO:0043233 | C | organelle lumen | 120 | 17430 | 282 | 52012 | 0.017 |
| GO:0005654 | C | nucleoplasm | 70 | 17430 | 153 | 52012 | 0.02 |
| GO:0044451 | C | nucleoplasm part | 70 | 17430 | 153 | 52012 | 0.02 |
| GO:0099568 | C | cytoplasmic region | 31 | 17430 | 57 | 52012 | 0.022 |
| GO:0005938 | C | cell cortex | 31 | 17430 | 57 | 52012 | 0.022 |
| GO:0044421 | C | extracellular region part | 12 | 17430 | 15 | 52012 | 0.022 |
| GO:0070461 | C | SAGA-type complex | 10 | 17430 | 11 | 52012 | 0.021 |
| GO:0005694 | C | chromosome | 125 | 17430 | 300 | 52012 | 0.024 |
| GO:0042175 | C | nuclear outer membrane-endoplasmic reticulum membrane network | 70 | 17430 | 155 | 52012 | 0.024 |
| GO:0005789 | C | endoplasmic reticulum membrane | 70 | 17430 | 155 | 52012 | 0.024 |
| GO:0005801 | C | cis-Golgi network | 19 | 17430 | 30 | 52012 | 0.024 |
| GO:0044432 | C | endoplasmic reticulum part | 70 | 17430 | 156 | 52012 | 0.027 |
| GO:0044444 | C | cytoplasmic part | 984 | 17430 | 2741 | 52012 | 0.03 |
| GO:0043229 | C | intracellular organelle | 1989 | 17430 | 5670 | 52012 | 0.033 |
| GO:0043226 | C | organelle | 1989 | 17430 | 5670 | 52012 | 0.033 |
| GO:0005819 | C | spindle | 17 | 17430 | 27 | 52012 | 0.033 |
| GO:0009539 | C | photosystem II reaction center | 7 | 17430 | 7 | 52012 | 0.039 |
| GO:0098796 | C | membrane protein complex | 322 | 17430 | 854 | 52012 | 0.038 |
| GO:0005744 | C | mitochondrial inner membrane presequence translocase complex | 7 | 17430 | 7 | 52012 | 0.039 |
| GO:0012511 | C | monolayer-surrounded lipid storage body | 10 | 17430 | 13 | 52012 | 0.042 |
| GO:0030684 | C | preribosome | 10 | 17430 | 13 | 52012 | 0.042 |
| GO:0005811 | C | lipid particle | 10 | 17430 | 13 | 52012 | 0.042 |
| GO:0031012 | C | extracellular matrix | 9 | 17430 | 11 | 52012 | 0.042 |
| GO:0000785 | C | chromatin | 63 | 17430 | 143 | 52012 | 0.043 |
| GO:0005681 | C | spliceosomal complex | 16 | 17430 | 26 | 52012 | 0.043 |
| GO:0005839 | C | proteasome core complex | 35 | 17430 | 101 | 52012 | 0.46 |
| GO:0005834 | C | heterotrimeric G-protein complex | 5 | 17430 | 11 | 52012 | 0.37 |
| GO:0005956 | C | protein kinase CK2 complex | 8 | 17430 | 31 | 52012 | 0.8 |
| GO:0044815 | C | DNA packaging complex | 51 | 17430 | 126 | 52012 | 0.15 |
| GO:0008287 | C | protein serine/threonine phosphatase complex | 34 | 17430 | 271 | 52012 | 1 |
| GO:0019897 | C | extrinsic component of plasma membrane | 5 | 17430 | 11 | 52012 | 0.37 |
| GO:0005829 | C | cytosol | 8 | 17430 | 66 | 52012 | 1 |
| GO:0030665 | C | clathrin-coated vesicle membrane | 8 | 17430 | 22 | 52012 | 0.49 |
| GO:0030660 | C | Golgi-associated vesicle membrane | 21 | 17430 | 57 | 52012 | 0.4 |
| GO:0030663 | C | COPI-coated vesicle membrane | 13 | 17430 | 35 | 52012 | 0.43 |
| GO:0030662 | C | coated vesicle membrane | 39 | 17430 | 120 | 52012 | 0.6 |
| GO:0000178 | C | exosome (RNase complex) | 7 | 17430 | 10 | 52012 | 0.11 |
| GO:0044391 | C | ribosomal subunit | 29 | 17430 | 185 | 52012 | 1 |
| GO:0048046 | C | apoplast | 37 | 17430 | 193 | 52012 | 1 |
| GO:0045261 | C | proton-transporting ATP synthase complex, catalytic core F(1) | 19 | 17430 | 34 | 52012 | 0.054 |
| GO:0012506 | C | vesicle membrane | 39 | 17430 | 120 | 52012 | 0.6 |
| GO:0012507 | C | ER to Golgi transport vesicle membrane | 18 | 17430 | 63 | 52012 | 0.76 |
| GO:1990204 | C | oxidoreductase complex | 34 | 17430 | 102 | 52012 | 0.54 |
| GO:0042579 | C | microbody | 31 | 17430 | 78 | 52012 | 0.24 |
| GO:0005732 | C | small nucleolar ribonucleoprotein complex | 6 | 17430 | 7 | 52012 | 0.082 |
| GO:0005730 | C | nucleolus | 7 | 17430 | 19 | 52012 | 0.49 |
| GO:0030120 | C | vesicle coat | 39 | 17430 | 120 | 52012 | 0.6 |
| GO:0030126 | C | COPI vesicle coat | 13 | 17430 | 35 | 52012 | 0.43 |
| GO:0030127 | C | COPII vesicle coat | 18 | 17430 | 63 | 52012 | 0.76 |
| GO:0030125 | C | clathrin vesicle coat | 8 | 17430 | 22 | 52012 | 0.49 |
| GO:0000775 | C | chromosome, centromeric region | 13 | 17430 | 35 | 52012 | 0.43 |
| GO:0045263 | C | proton-transporting ATP synthase complex, coupling factor F(o) | 16 | 17430 | 48 | 52012 | 0.56 |
| GO:0033180 | C | proton-transporting V-type ATPase, V1 domain | 8 | 17430 | 47 | 52012 | 0.98 |
| GO:0000502 | C | proteasome complex | 45 | 17430 | 121 | 52012 | 0.3 |
| GO:0097708 | C | intracellular vesicle | 39 | 17430 | 123 | 52012 | 0.65 |
| GO:0005905 | C | clathrin-coated pit | 8 | 17430 | 29 | 52012 | 0.75 |
| GO:0012510 | C | trans-Golgi network transport vesicle membrane | 8 | 17430 | 22 | 52012 | 0.49 |
| GO:0061695 | C | transferase complex, transferring phosphorus-containing groups | 29 | 17430 | 149 | 52012 | 1 |
| GO:0030130 | C | clathrin coat of trans-Golgi network vesicle | 8 | 17430 | 22 | 52012 | 0.49 |
| GO:0030133 | C | transport vesicle | 26 | 17430 | 85 | 52012 | 0.69 |
| GO:0030132 | C | clathrin coat of coated pit | 8 | 17430 | 29 | 52012 | 0.75 |
| GO:0030135 | C | coated vesicle | 39 | 17430 | 120 | 52012 | 0.6 |
| GO:0030134 | C | ER to Golgi transport vesicle | 18 | 17430 | 63 | 52012 | 0.76 |
| GO:0030137 | C | COPI-coated vesicle | 13 | 17430 | 35 | 52012 | 0.43 |
| GO:0030136 | C | clathrin-coated vesicle | 8 | 17430 | 22 | 52012 | 0.49 |
| GO:0031975 | C | envelope | 89 | 17430 | 359 | 52012 | 1 |
| GO:0019866 | C | organelle inner membrane | 45 | 17430 | 120 | 52012 | 0.29 |
| GO:1903293 | C | phosphatase complex | 34 | 17430 | 271 | 52012 | 1 |
| GO:0042765 | C | GPI-anchor transamidase complex | 5 | 17430 | 6 | 52012 | 0.12 |
| GO:0000159 | C | protein phosphatase type 2A complex | 34 | 17430 | 80 | 52012 | 0.15 |
| GO:0000151 | C | ubiquitin ligase complex | 10 | 17430 | 232 | 52012 | 1 |
| GO:0000152 | C | nuclear ubiquitin ligase complex | 8 | 17430 | 16 | 52012 | 0.24 |
| GO:0019898 | C | extrinsic component of membrane | 28 | 17430 | 55 | 52012 | 0.049 |
| GO:0005787 | C | signal peptidase complex | 8 | 17430 | 23 | 52012 | 0.53 |
| GO:0005783 | C | endoplasmic reticulum | 123 | 17430 | 397 | 52012 | 0.79 |
| GO:0030117 | C | membrane coat | 64 | 17430 | 305 | 52012 | 1 |
| GO:0030119 | C | AP-type membrane coat adaptor complex | 16 | 17430 | 70 | 52012 | 0.94 |
| GO:0030118 | C | clathrin coat | 16 | 17430 | 139 | 52012 | 1 |
| GO:0005623 | C | cell | 2747 | 17430 | 10436 | 52012 | 1 |
| GO:0000786 | C | nucleosome | 46 | 17430 | 126 | 52012 | 0.34 |
| GO:0000148 | C | 1,3-beta-D-glucan synthase complex | 16 | 17430 | 40 | 52012 | 0.32 |
| GO:0000428 | C | DNA-directed RNA polymerase complex | 24 | 17430 | 86 | 52012 | 0.82 |
| GO:0030131 | C | clathrin adaptor complex | 8 | 17430 | 70 | 52012 | 1 |
| GO:0030904 | C | retromer complex | 10 | 17430 | 28 | 52012 | 0.49 |
| GO:0031982 | C | vesicle | 39 | 17430 | 123 | 52012 | 0.65 |
| GO:0005753 | C | mitochondrial proton-transporting ATP synthase complex | 17 | 17430 | 36 | 52012 | 0.16 |
| GO:0031988 | C | membrane-bounded vesicle | 39 | 17430 | 123 | 52012 | 0.65 |
| GO:0005798 | C | Golgi-associated vesicle | 21 | 17430 | 57 | 52012 | 0.4 |
| GO:0005759 | C | mitochondrial matrix | 8 | 17430 | 17 | 52012 | 0.28 |
| GO:0005667 | C | transcription factor complex | 27 | 17430 | 141 | 52012 | 1 |
| GO:0048500 | C | signal recognition particle | 8 | 17430 | 21 | 52012 | 0.45 |
| GO:0005669 | C | transcription factor TFIID complex | 6 | 17430 | 23 | 52012 | 0.77 |
| GO:0000228 | C | nuclear chromosome | 32 | 17430 | 67 | 52012 | 0.064 |
| GO:0098687 | C | chromosomal region | 15 | 17430 | 43 | 52012 | 0.5 |
| GO:0000139 | C | Golgi membrane | 15 | 17430 | 59 | 52012 | 0.86 |
| GO:0031410 | C | cytoplasmic vesicle | 39 | 17430 | 123 | 52012 | 0.65 |
| GO:0032040 | C | small-subunit processome | 7 | 17430 | 10 | 52012 | 0.11 |
| GO:0005576 | C | extracellular region | 64 | 17430 | 335 | 52012 | 1 |
| GO:0005886 | C | plasma membrane | 46 | 17430 | 122 | 52012 | 0.27 |
| GO:0032993 | C | protein-DNA complex | 46 | 17430 | 126 | 52012 | 0.34 |
| GO:0044798 | C | nuclear transcription factor complex | 17 | 17430 | 107 | 52012 | 1 |
| GO:0016459 | C | myosin complex | 32 | 17430 | 79 | 52012 | 0.21 |
| GO:0044429 | C | mitochondrial part | 83 | 17430 | 255 | 52012 | 0.61 |
| GO:0044424 | C | intracellular part | 2502 | 17430 | 7865 | 52012 | 0.99 |
| GO:0009898 | C | cytoplasmic side of plasma membrane | 5 | 17430 | 11 | 52012 | 0.37 |
| GO:1990351 | C | transporter complex | 7 | 17430 | 22 | 52012 | 0.62 |
| GO:0031227 | C | intrinsic component of endoplasmic reticulum membrane | 10 | 17430 | 44 | 52012 | 0.9 |
| GO:0016592 | C | mediator complex | 18 | 17430 | 54 | 52012 | 0.55 |
| GO:0016591 | C | DNA-directed RNA polymerase II, holoenzyme | 23 | 17430 | 78 | 52012 | 0.74 |
| GO:0016023 | C | cytoplasmic, membrane-bounded vesicle | 39 | 17430 | 123 | 52012 | 0.65 |
| GO:0016020 | C | membrane | 2283 | 17430 | 6593 | 52012 | 0.076 |
| GO:0043231 | C | intracellular membrane-bounded organelle | 1206 | 17430 | 4042 | 52012 | 1 |
| GO:0045259 | C | proton-transporting ATP synthase complex | 35 | 17430 | 82 | 52012 | 0.14 |
| GO:0044433 | C | cytoplasmic vesicle part | 39 | 17430 | 120 | 52012 | 0.6 |
| GO:0044431 | C | Golgi apparatus part | 43 | 17430 | 140 | 52012 | 0.72 |
| GO:0044437 | C | vacuolar part | 10 | 17430 | 19 | 52012 | 0.17 |
| GO:0044439 | C | peroxisomal part | 14 | 17430 | 40 | 52012 | 0.5 |
| GO:0044438 | C | microbody part | 14 | 17430 | 40 | 52012 | 0.5 |
| GO:0030140 | C | trans-Golgi network transport vesicle | 8 | 17430 | 22 | 52012 | 0.49 |
| GO:0009507 | C | chloroplast | 22 | 17430 | 94 | 52012 | 0.95 |
| GO:0005643 | C | nuclear pore | 13 | 17430 | 47 | 52012 | 0.77 |
| GO:0031301 | C | integral component of organelle membrane | 12 | 17430 | 45 | 52012 | 0.8 |
| GO:0031300 | C | intrinsic component of organelle membrane | 12 | 17430 | 45 | 52012 | 0.8 |
| GO:0031090 | C | organelle membrane | 154 | 17430 | 452 | 52012 | 0.44 |
| GO:0031231 | C | intrinsic component of peroxisomal membrane | 10 | 17430 | 23 | 52012 | 0.3 |
| GO:0042651 | C | thylakoid membrane | 28 | 17430 | 86 | 52012 | 0.59 |
| GO:0031234 | C | extrinsic component of cytoplasmic side of plasma membrane | 5 | 17430 | 11 | 52012 | 0.37 |
| GO:0098552 | C | side of membrane | 5 | 17430 | 11 | 52012 | 0.37 |
| GO:0009538 | C | photosystem I reaction center | 10 | 17430 | 16 | 52012 | 0.093 |
| GO:0000922 | C | spindle pole | 8 | 17430 | 27 | 52012 | 0.68 |
| GO:0016272 | C | prefoldin complex | 9 | 17430 | 26 | 52012 | 0.53 |
| GO:0043227 | C | membrane-bounded organelle | 1206 | 17430 | 4042 | 52012 | 1 |
| GO:0016471 | C | vacuolar proton-transporting V-type ATPase complex | 6 | 17430 | 19 | 52012 | 0.63 |
| GO:0033179 | C | proton-transporting V-type ATPase, V0 domain | 10 | 17430 | 37 | 52012 | 0.78 |
| GO:0044445 | C | cytosolic part | 7 | 17430 | 66 | 52012 | 1 |
| GO:0033177 | C | proton-transporting two-sector ATPase complex, proton-transporting domain | 43 | 17430 | 101 | 52012 | 0.11 |
| GO:0005634 | C | nucleus | 803 | 17430 | 2916 | 52012 | 1 |
| GO:0005742 | C | mitochondrial outer membrane translocase complex | 5 | 17430 | 24 | 52012 | 0.89 |
| GO:0005740 | C | mitochondrial envelope | 75 | 17430 | 238 | 52012 | 0.7 |
| GO:0005741 | C | mitochondrial outer membrane | 23 | 17430 | 77 | 52012 | 0.72 |
| GO:0005746 | C | mitochondrial respiratory chain | 9 | 17430 | 15 | 52012 | 0.12 |
| GO:0031968 | C | organelle outer membrane | 23 | 17430 | 83 | 52012 | 0.82 |
| GO:0009536 | C | plastid | 22 | 17430 | 116 | 52012 | 1 |
| GO:0098562 | C | cytoplasmic side of membrane | 5 | 17430 | 11 | 52012 | 0.37 |
| GO:0031966 | C | mitochondrial membrane | 68 | 17430 | 198 | 52012 | 0.45 |
| GO:0016469 | C | proton-transporting two-sector ATPase complex | 85 | 17430 | 226 | 52012 | 0.2 |
| GO:0015934 | C | large ribosomal subunit | 25 | 17430 | 90 | 52012 | 0.83 |
| GO:0044455 | C | mitochondrial membrane part | 38 | 17430 | 85 | 52012 | 0.086 |
| GO:0044459 | C | plasma membrane part | 34 | 17430 | 110 | 52012 | 0.69 |
| GO:0005622 | C | intracellular | 2608 | 17430 | 9868 | 52012 | 1 |
| GO:0009331 | C | glycerol-3-phosphate dehydrogenase complex | 5 | 17430 | 32 | 52012 | 0.97 |
| GO:0031967 | C | organelle envelope | 89 | 17430 | 297 | 52012 | 0.84 |
| GO:0098805 | C | whole membrane | 86 | 17430 | 269 | 52012 | 0.67 |
| GO:0098797 | C | plasma membrane protein complex | 31 | 17430 | 94 | 52012 | 0.57 |
| GO:0098799 | C | outer mitochondrial membrane protein complex | 5 | 17430 | 24 | 52012 | 0.89 |
| GO:0098798 | C | mitochondrial protein complex | 38 | 17430 | 80 | 52012 | 0.05 |
| GO:0048475 | C | coated membrane | 64 | 17430 | 305 | 52012 | 1 |
| GO:0019867 | C | outer membrane | 31 | 17430 | 112 | 52012 | 0.85 |
| GO:0030880 | C | RNA polymerase complex | 24 | 17430 | 89 | 52012 | 0.86 |
| GO:0044464 | C | cell part | 2747 | 17430 | 10436 | 52012 | 1 |
| GO:0000276 | C | mitochondrial proton-transporting ATP synthase complex, coupling factor F(o) | 13 | 17430 | 27 | 52012 | 0.18 |
| GO:0098588 | C | bounding membrane of organelle | 108 | 17430 | 325 | 52012 | 0.55 |
| GO:0098589 | C | membrane region | 8 | 17430 | 29 | 52012 | 0.75 |
| GO:0031461 | C | cullin-RING ubiquitin ligase complex | 8 | 17430 | 67 | 52012 | 1 |
| GO:0005794 | C | Golgi apparatus | 45 | 17430 | 142 | 52012 | 0.66 |
| GO:0000015 | C | phosphopyruvate hydratase complex | 5 | 17430 | 23 | 52012 | 0.87 |
| GO:0009654 | C | photosystem II oxygen evolving complex | 22 | 17430 | 46 | 52012 | 0.11 |
| GO:0019773 | C | proteasome core complex, alpha-subunit complex | 17 | 17430 | 43 | 52012 | 0.33 |
| GO:0000808 | C | origin recognition complex | 10 | 17430 | 36 | 52012 | 0.75 |
| GO:0005815 | C | microtubule organizing center | 13 | 17430 | 27 | 52012 | 0.18 |
| GO:0005680 | C | anaphase-promoting complex | 8 | 17430 | 16 | 52012 | 0.24 |
| GO:0005779 | C | integral component of peroxisomal membrane | 10 | 17430 | 23 | 52012 | 0.3 |
| GO:0005778 | C | peroxisomal membrane | 14 | 17430 | 40 | 52012 | 0.5 |
| GO:0005773 | C | vacuole | 10 | 17430 | 20 | 52012 | 0.2 |
| GO:0005777 | C | peroxisome | 31 | 17430 | 78 | 52012 | 0.24 |
| GO:0005774 | C | vacuolar membrane | 10 | 17430 | 19 | 52012 | 0.17 |
| GO:0031903 | C | microbody membrane | 14 | 17430 | 40 | 52012 | 0.5 |
| GO:0055029 | C | nuclear DNA-directed RNA polymerase complex | 24 | 17430 | 86 | 52012 | 0.82 |
| GO:0005743 | C | mitochondrial inner membrane | 44 | 17430 | 114 | 52012 | 0.24 |
| GO:0012505 | C | endomembrane system | 210 | 17430 | 690 | 52012 | 0.9 |
| GO:0090575 | C | RNA polymerase II transcription factor complex | 15 | 17430 | 107 | 52012 | 1 |
| GO:0000439 | C | core TFIIH complex | 6 | 17430 | 9 | 52012 | 0.15 |
| GO:0070469 | C | respiratory chain | 11 | 17430 | 18 | 52012 | 0.088 |
| GO:1902495 | C | transmembrane transporter complex | 7 | 17430 | 11 | 52012 | 0.14 |
| GO:1902494 | C | catalytic complex | 204 | 17430 | 935 | 52012 | 1 |
| GO:0033178 | C | proton-transporting two-sector ATPase complex, catalytic domain | 39 | 17430 | 109 | 52012 | 0.39 |
| GO:0033176 | C | proton-transporting V-type ATPase complex | 21 | 17430 | 95 | 52012 | 0.97 |
| GO:1990234 | C | transferase complex | 99 | 17430 | 486 | 52012 | 1 |
| GO:0005737 | C | cytoplasm | 1145 | 17430 | 3805 | 52012 | 1 |
| GO:0005635 | C | nuclear envelope | 14 | 17430 | 53 | 52012 | 0.82 |
| GO:0030658 | C | transport vesicle membrane | 26 | 17430 | 85 | 52012 | 0.69 |
| GO:0030659 | C | cytoplasmic vesicle membrane | 39 | 17430 | 120 | 52012 | 0.6 |
