# Supplementary material for: Comparative transcriptome analysis reveals evolutionary divergence and shared network of cold and salt stress response in diploid D-genome cotton
Source: BMC Plant Biol. 2020 Nov 12;20:518. doi: 10.1186/s12870-020-02726-4 (PMC7664088; doi:10.1186/s12870-020-02726-4)
Supplement: Supplementary file 5 — Additional files 5: Table S5. GO enrichment analysis of PSGs in wild species. [file 12870_2020_2726_MOESM5_ESM.docx]

Table S5 GO enrichment analysis of PSGs in wild species.

| **GO_acc** | **term_type** | **Term** | **queryitem** | **querytotal** | **bgitem** | **bgtotal** | **pvalue** |
| --- | --- | --- | --- | --- | --- | --- | --- |
| GO:0032446 | P | protein modification by small protein conjugation | 7 | 450 | 203 | 52012 | 0.0024 |
| GO:0016567 | P | protein ubiquitination | 7 | 450 | 198 | 52012 | 0.0021 |
| GO:0070647 | P | protein modification by small protein conjugation or removal | 7 | 450 | 203 | 52012 | 0.0024 |
| GO:0006396 | P | RNA processing | 15 | 450 | 655 | 52012 | 0.00079 |
| GO:0034470 | P | ncRNA processing | 7 | 450 | 225 | 52012 | 0.0041 |
| GO:0008104 | P | protein localization | 5 | 450 | 1369 | 52012 | 0.99 |
| GO:0080090 | P | regulation of primary metabolic process | 28 | 450 | 3507 | 52012 | 0.7 |
| GO:0019222 | P | regulation of metabolic process | 28 | 450 | 3531 | 52012 | 0.71 |
| GO:0044281 | P | small molecule metabolic process | 16 | 450 | 2983 | 52012 | 0.99 |
| GO:1901362 | P | organic cyclic compound biosynthetic process | 40 | 450 | 4573 | 52012 | 0.5 |
| GO:1901360 | P | organic cyclic compound metabolic process | 73 | 450 | 6972 | 52012 | 0.05 |
| GO:0051716 | P | cellular response to stimulus | 9 | 450 | 1838 | 52012 | 0.98 |
| GO:0044711 | P | single-organism biosynthetic process | 13 | 450 | 2083 | 52012 | 0.91 |
| GO:0070727 | P | cellular macromolecule localization | 5 | 450 | 1046 | 52012 | 0.95 |
| GO:0043043 | P | peptide biosynthetic process | 10 | 450 | 1750 | 52012 | 0.94 |
| GO:0016310 | P | phosphorylation | 33 | 450 | 4109 | 52012 | 0.7 |
| GO:0033036 | P | macromolecule localization | 7 | 450 | 1630 | 52012 | 0.99 |
| GO:0009058 | P | biosynthetic process | 62 | 450 | 8504 | 52012 | 0.94 |
| GO:0006281 | P | DNA repair | 5 | 450 | 484 | 52012 | 0.41 |
| GO:0060255 | P | regulation of macromolecule metabolic process | 28 | 450 | 3517 | 52012 | 0.7 |
| GO:0045184 | P | establishment of protein localization | 5 | 450 | 1323 | 52012 | 0.99 |
| GO:2001141 | P | regulation of RNA biosynthetic process | 27 | 450 | 3321 | 52012 | 0.66 |
| GO:0043436 | P | oxoacid metabolic process | 6 | 450 | 1570 | 52012 | 0.99 |
| GO:0055114 | P | oxidation-reduction process | 27 | 450 | 3824 | 52012 | 0.88 |
| GO:0046483 | P | heterocycle metabolic process | 70 | 450 | 6782 | 52012 | 0.068 |
| GO:1901564 | P | organonitrogen compound metabolic process | 23 | 450 | 3938 | 52012 | 0.98 |
| GO:0016192 | P | vesicle-mediated transport | 5 | 450 | 682 | 52012 | 0.7 |
| GO:1901566 | P | organonitrogen compound biosynthetic process | 18 | 450 | 2975 | 52012 | 0.96 |
| GO:0044249 | P | cellular biosynthetic process | 53 | 450 | 8039 | 52012 | 0.99 |
| GO:0019538 | P | protein metabolic process | 65 | 450 | 8223 | 52012 | 0.8 |
| GO:0033554 | P | cellular response to stress | 5 | 450 | 490 | 52012 | 0.42 |
| GO:0019637 | P | organophosphate metabolic process | 7 | 450 | 1292 | 52012 | 0.93 |
| GO:0019438 | P | aromatic compound biosynthetic process | 35 | 450 | 4449 | 52012 | 0.74 |
| GO:0022607 | P | cellular component assembly | 6 | 450 | 463 | 52012 | 0.22 |
| GO:0006807 | P | nitrogen compound metabolic process | 81 | 450 | 9325 | 52012 | 0.5 |
| GO:0034660 | P | ncRNA metabolic process | 10 | 450 | 480 | 52012 | 0.01 |
| GO:0050789 | P | regulation of biological process | 34 | 450 | 5130 | 52012 | 0.96 |
| GO:0097659 | P | nucleic acid-templated transcription | 31 | 450 | 3727 | 52012 | 0.62 |
| GO:0044267 | P | cellular protein metabolic process | 59 | 450 | 6950 | 52012 | 0.58 |
| GO:1901575 | P | organic substance catabolic process | 5 | 450 | 1139 | 52012 | 0.97 |
| GO:0044262 | P | cellular carbohydrate metabolic process | 5 | 450 | 537 | 52012 | 0.5 |
| GO:0044260 | P | cellular macromolecule metabolic process | 116 | 450 | 12324 | 52012 | 0.16 |
| GO:0006886 | P | intracellular protein transport | 5 | 450 | 991 | 52012 | 0.93 |
| GO:0016043 | P | cellular component organization | 17 | 450 | 1162 | 52012 | 0.027 |
| GO:0065003 | P | macromolecular complex assembly | 5 | 450 | 429 | 52012 | 0.32 |
| GO:0065007 | P | biological regulation | 36 | 450 | 5353 | 52012 | 0.96 |
| GO:0071840 | P | cellular component organization or biogenesis | 19 | 450 | 1282 | 52012 | 0.018 |
| GO:0018130 | P | heterocycle biosynthetic process | 37 | 450 | 4465 | 52012 | 0.63 |
| GO:0034613 | P | cellular protein localization | 5 | 450 | 1046 | 52012 | 0.95 |
| GO:0006629 | P | lipid metabolic process | 10 | 450 | 1561 | 52012 | 0.87 |
| GO:0006810 | P | transport | 36 | 450 | 4999 | 52012 | 0.89 |
| GO:0009889 | P | regulation of biosynthetic process | 27 | 450 | 3376 | 52012 | 0.69 |
| GO:0044710 | P | single-organism metabolic process | 51 | 450 | 7815 | 52012 | 0.99 |
| GO:0050794 | P | regulation of cellular process | 34 | 450 | 5061 | 52012 | 0.95 |
| GO:0043412 | P | macromolecule modification | 51 | 450 | 4833 | 52012 | 0.083 |
| GO:0036211 | P | protein modification process | 47 | 450 | 4679 | 52012 | 0.16 |
| GO:0008152 | P | metabolic process | 202 | 450 | 24203 | 52012 | 0.77 |
| GO:0044723 | P | single-organism carbohydrate metabolic process | 7 | 450 | 1036 | 52012 | 0.79 |
| GO:0051234 | P | establishment of localization | 36 | 450 | 5020 | 52012 | 0.9 |
| GO:0016070 | P | RNA metabolic process | 51 | 450 | 4744 | 52012 | 0.065 |
| GO:0044271 | P | cellular nitrogen compound biosynthetic process | 47 | 450 | 6174 | 52012 | 0.84 |
| GO:0046907 | P | intracellular transport | 8 | 450 | 1069 | 52012 | 0.71 |
| GO:0050896 | P | response to stimulus | 18 | 450 | 3207 | 52012 | 0.98 |
| GO:0006950 | P | response to stress | 9 | 450 | 1440 | 52012 | 0.88 |
| GO:0006355 | P | regulation of transcription, DNA-templated | 27 | 450 | 3321 | 52012 | 0.66 |
| GO:0010556 | P | regulation of macromolecule biosynthetic process | 27 | 450 | 3366 | 52012 | 0.68 |
| GO:0006351 | P | transcription, DNA-templated | 31 | 450 | 3727 | 52012 | 0.62 |
| GO:0006518 | P | peptide metabolic process | 10 | 450 | 1802 | 52012 | 0.95 |
| GO:0032774 | P | RNA biosynthetic process | 31 | 450 | 3730 | 52012 | 0.62 |
| GO:0070271 | P | protein complex biogenesis | 5 | 450 | 415 | 52012 | 0.29 |
| GO:0034654 | P | nucleobase-containing compound biosynthetic process | 33 | 450 | 4164 | 52012 | 0.72 |
| GO:0034641 | P | cellular nitrogen compound metabolic process | 77 | 450 | 8317 | 52012 | 0.28 |
| GO:0034645 | P | cellular macromolecule biosynthetic process | 42 | 450 | 6113 | 52012 | 0.96 |
| GO:0007154 | P | cell communication | 6 | 450 | 1521 | 52012 | 0.99 |
| GO:0044699 | P | single-organism process | 80 | 450 | 11479 | 52012 | 0.99 |
| GO:0006139 | P | nucleobase-containing compound metabolic process | 66 | 450 | 6446 | 52012 | 0.085 |
| GO:0006508 | P | proteolysis | 7 | 450 | 1664 | 52012 | 0.99 |
| GO:0008610 | P | lipid biosynthetic process | 5 | 450 | 628 | 52012 | 0.63 |
| GO:0008033 | P | tRNA processing | 5 | 450 | 170 | 52012 | 0.018 |
| GO:0009987 | P | cellular process | 161 | 450 | 20063 | 52012 | 0.9 |
| GO:0006725 | P | cellular aromatic compound metabolic process | 69 | 450 | 6894 | 52012 | 0.11 |
| GO:1903506 | P | regulation of nucleic acid-templated transcription | 27 | 450 | 3321 | 52012 | 0.66 |
| GO:0006974 | P | cellular response to DNA damage stimulus | 5 | 450 | 485 | 52012 | 0.41 |
| GO:0007010 | P | cytoskeleton organization | 6 | 450 | 198 | 52012 | 0.0087 |
| GO:0055085 | P | transmembrane transport | 16 | 450 | 2073 | 52012 | 0.71 |
| GO:0055086 | P | nucleobase-containing small molecule metabolic process | 6 | 450 | 897 | 52012 | 0.79 |
| GO:0043604 | P | amide biosynthetic process | 10 | 450 | 1753 | 52012 | 0.94 |
| GO:0043603 | P | cellular amide metabolic process | 10 | 450 | 1839 | 52012 | 0.96 |
| GO:1901135 | P | carbohydrate derivative metabolic process | 5 | 450 | 1166 | 52012 | 0.97 |
| GO:0051252 | P | regulation of RNA metabolic process | 28 | 450 | 3329 | 52012 | 0.59 |
| GO:0043170 | P | macromolecule metabolic process | 122 | 450 | 13664 | 52012 | 0.36 |
| GO:0043933 | P | macromolecular complex subunit organization | 6 | 450 | 587 | 52012 | 0.4 |
| GO:0031326 | P | regulation of cellular biosynthetic process | 27 | 450 | 3376 | 52012 | 0.69 |
| GO:0031323 | P | regulation of cellular metabolic process | 28 | 450 | 3502 | 52012 | 0.69 |
| GO:0019752 | P | carboxylic acid metabolic process | 6 | 450 | 1567 | 52012 | 0.99 |
| GO:0090304 | P | nucleic acid metabolic process | 60 | 450 | 5491 | 52012 | 0.037 |
| GO:0071822 | P | protein complex subunit organization | 5 | 450 | 469 | 52012 | 0.38 |
| GO:2000112 | P | regulation of cellular macromolecule biosynthetic process | 27 | 450 | 3366 | 52012 | 0.68 |
| GO:1902589 | P | single-organism organelle organization | 6 | 450 | 216 | 52012 | 0.013 |
| GO:0071704 | P | organic substance metabolic process | 159 | 450 | 19075 | 52012 | 0.74 |
| GO:0010467 | P | gene expression | 56 | 450 | 6121 | 52012 | 0.35 |
| GO:0006082 | P | organic acid metabolic process | 6 | 450 | 1768 | 52012 | 1 |
| GO:0071702 | P | organic substance transport | 8 | 450 | 1805 | 52012 | 0.99 |
| GO:0010468 | P | regulation of gene expression | 28 | 450 | 3371 | 52012 | 0.62 |
| GO:0006468 | P | protein phosphorylation | 32 | 450 | 3669 | 52012 | 0.51 |
| GO:1901576 | P | organic substance biosynthetic process | 57 | 450 | 8122 | 52012 | 0.97 |
| GO:0019219 | P | regulation of nucleobase-containing compound metabolic process | 28 | 450 | 3339 | 52012 | 0.6 |
| GO:0006461 | P | protein complex assembly | 5 | 450 | 415 | 52012 | 0.29 |
| GO:0006464 | P | cellular protein modification process | 47 | 450 | 4679 | 52012 | 0.16 |
| GO:0015031 | P | protein transport | 5 | 450 | 1302 | 52012 | 0.99 |
| GO:0044765 | P | single-organism transport | 11 | 450 | 1208 | 52012 | 0.47 |
| GO:0009059 | P | macromolecule biosynthetic process | 42 | 450 | 6117 | 52012 | 0.96 |
| GO:0044763 | P | single-organism cellular process | 42 | 450 | 7018 | 52012 | 1 |
| GO:0051171 | P | regulation of nitrogen compound metabolic process | 28 | 450 | 3389 | 52012 | 0.63 |
| GO:0051649 | P | establishment of localization in cell | 8 | 450 | 1069 | 52012 | 0.71 |
| GO:0042221 | P | response to chemical | 5 | 450 | 395 | 52012 | 0.26 |
| GO:0009056 | P | catabolic process | 6 | 450 | 1185 | 52012 | 0.94 |
| GO:0051179 | P | localization | 36 | 450 | 5066 | 52012 | 0.91 |
| GO:1902578 | P | single-organism localization | 11 | 450 | 1263 | 52012 | 0.53 |
| GO:0051641 | P | cellular localization | 8 | 450 | 1124 | 52012 | 0.76 |
| GO:0006996 | P | organelle organization | 11 | 450 | 671 | 52012 | 0.035 |
| GO:0044238 | P | primary metabolic process | 153 | 450 | 18025 | 52012 | 0.63 |
| GO:0005975 | P | carbohydrate metabolic process | 16 | 450 | 2274 | 52012 | 0.83 |
| GO:0044237 | P | cellular metabolic process | 138 | 450 | 16458 | 52012 | 0.69 |
| GO:0006399 | P | tRNA metabolic process | 8 | 450 | 403 | 52012 | 0.027 |
| GO:0006796 | P | phosphate-containing compound metabolic process | 41 | 450 | 5379 | 52012 | 0.82 |
| GO:0044085 | P | cellular component biogenesis | 8 | 450 | 589 | 52012 | 0.14 |
| GO:0006793 | P | phosphorus metabolic process | 41 | 450 | 5392 | 52012 | 0.83 |
| GO:0006259 | P | DNA metabolic process | 9 | 450 | 795 | 52012 | 0.26 |
| GO:0006412 | P | translation | 10 | 450 | 1731 | 52012 | 0.93 |
| GO:0004540 | F | ribonuclease activity | 6 | 450 | 158 | 52012 | 0.003 |
| GO:0016765 | F | transferase activity, transferring alkyl or aryl (other than methyl) groups | 6 | 450 | 156 | 52012 | 0.0029 |
| GO:0004842 | F | ubiquitin-protein transferase activity | 7 | 450 | 229 | 52012 | 0.0045 |
| GO:0019787 | F | ubiquitin-like protein transferase activity | 7 | 450 | 229 | 52012 | 0.0045 |
| GO:0004521 | F | endoribonuclease activity | 5 | 450 | 114 | 52012 | 0.0037 |
| GO:0008168 | F | methyltransferase activity | 14 | 450 | 743 | 52012 | 0.0064 |
| GO:0016741 | F | transferase activity, transferring one-carbon groups | 14 | 450 | 818 | 52012 | 0.014 |
| GO:0043531 | F | ADP binding | 8 | 450 | 379 | 52012 | 0.019 |
| GO:0004519 | F | endonuclease activity | 6 | 450 | 236 | 52012 | 0.019 |
| GO:0043169 | F | cation binding | 52 | 450 | 8202 | 52012 | 1 |
| GO:1901363 | F | heterocyclic compound binding | 127 | 450 | 18860 | 52012 | 1 |
| GO:0046983 | F | protein dimerization activity | 9 | 450 | 1009 | 52012 | 0.51 |
| GO:1901265 | F | nucleoside phosphate binding | 69 | 450 | 11390 | 52012 | 1 |
| GO:0000166 | F | nucleotide binding | 69 | 450 | 11390 | 52012 | 1 |
| GO:0016740 | F | transferase activity | 88 | 450 | 8540 | 52012 | 0.045 |
| GO:0016746 | F | transferase activity, transferring acyl groups | 6 | 450 | 777 | 52012 | 0.66 |
| GO:0097367 | F | carbohydrate derivative binding | 64 | 450 | 8366 | 52012 | 0.87 |
| GO:0004672 | F | protein kinase activity | 32 | 450 | 3556 | 52012 | 0.44 |
| GO:0016614 | F | oxidoreductase activity, acting on CH-OH group of donors | 6 | 450 | 530 | 52012 | 0.31 |
| GO:0016818 | F | hydrolase activity, acting on acid anhydrides, in phosphorus-containing anhydrides | 9 | 450 | 2759 | 52012 | 1 |
| GO:0016817 | F | hydrolase activity, acting on acid anhydrides | 9 | 450 | 2832 | 52012 | 1 |
| GO:0005524 | F | ATP binding | 51 | 450 | 6846 | 52012 | 0.89 |
| GO:0004553 | F | hydrolase activity, hydrolyzing O-glycosyl compounds | 5 | 450 | 963 | 52012 | 0.92 |
| GO:0005525 | F | GTP binding | 5 | 450 | 948 | 52012 | 0.91 |
| GO:0001071 | F | nucleic acid binding transcription factor activity | 12 | 450 | 1741 | 52012 | 0.82 |
| GO:0005488 | F | binding | 241 | 450 | 34020 | 52012 | 1 |
| GO:0003676 | F | nucleic acid binding | 66 | 450 | 8571 | 52012 | 0.86 |
| GO:0003677 | F | DNA binding | 31 | 450 | 4922 | 52012 | 0.98 |
| GO:0032549 | F | ribonucleoside binding | 64 | 450 | 8189 | 52012 | 0.83 |
| GO:0017076 | F | purine nucleotide binding | 65 | 450 | 8193 | 52012 | 0.79 |
| GO:0022804 | F | active transmembrane transporter activity | 6 | 450 | 976 | 52012 | 0.85 |
| GO:0016787 | F | hydrolase activity | 47 | 450 | 8107 | 52012 | 1 |
| GO:0020037 | F | heme binding | 5 | 450 | 842 | 52012 | 0.85 |
| GO:0043565 | F | sequence-specific DNA binding | 8 | 450 | 1132 | 52012 | 0.76 |
| GO:0016788 | F | hydrolase activity, acting on ester bonds | 17 | 450 | 2096 | 52012 | 0.64 |
| GO:0003824 | F | catalytic activity | 188 | 450 | 24009 | 52012 | 0.97 |
| GO:0016758 | F | transferase activity, transferring hexosyl groups | 11 | 450 | 878 | 52012 | 0.15 |
| GO:0022892 | F | substrate-specific transporter activity | 7 | 450 | 1691 | 52012 | 0.99 |
| GO:0046906 | F | tetrapyrrole binding | 5 | 450 | 864 | 52012 | 0.87 |
| GO:0016773 | F | phosphotransferase activity, alcohol group as acceptor | 34 | 450 | 4173 | 52012 | 0.67 |
| GO:0016772 | F | transferase activity, transferring phosphorus-containing groups | 40 | 450 | 5055 | 52012 | 0.75 |
| GO:0016301 | F | kinase activity | 33 | 450 | 4158 | 52012 | 0.72 |
| GO:0032559 | F | adenyl ribonucleotide binding | 59 | 450 | 7232 | 52012 | 0.71 |
| GO:0032555 | F | purine ribonucleotide binding | 64 | 450 | 8163 | 52012 | 0.82 |
| GO:0008194 | F | UDP-glycosyltransferase activity | 6 | 450 | 345 | 52012 | 0.083 |
| GO:0016757 | F | transferase activity, transferring glycosyl groups | 15 | 450 | 1169 | 52012 | 0.088 |
| GO:0032553 | F | ribonucleotide binding | 64 | 450 | 8311 | 52012 | 0.86 |
| GO:0008270 | F | zinc ion binding | 31 | 450 | 4422 | 52012 | 0.91 |
| GO:0016705 | F | oxidoreductase activity, acting on paired donors, with incorporation or reduction of molecular oxygen | 7 | 450 | 1093 | 52012 | 0.83 |
| GO:0035639 | F | purine ribonucleoside triphosphate binding | 56 | 450 | 7777 | 52012 | 0.94 |
| GO:0016798 | F | hydrolase activity, acting on glycosyl bonds | 5 | 450 | 1048 | 52012 | 0.95 |
| GO:0008233 | F | peptidase activity | 5 | 450 | 1440 | 52012 | 0.99 |
| GO:0016491 | F | oxidoreductase activity | 31 | 450 | 4329 | 52012 | 0.88 |
| GO:0016874 | F | ligase activity | 6 | 450 | 989 | 52012 | 0.86 |
| GO:0000287 | F | magnesium ion binding | 5 | 450 | 426 | 52012 | 0.31 |
| GO:0043167 | F | ion binding | 53 | 450 | 8723 | 52012 | 1 |
| GO:0032561 | F | guanyl ribonucleotide binding | 5 | 450 | 948 | 52012 | 0.91 |
| GO:0005506 | F | iron ion binding | 6 | 450 | 983 | 52012 | 0.85 |
| GO:0015297 | F | antiporter activity | 5 | 450 | 271 | 52012 | 0.09 |
| GO:0005215 | F | transporter activity | 19 | 450 | 2603 | 52012 | 0.81 |
| GO:0048037 | F | cofactor binding | 9 | 450 | 1648 | 52012 | 0.95 |
| GO:0046872 | F | metal ion binding | 52 | 450 | 7694 | 52012 | 0.98 |
| GO:0030554 | F | adenyl nucleotide binding | 60 | 450 | 7245 | 52012 | 0.66 |
| GO:0004518 | F | nuclease activity | 8 | 450 | 478 | 52012 | 0.06 |
| GO:0016616 | F | oxidoreductase activity, acting on the CH-OH group of donors, NAD or NADP as acceptor | 6 | 450 | 477 | 52012 | 0.24 |
| GO:0003723 | F | RNA binding | 14 | 450 | 1378 | 52012 | 0.31 |
| GO:0005515 | F | protein binding | 94 | 450 | 12774 | 52012 | 0.97 |
| GO:0097159 | F | organic cyclic compound binding | 127 | 450 | 18864 | 52012 | 1 |
| GO:0030246 | F | carbohydrate binding | 6 | 450 | 314 | 52012 | 0.059 |
| GO:0015291 | F | secondary active transmembrane transporter activity | 5 | 450 | 426 | 52012 | 0.31 |
| GO:0001883 | F | purine nucleoside binding | 64 | 450 | 8163 | 52012 | 0.82 |
| GO:0001882 | F | nucleoside binding | 64 | 450 | 8191 | 52012 | 0.83 |
| GO:0050662 | F | coenzyme binding | 8 | 450 | 1021 | 52012 | 0.66 |
| GO:0019001 | F | guanyl nucleotide binding | 5 | 450 | 965 | 52012 | 0.92 |
| GO:0005198 | F | structural molecule activity | 6 | 450 | 1500 | 52012 | 0.99 |
| GO:0003735 | F | structural constituent of ribosome | 6 | 450 | 1218 | 52012 | 0.95 |
| GO:0070011 | F | peptidase activity, acting on L-amino acid peptides | 5 | 450 | 1379 | 52012 | 0.99 |
| GO:0022857 | F | transmembrane transporter activity | 12 | 450 | 1956 | 52012 | 0.92 |
| GO:0017111 | F | nucleoside-triphosphatase activity | 7 | 450 | 2615 | 52012 | 1 |
| GO:0036094 | F | small molecule binding | 70 | 450 | 11530 | 52012 | 1 |
| GO:0046914 | F | transition metal ion binding | 38 | 450 | 5835 | 52012 | 0.98 |
| GO:0003700 | F | transcription factor activity, sequence-specific DNA binding | 12 | 450 | 1741 | 52012 | 0.82 |
| GO:0032550 | F | purine ribonucleoside binding | 64 | 450 | 8163 | 52012 | 0.82 |
| GO:0016462 | F | pyrophosphatase activity | 7 | 450 | 2710 | 52012 | 1 |
| GO:0031224 | C | intrinsic component of membrane | 26 | 450 | 2581 | 52012 | 0.24 |
| GO:0043228 | C | non-membrane-bounded organelle | 18 | 450 | 1875 | 52012 | 0.36 |
| GO:0030529 | C | intracellular ribonucleoprotein complex | 8 | 450 | 1328 | 52012 | 0.89 |
| GO:0016021 | C | integral component of membrane | 26 | 450 | 2545 | 52012 | 0.22 |
| GO:0016020 | C | membrane | 51 | 450 | 6593 | 52012 | 0.82 |
| GO:0043234 | C | protein complex | 22 | 450 | 2520 | 52012 | 0.51 |
| GO:0043231 | C | intracellular membrane-bounded organelle | 38 | 450 | 4042 | 52012 | 0.32 |
| GO:0044428 | C | nuclear part | 8 | 450 | 411 | 52012 | 0.029 |
| GO:0044422 | C | organelle part | 14 | 450 | 1680 | 52012 | 0.59 |
| GO:1990904 | C | ribonucleoprotein complex | 8 | 450 | 1328 | 52012 | 0.89 |
| GO:0044424 | C | intracellular part | 68 | 450 | 7865 | 52012 | 0.52 |
| GO:0043232 | C | intracellular non-membrane-bounded organelle | 18 | 450 | 1875 | 52012 | 0.36 |
| GO:0043229 | C | intracellular organelle | 54 | 450 | 5670 | 52012 | 0.25 |
| GO:0005622 | C | intracellular | 71 | 450 | 9868 | 52012 | 0.97 |
| GO:0043227 | C | membrane-bounded organelle | 38 | 450 | 4042 | 52012 | 0.32 |
| GO:0005856 | C | cytoskeleton | 6 | 450 | 322 | 52012 | 0.065 |
| GO:0044430 | C | cytoskeletal part | 5 | 450 | 213 | 52012 | 0.041 |
| GO:0012505 | C | endomembrane system | 5 | 450 | 690 | 52012 | 0.71 |
| GO:0005840 | C | ribosome | 6 | 450 | 1220 | 52012 | 0.95 |
| GO:0044446 | C | intracellular organelle part | 14 | 450 | 1677 | 52012 | 0.59 |
| GO:0044444 | C | cytoplasmic part | 16 | 450 | 2741 | 52012 | 0.96 |
| GO:0043226 | C | organelle | 54 | 450 | 5670 | 52012 | 0.25 |
| GO:0005634 | C | nucleus | 33 | 450 | 2916 | 52012 | 0.073 |
| GO:0005737 | C | cytoplasm | 19 | 450 | 3805 | 52012 | 1 |
| GO:0032991 | C | macromolecular complex | 30 | 450 | 3839 | 52012 | 0.74 |
| GO:0044464 | C | cell part | 74 | 450 | 10436 | 52012 | 0.98 |
| GO:0005623 | C | cell | 74 | 450 | 10436 | 52012 | 0.98 |
| GO:0044425 | C | membrane part | 29 | 450 | 3420 | 52012 | 0.57 |
