# Supplementary material for: Comparative transcriptome analysis reveals evolutionary divergence and shared network of cold and salt stress response in diploid D-genome cotton
Source: BMC Plant Biol. 2020 Nov 12;20:518. doi: 10.1186/s12870-020-02726-4 (PMC7664088; doi:10.1186/s12870-020-02726-4)
Supplement: Supplementary file 6 — Additional files 6: Table S6. GO enrichment analysis of PSGs in cultivated species. [file 12870_2020_2726_MOESM6_ESM.docx]

Table S6 GO enrichment analysis of PSGs in cultivted species.

| GO_acc | term_type | Term | queryitem | querytotal | bgitem | bgtotal | pvalue |
| --- | --- | --- | --- | --- | --- | --- | --- |
| GO:0016071 | P | mRNA metabolic process | 6 | 233 | 179 | 52012 | 0.00019 |
| GO:0080090 | P | regulation of primary metabolic process | 14 | 233 | 3507 | 52012 | 0.71 |
| GO:0019222 | P | regulation of metabolic process | 14 | 233 | 3531 | 52012 | 0.72 |
| GO:0044281 | P | small molecule metabolic process | 8 | 233 | 2983 | 52012 | 0.96 |
| GO:1901362 | P | organic cyclic compound biosynthetic process | 17 | 233 | 4573 | 52012 | 0.82 |
| GO:0071840 | P | cellular component organization or biogenesis | 9 | 233 | 1282 | 52012 | 0.13 |
| GO:0051716 | P | cellular response to stimulus | 5 | 233 | 1838 | 52012 | 0.92 |
| GO:0044711 | P | single-organism biosynthetic process | 6 | 233 | 2083 | 52012 | 0.91 |
| GO:0033036 | P | macromolecule localization | 5 | 233 | 1630 | 52012 | 0.86 |
| GO:0019725 | P | cellular homeostasis | 5 | 233 | 351 | 52012 | 0.022 |
| GO:0060255 | P | regulation of macromolecule metabolic process | 14 | 233 | 3517 | 52012 | 0.71 |
| GO:2001141 | P | regulation of RNA biosynthetic process | 14 | 233 | 3321 | 52012 | 0.63 |
| GO:0055114 | P | oxidation-reduction process | 13 | 233 | 3824 | 52012 | 0.88 |
| GO:0046483 | P | heterocycle metabolic process | 31 | 233 | 6782 | 52012 | 0.48 |
| GO:1901564 | P | organonitrogen compound metabolic process | 12 | 233 | 3938 | 52012 | 0.94 |
| GO:1901566 | P | organonitrogen compound biosynthetic process | 9 | 233 | 2975 | 52012 | 0.92 |
| GO:0044249 | P | cellular biosynthetic process | 28 | 233 | 8039 | 52012 | 0.94 |
| GO:0019538 | P | protein metabolic process | 28 | 233 | 8223 | 52012 | 0.96 |
| GO:0019438 | P | aromatic compound biosynthetic process | 17 | 233 | 4449 | 52012 | 0.79 |
| GO:0006807 | P | nitrogen compound metabolic process | 39 | 233 | 9325 | 52012 | 0.71 |
| GO:0050789 | P | regulation of biological process | 20 | 233 | 5130 | 52012 | 0.77 |
| GO:0097659 | P | nucleic acid-templated transcription | 14 | 233 | 3727 | 52012 | 0.79 |
| GO:1901576 | P | organic substance biosynthetic process | 28 | 233 | 8122 | 52012 | 0.95 |
| GO:1901575 | P | organic substance catabolic process | 5 | 233 | 1139 | 52012 | 0.58 |
| GO:0044260 | P | cellular macromolecule metabolic process | 52 | 233 | 12324 | 52012 | 0.71 |
| GO:0016043 | P | cellular component organization | 7 | 233 | 1162 | 52012 | 0.27 |
| GO:0065007 | P | biological regulation | 21 | 233 | 5353 | 52012 | 0.77 |
| GO:1901360 | P | organic cyclic compound metabolic process | 31 | 233 | 6972 | 52012 | 0.55 |
| GO:0065008 | P | regulation of biological quality | 5 | 233 | 521 | 52012 | 0.088 |
| GO:0018130 | P | heterocycle biosynthetic process | 17 | 233 | 4465 | 52012 | 0.79 |
| GO:0006810 | P | transport | 18 | 233 | 4999 | 52012 | 0.86 |
| GO:0009889 | P | regulation of biosynthetic process | 14 | 233 | 3376 | 52012 | 0.66 |
| GO:0044710 | P | single-organism metabolic process | 30 | 233 | 7815 | 52012 | 0.84 |
| GO:0050794 | P | regulation of cellular process | 20 | 233 | 5061 | 52012 | 0.75 |
| GO:0043412 | P | macromolecule modification | 21 | 233 | 4833 | 52012 | 0.59 |
| GO:0036211 | P | protein modification process | 19 | 233 | 4679 | 52012 | 0.71 |
| GO:0008152 | P | metabolic process | 97 | 233 | 24203 | 52012 | 0.94 |
| GO:0034654 | P | nucleobase-containing compound biosynthetic process | 15 | 233 | 4164 | 52012 | 0.84 |
| GO:0051234 | P | establishment of localization | 18 | 233 | 5020 | 52012 | 0.87 |
| GO:0016070 | P | RNA metabolic process | 24 | 233 | 4744 | 52012 | 0.3 |
| GO:0044271 | P | cellular nitrogen compound biosynthetic process | 22 | 233 | 6174 | 52012 | 0.9 |
| GO:0050896 | P | response to stimulus | 7 | 233 | 3207 | 52012 | 0.99 |
| GO:0006355 | P | regulation of transcription, DNA-templated | 14 | 233 | 3321 | 52012 | 0.63 |
| GO:0010556 | P | regulation of macromolecule biosynthetic process | 14 | 233 | 3366 | 52012 | 0.65 |
| GO:0006351 | P | transcription, DNA-templated | 14 | 233 | 3727 | 52012 | 0.79 |
| GO:0006518 | P | peptide metabolic process | 5 | 233 | 1802 | 52012 | 0.91 |
| GO:0032774 | P | RNA biosynthetic process | 14 | 233 | 3730 | 52012 | 0.79 |
| GO:0016310 | P | phosphorylation | 14 | 233 | 4109 | 52012 | 0.89 |
| GO:0044723 | P | single-organism carbohydrate metabolic process | 5 | 233 | 1036 | 52012 | 0.5 |
| GO:0034641 | P | cellular nitrogen compound metabolic process | 37 | 233 | 8317 | 52012 | 0.55 |
| GO:0034645 | P | cellular macromolecule biosynthetic process | 21 | 233 | 6113 | 52012 | 0.92 |
| GO:0044699 | P | single-organism process | 43 | 233 | 11479 | 52012 | 0.92 |
| GO:0006139 | P | nucleobase-containing compound metabolic process | 29 | 233 | 6446 | 52012 | 0.52 |
| GO:0006508 | P | proteolysis | 6 | 233 | 1664 | 52012 | 0.76 |
| GO:0009987 | P | cellular process | 79 | 233 | 20063 | 52012 | 0.94 |
| GO:0006725 | P | cellular aromatic compound metabolic process | 31 | 233 | 6894 | 52012 | 0.52 |
| GO:1903506 | P | regulation of nucleic acid-templated transcription | 14 | 233 | 3321 | 52012 | 0.63 |
| GO:0055085 | P | transmembrane transport | 7 | 233 | 2073 | 52012 | 0.82 |
| GO:0090304 | P | nucleic acid metabolic process | 28 | 233 | 5491 | 52012 | 0.26 |
| GO:0043603 | P | cellular amide metabolic process | 5 | 233 | 1839 | 52012 | 0.92 |
| GO:0051252 | P | regulation of RNA metabolic process | 14 | 233 | 3329 | 52012 | 0.63 |
| GO:0043170 | P | macromolecule metabolic process | 57 | 233 | 13664 | 52012 | 0.76 |
| GO:0031326 | P | regulation of cellular biosynthetic process | 14 | 233 | 3376 | 52012 | 0.66 |
| GO:0031323 | P | regulation of cellular metabolic process | 14 | 233 | 3502 | 52012 | 0.71 |
| GO:0042592 | P | homeostatic process | 5 | 233 | 396 | 52012 | 0.035 |
| GO:2000112 | P | regulation of cellular macromolecule biosynthetic process | 14 | 233 | 3366 | 52012 | 0.65 |
| GO:0071704 | P | organic substance metabolic process | 78 | 233 | 19075 | 52012 | 0.86 |
| GO:0010467 | P | gene expression | 25 | 233 | 6121 | 52012 | 0.72 |
| GO:0071702 | P | organic substance transport | 5 | 233 | 1805 | 52012 | 0.91 |
| GO:0010468 | P | regulation of gene expression | 14 | 233 | 3371 | 52012 | 0.65 |
| GO:0006468 | P | protein phosphorylation | 13 | 233 | 3669 | 52012 | 0.84 |
| GO:0044267 | P | cellular protein metabolic process | 24 | 233 | 6950 | 52012 | 0.93 |
| GO:0019219 | P | regulation of nucleobase-containing compound metabolic process | 14 | 233 | 3339 | 52012 | 0.64 |
| GO:0006464 | P | cellular protein modification process | 19 | 233 | 4679 | 52012 | 0.71 |
| GO:0009058 | P | biosynthetic process | 30 | 233 | 8504 | 52012 | 0.94 |
| GO:0009059 | P | macromolecule biosynthetic process | 21 | 233 | 6117 | 52012 | 0.92 |
| GO:0044763 | P | single-organism cellular process | 27 | 233 | 7018 | 52012 | 0.83 |
| GO:0051171 | P | regulation of nitrogen compound metabolic process | 14 | 233 | 3389 | 52012 | 0.66 |
| GO:0007154 | P | cell communication | 5 | 233 | 1521 | 52012 | 0.81 |
| GO:0009056 | P | catabolic process | 5 | 233 | 1185 | 52012 | 0.61 |
| GO:0051179 | P | localization | 18 | 233 | 5066 | 52012 | 0.88 |
| GO:0006996 | P | organelle organization | 6 | 233 | 671 | 52012 | 0.084 |
| GO:0044238 | P | primary metabolic process | 70 | 233 | 18025 | 52012 | 0.94 |
| GO:0005975 | P | carbohydrate metabolic process | 11 | 233 | 2274 | 52012 | 0.44 |
| GO:0044237 | P | cellular metabolic process | 67 | 233 | 16458 | 52012 | 0.85 |
| GO:0006796 | P | phosphate-containing compound metabolic process | 17 | 233 | 5379 | 52012 | 0.95 |
| GO:0006793 | P | phosphorus metabolic process | 17 | 233 | 5392 | 52012 | 0.96 |
| GO:0006396 | P | RNA processing | 7 | 233 | 655 | 52012 | 0.03 |
| GO:1901363 | F | heterocyclic compound binding | 69 | 233 | 18860 | 52012 | 0.99 |
| GO:0000166 | F | nucleotide binding | 41 | 233 | 11390 | 52012 | 0.95 |
| GO:0016740 | F | transferase activity | 35 | 233 | 8540 | 52012 | 0.74 |
| GO:0004672 | F | protein kinase activity | 14 | 233 | 3556 | 52012 | 0.73 |
| GO:0016818 | F | hydrolase activity, acting on acid anhydrides, in phosphorus-containing anhydrides | 9 | 233 | 2759 | 52012 | 0.87 |
| GO:0016817 | F | hydrolase activity, acting on acid anhydrides | 10 | 233 | 2832 | 52012 | 0.82 |
| GO:0097367 | F | carbohydrate derivative binding | 39 | 233 | 8366 | 52012 | 0.42 |
| GO:0016757 | F | transferase activity, transferring glycosyl groups | 11 | 233 | 1169 | 52012 | 0.018 |
| GO:0004553 | F | hydrolase activity, hydrolyzing O-glycosyl compounds | 5 | 233 | 963 | 52012 | 0.43 |
| GO:0008270 | F | zinc ion binding | 19 | 233 | 4422 | 52012 | 0.61 |
| GO:0005488 | F | binding | 128 | 233 | 34020 | 52012 | 1 |
| GO:0003676 | F | nucleic acid binding | 32 | 233 | 8571 | 52012 | 0.89 |
| GO:0003677 | F | DNA binding | 18 | 233 | 4922 | 52012 | 0.85 |
| GO:0032549 | F | ribonucleoside binding | 39 | 233 | 8189 | 52012 | 0.37 |
| GO:0017076 | F | purine nucleotide binding | 39 | 233 | 8193 | 52012 | 0.37 |
| GO:0005524 | F | ATP binding | 31 | 233 | 6846 | 52012 | 0.5 |
| GO:0016787 | F | hydrolase activity | 33 | 233 | 8107 | 52012 | 0.75 |
| GO:0016301 | F | kinase activity | 15 | 233 | 4158 | 52012 | 0.84 |
| GO:0043169 | F | cation binding | 35 | 233 | 8202 | 52012 | 0.65 |
| GO:0016788 | F | hydrolase activity, acting on ester bonds | 6 | 233 | 2096 | 52012 | 0.91 |
| GO:0003824 | F | catalytic activity | 91 | 233 | 24009 | 52012 | 0.99 |
| GO:0016758 | F | transferase activity, transferring hexosyl groups | 9 | 233 | 878 | 52012 | 0.019 |
| GO:0016773 | F | phosphotransferase activity, alcohol group as acceptor | 16 | 233 | 4173 | 52012 | 0.78 |
| GO:0016772 | F | transferase activity, transferring phosphorus-containing groups | 17 | 233 | 5055 | 52012 | 0.92 |
| GO:0032559 | F | adenyl ribonucleotide binding | 35 | 233 | 7232 | 52012 | 0.34 |
| GO:0032555 | F | purine ribonucleotide binding | 39 | 233 | 8163 | 52012 | 0.36 |
| GO:0032550 | F | purine ribonucleoside binding | 39 | 233 | 8163 | 52012 | 0.36 |
| GO:0032553 | F | ribonucleotide binding | 39 | 233 | 8311 | 52012 | 0.4 |
| GO:0035639 | F | purine ribonucleoside triphosphate binding | 35 | 233 | 7777 | 52012 | 0.52 |
| GO:0016798 | F | hydrolase activity, acting on glycosyl bonds | 5 | 233 | 1048 | 52012 | 0.51 |
| GO:0008233 | F | peptidase activity | 6 | 233 | 1440 | 52012 | 0.63 |
| GO:0016491 | F | oxidoreductase activity | 16 | 233 | 4329 | 52012 | 0.82 |
| GO:0008194 | F | UDP-glycosyltransferase activity | 5 | 233 | 345 | 52012 | 0.021 |
| GO:0043167 | F | ion binding | 36 | 233 | 8723 | 52012 | 0.73 |
| GO:0005215 | F | transporter activity | 8 | 233 | 2603 | 52012 | 0.9 |
| GO:0046872 | F | metal ion binding | 35 | 233 | 7694 | 52012 | 0.49 |
| GO:0030554 | F | adenyl nucleotide binding | 35 | 233 | 7245 | 52012 | 0.34 |
| GO:0003723 | F | RNA binding | 7 | 233 | 1378 | 52012 | 0.42 |
| GO:0005515 | F | protein binding | 47 | 233 | 12774 | 52012 | 0.95 |
| GO:0097159 | F | organic cyclic compound binding | 69 | 233 | 18864 | 52012 | 0.99 |
| GO:0001883 | F | purine nucleoside binding | 39 | 233 | 8163 | 52012 | 0.36 |
| GO:0001882 | F | nucleoside binding | 39 | 233 | 8191 | 52012 | 0.37 |
| GO:0016462 | F | pyrophosphatase activity | 8 | 233 | 2710 | 52012 | 0.92 |
| GO:1901265 | F | nucleoside phosphate binding | 41 | 233 | 11390 | 52012 | 0.95 |
| GO:0017111 | F | nucleoside-triphosphatase activity | 8 | 233 | 2615 | 52012 | 0.9 |
| GO:0036094 | F | small molecule binding | 41 | 233 | 11530 | 52012 | 0.96 |
| GO:0009055 | F | electron carrier activity | 5 | 233 | 1056 | 52012 | 0.51 |
| GO:0046914 | F | transition metal ion binding | 23 | 233 | 5835 | 52012 | 0.77 |
| GO:0070011 | F | peptidase activity, acting on L-amino acid peptides | 6 | 233 | 1379 | 52012 | 0.59 |
| GO:0044428 | C | nuclear part | 6 | 233 | 411 | 52012 | 0.011 |
| GO:0031224 | C | intrinsic component of membrane | 15 | 233 | 2581 | 52012 | 0.19 |
| GO:0016021 | C | integral component of membrane | 15 | 233 | 2545 | 52012 | 0.17 |
| GO:0016020 | C | membrane | 29 | 233 | 6593 | 52012 | 0.57 |
| GO:0043234 | C | protein complex | 15 | 233 | 2520 | 52012 | 0.16 |
| GO:0043231 | C | intracellular membrane-bounded organelle | 20 | 233 | 4042 | 52012 | 0.36 |
| GO:0044424 | C | intracellular part | 37 | 233 | 7865 | 52012 | 0.4 |
| GO:0044422 | C | organelle part | 13 | 233 | 1680 | 52012 | 0.041 |
| GO:0043232 | C | intracellular non-membrane-bounded organelle | 5 | 233 | 1875 | 52012 | 0.92 |
| GO:0043229 | C | intracellular organelle | 25 | 233 | 5670 | 52012 | 0.56 |
| GO:0005622 | C | intracellular | 40 | 233 | 9868 | 52012 | 0.78 |
| GO:0043227 | C | membrane-bounded organelle | 20 | 233 | 4042 | 52012 | 0.36 |
| GO:0012505 | C | endomembrane system | 6 | 233 | 690 | 52012 | 0.093 |
| GO:0044446 | C | intracellular organelle part | 13 | 233 | 1677 | 52012 | 0.041 |
| GO:0044444 | C | cytoplasmic part | 10 | 233 | 2741 | 52012 | 0.79 |
| GO:0005737 | C | cytoplasm | 15 | 233 | 3805 | 52012 | 0.73 |
| GO:0005634 | C | nucleus | 13 | 233 | 2916 | 52012 | 0.55 |
| GO:0032991 | C | macromolecular complex | 19 | 233 | 3839 | 52012 | 0.36 |
| GO:0044464 | C | cell part | 41 | 233 | 10436 | 52012 | 0.85 |
| GO:0005623 | C | cell | 41 | 233 | 10436 | 52012 | 0.85 |
| GO:0043228 | C | non-membrane-bounded organelle | 5 | 233 | 1875 | 52012 | 0.92 |
| GO:0043226 | C | organelle | 25 | 233 | 5670 | 52012 | 0.56 |
| GO:0044425 | C | membrane part | 20 | 233 | 3420 | 52012 | 0.14 |
