# Supplementary material for: Comparative transcriptome analysis reveals evolutionary divergence and shared network of cold and salt stress response in diploid D-genome cotton
Source: BMC Plant Biol. 2020 Nov 12;20:518. doi: 10.1186/s12870-020-02726-4 (PMC7664088; doi:10.1186/s12870-020-02726-4)
Supplement: Supplementary file 7 — Additional files 7: Table S7. Gene number of eight import modules in ten GO terms. [file 12870_2020_2726_MOESM7_ESM.docx]

Table S7 Gene number of eight import modules in ten GO terms

|  | **Yellow** | **Blue** | **Turquoise** | **Purple** | **Skyblue3** | **Mediumpurple3** | **Pink** | **Greenyellow** |
| --- | --- | --- | --- | --- | --- | --- | --- | --- |
| Total Gene Number | 420 | 1193 | 3128 | 442 | 72 | 65 | 627 | 533 |
| reproduction | 20 | 59 | 138 | 22 | 4 | 2 | 34 | 39 |
| immune system process | 32 | 100 | 277 | 41 | 5 | 16 | 46 | 51 |
| reproductive process | 90 | 229 | 537 | 80 | 22 | 20 | 149 | 127 |
| signaling | 51 | 171 | 441 | 58 | 20 | 19 | 86 | 109 |
| multicellular organismal process | 112 | 321 | 712 | 122 | 24 | 25 | 214 | 181 |
| growth | 48 | 114 | 317 | 59 | 20 | 5 | 57 | 58 |
| rhythmic process | 4 | 14 | 34 | 3 | 1 | 3 | 9 | 6 |
| response to stimulus | 189 | 569 | 1408 | 211 | 41 | 48 | 291 | 308 |
| multi-organism process | 59 | 196 | 581 | 79 | 18 | 21 | 107 | 116 |
| cellular component organization or biogenesis | 126 | 363 | 825 | 139 | 30 | 24 | 242 | 182 |
