# Supplementary figures and images for: Comparative transcriptome analysis reveals evolutionary divergence and shared network of cold and salt stress response in diploid D-genome cotton
Source: BMC Plant Biol. 2020 Nov 12;20:518. doi: 10.1186/s12870-020-02726-4 (PMC7664088; doi:10.1186/s12870-020-02726-4)

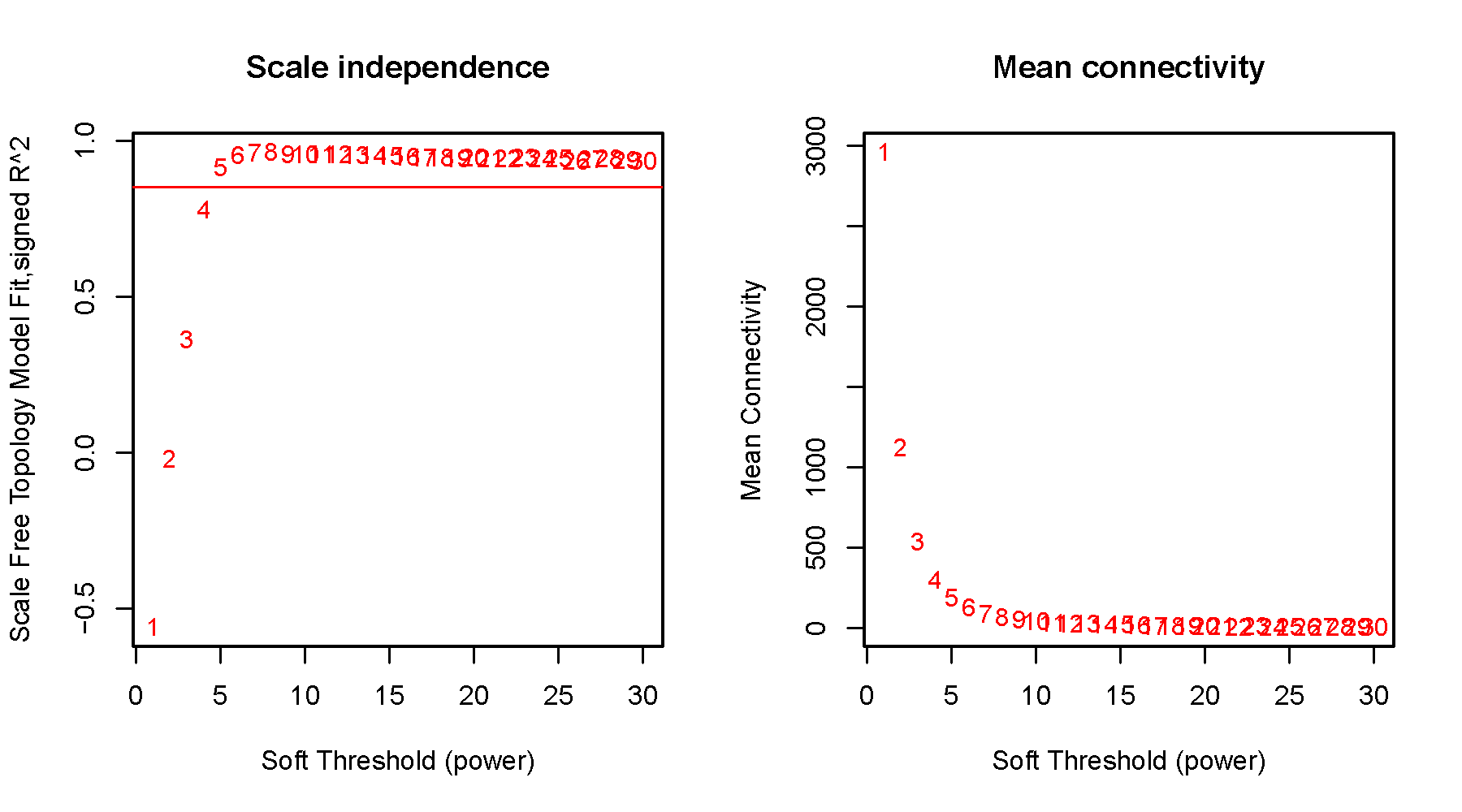

Supplement: Supplementary file 8 — Additional files 8: Figure S1. Determination of soft-thresholding power. (A) Analysis of the scale-free fit index for various soft-thresholding powers (β). (B) Analysis of the mean connectivity for various soft-thresholding powers. [file 12870_2020_2726_MOESM8_ESM.tiff]

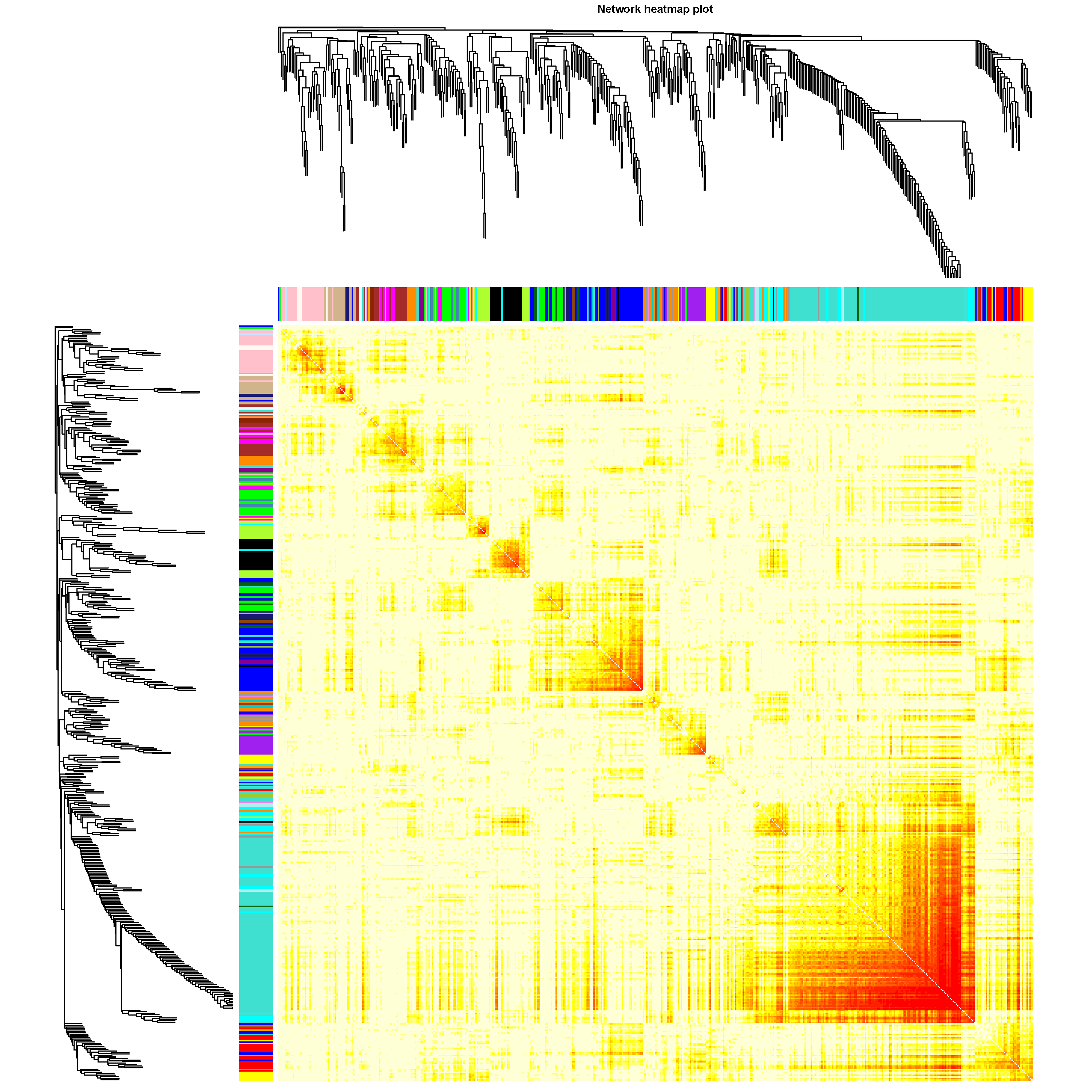

Supplement: Supplementary file 9 — Additional files 9: Figure S2. Heatmap plot of the topological overlap matrix. [file 12870_2020_2726_MOESM9_ESM.tiff]

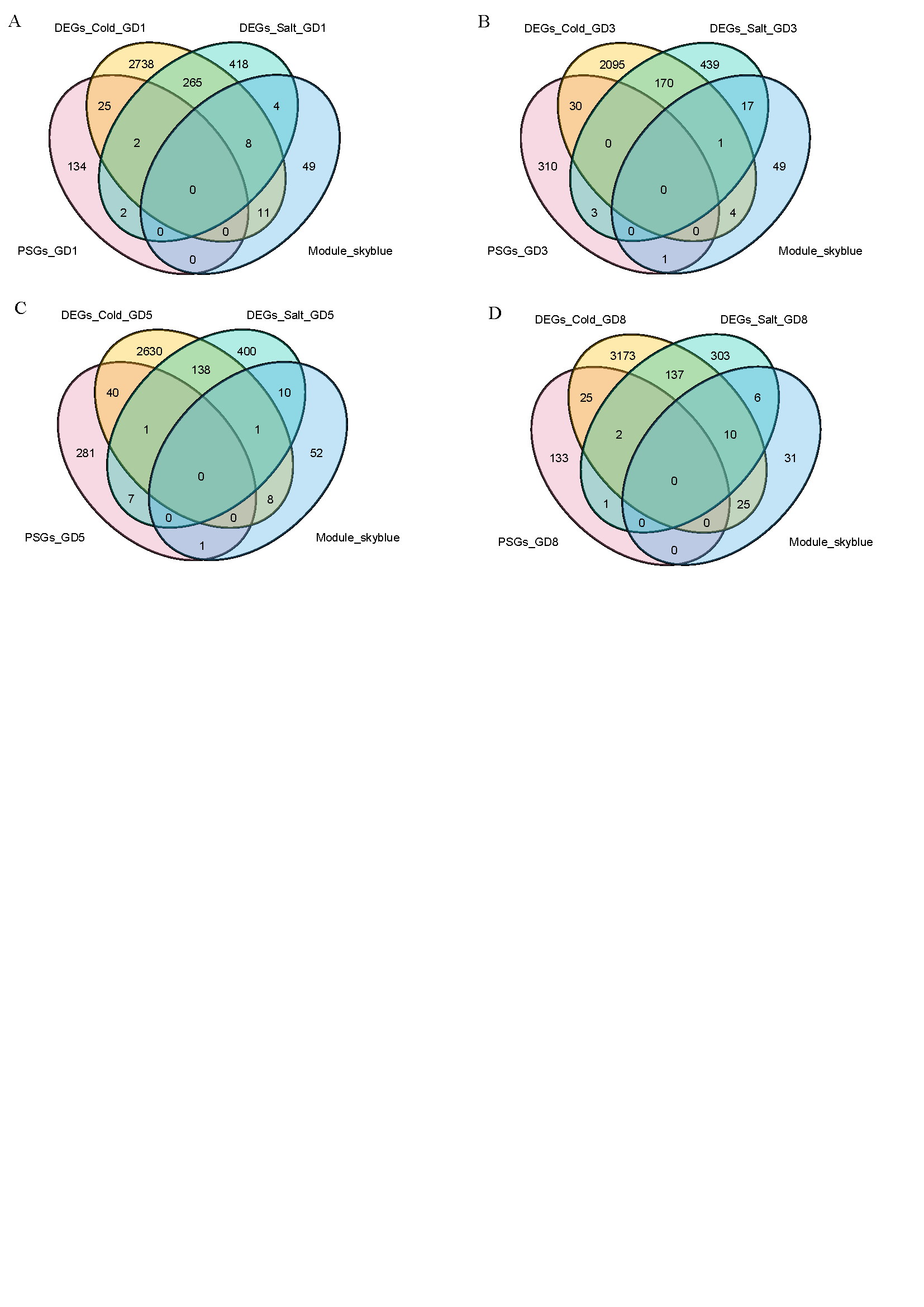

Supplement: Supplementary file 10 — Additional files 10: Figure S3. Venn diagrams of DEGs; PSGs and genes of skyblue3 module. A-D represents the Venn diagram of GD1, GD3, GD5, and GD8, respectively. [file 12870_2020_2726_MOESM10_ESM.tiff]
